# Association between Antibiotic Prescribing in Pregnancy and Cerebral Palsy or Epilepsy in Children Born at Term: A Cohort Study Using The Health Improvement Network

**DOI:** 10.1371/journal.pone.0122034

**Published:** 2015-03-25

**Authors:** Wilhelmine Hadler Meeraus, Irene Petersen, Ruth Gilbert

**Affiliations:** 1 UCL Institute of Child Health, Population Policy and Practice Programme, London, United Kingdom; 2 UCL Research Department of Primary Care and Population Health, London, United Kingdom; 3 Farr Institute of Health Informatics Research, London, United Kingdom; Nathan Kline Institute and New York University School of Medicine, UNITED STATES

## Abstract

**Background:**

Between 19%-44% pregnant women are prescribed antibiotics during pregnancy. A single, large randomised-controlled-trial (ORACLE Childhood Study II) found an increased risk of childhood cerebral palsy and possibly epilepsy following prophylactic antibiotic use in pregnant women with spontaneous preterm labour. We ascertained whether this outcome could be reproduced across the population of babies delivered at term and prospectively followed in primary-care using data from The Health Improvement Network.

**Methods:**

We determined the risk of cerebral palsy or epilepsy in children whose mothers were prescribed antibiotics during pregnancy using a cohort of 195,909 women linked to their live, term-born, singleton children. We compared the effect of antibiotic class, number of courses and timing of prescribing in pregnancy. Analyses were adjusted for maternal risk factors (e.g. recorded infection, age, chronic conditions, social deprivation, smoking status). Children were followed until age seven years or cessation of registration with the primary-care practitioner.

**Results:**

In total, 64,623 (33.0%) women were prescribed antibiotics in pregnancy and 1,170 (0.60%) children had records indicating cerebral palsy or epilepsy. Adjusted analyses showed no association between prescribing of any antibiotic and cerebral palsy or epilepsy (adj.HR 1.04, 95%CI 0.91–1.19). However, compared with penicillins, macrolides were associated with an increased risk of cerebral palsy or epilepsy (adj.HR 1.78, 95%CI 1.18–2.69; number needed to harm 153, 95%CI 71–671).

**Conclusions:**

We found no overall association between antibiotic prescribing in pregnancy and cerebral palsy and/or epilepsy in childhood. However, our finding of an increased risk of cerebral palsy or epilepsy associated with macrolide prescribing in pregnancy adds to evidence that macrolide use is associated with serious harm.

## INTRODUCTION

Antibiotics are one of the most frequently prescribed medications in pregnancy, with estimates of antibiotic use in pregnancy ranging from 19% to 44%.[1–6] When deciding whether to taken any form of pharmacologic treatment during pregnancy, women (and the healthcare professionals treating them) need to be sure that overall benefits outweigh potential harm to the fetus.[7,8] There is strong evidence for net benefits of prenatal antibiotics for mother and child when infection is the predominant underlying mechanism of effect. For example, prenatal antibiotics are effective for women with suspected bacterial infections (e.g urinary tract infections [UTI][9]) and as prophylaxis for women with asymptomatic bacteriuria[10] and preterm prolonged rupture of membranes.[11]

Unexpected evidence of harm associated with prenatal antibiotics was provided by a single large (N>4,000) randomised-controlled-trial (RCT) of prophylactic antibiotics for women in spontaneous preterm labour.[12] This trial, the Oracle Childhood Study (OCS) II, evaluated children at the age of seven years whose mothers had been randomised to receive erythromycin, co-amoxiclav, both, or placebo during pregnancy. Most children were born at term and were delivered greater than seven days after trial entry.[12] Women who received both erythromycin and co-amoxiclav had an increased risk of having a child with cerebral palsy (odds ratio [OR]: 2.93; 95% confidence interval [CI]: 1.50–5.65) or epilepsy (OR: 1.53; 95%CI: 1.07–2.20) compared to women randomised to receive placebo.[12–14] These findings may be explained by harm due to antibiotics, possibly from prolonged exposure to inflammatory processes in the intrauterine environment due to partially treated infection.[15] Alternatively, the apparent adverse effect of antibiotics may be explained by biases due to ‘damaged-survivors’ in analyses that were conditioned on survivors of infancy—where stillbirths, infant deaths and children lost-to-follow up were excluded.[16]

Antibiotics are now not recommended for women in preterm labour without overt evidence of infection and further RCTs to confirm or refute the findings of OCS II are unlikely.[17] Large, longitudinal primary-care databases are widely used to investigate drug safety and are particularly useful for exploring unexpected adverse effects.[18] We therefore analysed primary-care data to determine whether antibiotic prescribing (for any indication) during pregnancy was associated with cerebral palsy or epilepsy in children and whether risk of cerebral palsy or epilepsy varied according to the timing, number of courses, and class of antibiotic prescribed. We hypothesised that there would be no association between prenatal antibiotic prescribing and cerebral palsy and/or epilepsy in childhood.

## METHODS

### Data Source

We used The Health Improvement Network (THIN) which captures prospective, anonymised data on prescriptions, diagnoses, and symptoms in primary-care for approximately 6% of the United Kingdom population.[19] Prescription data are automatically recorded when prescriptions are issued, and they are classified according to the British National Formulary (BNF).[20,21] Significant medical events are recorded by clinicians and coded using the Read system.[22] Data on contact with secondary-care and socio-demographic status are also available. THIN is broadly representative of the UK population and captures valid and complete data.[23–27]

### Study design

We analysed a retrospective cohort of women who were linked to their live-born children using deterministic (exact) linkage methods. The following variables were considered when linking mothers and children: family ID number, mother’s actual or estimated date of delivery, child’s month and/or year of birth, and gestational age at delivery/birth.

### Participants

Inception to the cohort was restricted to women aged 15–50 years between January 1990 and May 2010, who had registered at their primary-care practice before pregnancy and who had a singleton, child born at or after term (37 weeks of gestation or more). Where gestational age at delivery was uncertain and evidence of pre- or post-term birth was lacking (37%), we assumed pregnancy lasted 40 weeks. For women with multiple pregnancies (approximately 24% in the cohort), we randomly selected one pregnancy for analysis. Children born preterm were excluded because their increased risk of cerebral palsy and epilepsy (related to prenatal, perinatal and postnatal risk factors) could mask any association with antibiotic prescribing in pregnancy. For consistency with the OCS II study[12], follow-up ended at the earliest of seven completed years of age, the date the child left their primary-care practice, death, or the end of the study (May 2010).

### Antibiotic prescriptions

We considered women as treated if they had a prescription record between the last menstrual period and estimated delivery date for any antibiotic in chapter 5.1 of the British National Formulary (BNF), excluding those with topical formulations such as antibiotics used to treat acne (BNF chapter 13.6.1) and conjunctivitis (BNF chapter 11.3.1). A list of the generic antibiotics considered is provided in **S1 Appendix—Codes used to identify women with antibiotic prescriptions in pregnancy**. All other women were considered as untreated. Antibiotics prescribed and dispensed by hospitals are not captured in THIN, but are likely to affect very few women in this study. Except for women whose illness or labour requires hospital care, treatment would normally be prescribed by the primary-care practitioner. Intrapartum antibiotic prescribing has been estimated to involve less than 5% of women, including those delivering preterm.[28]

### Cerebral palsy or epilepsy

The pre-specified primary outcome was cerebral palsy and/or epilepsy recorded in a child’s primary-care record up to seven years old. We grouped cerebral palsy and epilepsy for the following reasons: 1. they often co-occur; 2. they have common prenatal aetiologies; 3. they were associated with prenatal antibiotic prescribing in the OCS II study; and 4. to increase statistical power for our analyses of timing, number of courses and type of antibiotic prescribed.

We used a previously published and validated algorithm to identify children with epilepsy which was based on one or more codes for an epilepsy diagnosis, symptoms (multiple seizure records), and on-going treatment (repeated prescriptions for anti-epileptic drugs).[29] Further details, and code lists, are provided in **S2 Appendix—Description of algorithm and codes used to identify children with epilepsy**. A similar algorithm was developed to identify children with cerebral palsy, whereby codes for diagnoses, symptoms (e.g. spasticity), treatment (e.g. intramuscular injection of baclofen) and management procedures (e.g. tenotomy) were used to identify children with possible cerebral palsy. We then reviewed the full medical records of all children with possible cerebral palsy and excluded (from consideration as a child with cerebral palsy) those without consistent evidence of a non-progressive motor disorder. All reviewers were blinded as to whether a child’s mother was prescribed antibiotics during pregnancy. Additional details are available in **S3 Appendix—Description of algorithm and codes used to identify children with cerebral palsy**.

The outcome date was the earliest date upon which evidence for cerebral palsy or epilepsy was recorded.

### Maternal risk factors for both antibiotic prescribing in pregnancy and cerebral palsy and/or epilepsy in the child

We classified the following maternal risk factors: maternal age at delivery, pregnancy complications (e.g. any record indicating hypertension or diabetes), chronic conditions (e.g. obesity prior to pregnancy [BMI≥30 or Read code for obesity], treatment for chronic medical conditions during pregnancy)[30], behaviours (e.g. recent smoking/tobacco use, alcohol or illicit drug misuse at any time), social deprivation (measured by the Townsend score quintile where Q1 is most affluent and Q5 is most deprived [31]), maternal infection that was a potentially neurologically-damaging infection (PNDI) for the fetus (e.g. UTIs and chorioamnionitis; see **S4 Appendix—Read codes used to identify women with potentially neurologically-damaging infections in pregnancy**).[32,33] These maternal risk factors were chosen a priori based on evidence from the literature of associations between these factors and both our exposure and outcomes of interest. We assumed that absence of codes for maternal conditions indicated absence of those conditions.

### Analysis

We used a series of Cox regression models to compare the time to cerebral palsy and/or epilepsy in different groups of women who were and were not prescribed antibiotics in pregnancy. In the primary analysis we compared children whose mothers received one or more antibiotic prescriptions with those whose mothers had no recorded antibiotic prescription. Adjustment was made for potential maternal confounders using the propensity score for antibiotic prescribing. This was derived using all maternal risk factors (for antibiotic prescribing and for having a child with cerebral palsy and/or epilepsy) listed previously as well as calendar year of delivery.[34] Ten women with non-overlapping propensity score distributions were trimmed from the analysis cohort. We further adjusted this and all other analyses for potential clustering at the level of primary-care practice by using robust standard errors. Assumptions of proportional hazards were met for this and all other models.

In a sensitivity analysis (designed to minimise bias due to the type of infection for which antibiotics were prescribed—not to minimise bias due to infection severity) we repeated the above analysis using a restricted cohort which included women with a single respiratory tract infection recorded in pregnancy and compared children whose mothers received an antibiotic prescription for a respiratory tract infection with children whose mothers were not prescribed antibiotics for their respiratory tract infection. Women with respiratory tract infections in pregnancy were identified using Read codes—details are provided in **S5 Appendix—Read codes used to identify women with respiratory tract infections in pregnancy**.

In secondary analyses we determined the effect of number, timing and type of antibiotic prescriptions in pregnancy on the time to cerebral palsy and/or epilepsy. To assess the effect of number of courses of antibiotics prescribed (where each separate prescription was defined as a single course), we categorised the variable for antibiotic treatment into a four-level variable (none, one, two or three or more courses). We determined the association with the class (type) and timing of antibiotic prescription in analyses restricted to women prescribed a single course of antibiotics. Class of antibiotic was grouped according to BNF sub-chapters (e.g. penicillins, aminoglycosides, macrolides, tetracyclines). Pencillins were chosen as the referent group because they were the most commonly prescribed class of antibiotics in pregnancy and have the longest history of safe use in pregnancy. To assess timing, we categorised prescriptions within four week periods (lunar month) of gestational age. Here, the first lunar month was arbitrarily chosen as the referent group. In all secondary analyses, we accounted for potential confounding using a stepwise approach to model building, as propensity scores are only valid with binary exposure variables. Numbers needed to harm were estimated at age seven years using methods of Altman et al.[35]

Finally, in post-hoc analyses requested by a reviewer, we repeated the primary analysis (any versus no antibiotic) and one secondary analysis (class of antibiotic versus penicillins) on risk of epilepsy.

All authors designed the study. WM performed the analyses, with input and advice from RG. Code lists were reviewed by GPs, a paediatrician (RG), and an epileptologist.

### Ethics Statement

Research using data from THIN has blanket ethics approval from the South East Multi-Centre Research Ethics Committee. All data in THIN are anonymised and researchers do not have access to any identifiable information such as names, NHS numbers, postcodes or full birthdays. This study received additional approval from the THIN scientific review committee (13_008).

## RESULTS

We identified a cohort of 195,909 women linked to their singleton children born at or after term. The cohort comprised all mother-child pairs in THIN meeting the study eligibility criteria. The children were followed up from birth for a median of 3.6 years (inter quartile range [IQR] 1.5–7.2). Median follow-up differed by outcome status—children with cerebral palsy and/or epilepsy were followed-up for 6.5 years (IQR 3.5–10.3) whilst children without were followed-up for 3.7 years (IQR 1.6–7.2). In total, 1,170 (0.6%) children had evidence of cerebral palsy (n = 232), epilepsy (n = 874) or both (n = 64) in their medical records, and 64,623 (33%) were prescribed antibiotics during pregnancy.

Compared to women not prescribed antibiotics, women prescribed antibiotics (Table 1) were on average more deprived, more likely to be obese (15.5% vs 10.9%), to receive treatment for chronic medical conditions in pregnancy (14.9% vs 9.2%), to have problems with alcohol (1.7% vs 1.1%) to use tobacco (28.6% vs 21.5%) or illicit drugs (1.6% vs 0.9%), and to have an infection which could potentially cause fetal neuro-damage (23.2% vs 4.2%).

**Table 1:** Characteristics of women in the cohort.

Variable		Number (%) without an antibiotic prescription recorded (n = 131,286)		Number (%) with an antibiotic prescription recorded (n = 64,623)	
All		131,286	(67.0%)	64,623	(33.0%)
Obese prior to pregnancy		14,371	(10.9%)	10,011	(15.5%)
Illicit drug use		1,190	(0.9%)	1,020	(1.6%)
Current smoking / tobacco use		28,209	(21.5%)	18,457	(28.6%)
Alcohol problems		1,488	(1.1%)	1,101	(1.7%)
Treatment for chronic medical condition in pregnancy		12,023	(9.2%)	9,633	(14.9%)
Social deprivation Townsend quintile based on postcode of residence)	1 (most affluent)	34,699	(26.4%)	14,250	(22.1%)
	2	27,322	(20.8%)	12,031	(18.6%)
	3	27,303	(20.8%)	13,407	(20.7%)
	4	24,683	(18.8%)	13,744	(21.3%)
	5 most deprived)	17,279	(13.2%)	11,191	(17.3%)
Age at delivery	15–19	6,196	(4.7%)	4,429	(6.9%)
	20–24	18,318	(14.0%)	11,668	(18.1%)
	25–29	34,756	(26.5%)	17,367	(26.9%)
	30–34	42,868	(32.7%)	18,791	(29.1%)
	35–39	24,054	(18.3%)	10,227	(15.8%)
	40–50	5,094	(3.9%)	2,141	(3.3%)
Calendar year of delivery	1990–94	7,383	(5.6%)	3,629	(5.6%)
	1995–99	17,501	(13.3%)	9,051	(14.0%)
	2000–04	41,848	(31.9%)	19,031	(29.4%)
	2005–10	64,554	(49.2%)	32,912	(50.9%)
Diabetes in pregnancy		2,944	(2.2%)	1,902	(2.9%)
Hypertension in pregnancy		4,380	(3.3%)	2,499	(3.9%)
Potentially neurologically-damaging infection in pregnancy		5,538	(4.2%)	14,978	(23.2%)

*NB. Absence of information on obesity, illicit drug use, smoking, alcohol problems, treatment for chronic medical conditions, diabetes and hypertension in a mothers’ medical record was interpreted as absence of the condition*.

Unadjusted analyses showed an increased risk of cerebral palsy and/or epilepsy in children born to women prescribed antibiotics compared with women with no recorded prescriptions (Table 2: unadj. hazard ratio [HR] 1.20; 95%CI 1.06–1.35; p = 0.004). However, after adjusting for potential confounders, no significant association was evident (adj.HR 1.04; 95% CI 0.91–1.19; p = 0.5). In sensitivity analyses restricted to women with a single respiratory tract infection in pregnancy, no association (significant at the 5% level) was found between antibiotic prescribing and cerebral palsy or epilepsy (Table 2). The post-hoc analysis for epilepsy also showed no significant association between any antibiotic prescribing during pregnancy and the risk of epilepsy in childhood, after adjusting for potential confounders (adj.HR 1.03; 95%CI 0.89–1.10; p = 0.7).

**Table 2:** Women prescribed antibiotics versus never prescribed: time to cerebral palsy and/or epilepsy in childhood.

	Number mother-child pairs in analysis	Number (%) prescribed antibiotics		Number children with any cerebral palsy or epilepsy	Unadjusted hazard ratio (95% confidence interval)		Adjusted† hazard ratio (95% confidence interval)	
All women	195,899	64,613	(33.0%)	1,170	1.20*	(1.06, 1.35)	1.04	(0.91, 1.19)
Women with a single respiratory tract infection‡	17,822	7,266	(40.8%)	83	0.99	(0.65, 1.51)	0.94	(0.62, 1.42)

*p<0.01

*† Cox regression model adjusted for propensity score which included: hypertension, smoking/tobacco use, diabetes, age at delivery, year of delivery, Townsend quintile, treatment of chronic medical condition in pregnancy, alcohol problems, illicit drug use, obesity and potentially neurologically-damaging infection during pregnancy*.

*‡ Analysis cohort included women with a single respiratory tract infection who either received or did not receive a single antibiotic prescription within plus or minus three days of the recorded date of respiratory tract infection*.

Whilst we found no association with the timing of antibiotic prescription and cerebral palsy or epilepsy, we found that children whose mothers received 3+ prescriptions during pregnancy had a 40% increased risk (adj.HR 1.40; 95%CI 1.07–1.83; p = 0.01) compared to those with no prescriptions (Table 3). The incidence of cerebral palsy or epilepsy in children whose mothers received 3+ prescriptions in pregnancy was 261.1 (95%CI 210.5–323.7) per 100,000 child-years-at-risk (CYAR) compared to 143.8 (95%CI 133.8–154.5) in children whose mothers received no prescriptions. The number needed to harm was 291 (95%CI 141–191) at age seven years.

**Table 3:** Association between class of antibiotic and number and timing of antibiotic prescriptions in pregnancy with cerebral palsy and/or epilepsy in childhood.

		Number (%) of women		Number of children with cerebral palsy and/or epilepsy	Follow-up in child-years-at-risk (CYAR)	Incidence rate of cerebral palsy and/or epilepsy per 100,000 CYAR (95% confidence interval)		Unadjusted hazard ratio (95% confidence interval)		Adjusted*†‡* hazard ratio (95% confidence interval)	
TYPE†—Group of antibiotic prescribed (n = 41,276 women with single prescriptions for most commonly prescribed antibiotics in pregnancy)	Penicillins	27,577	(66.8%)	156	108,630	143.6	(122.8, 168.0)	[Ref]		[Ref]	
	Cephalosporins, carbapenems, and other beta-lactams	7,740	(18.8%)	40	28,327	141.2	(103.6, 192.5)	0.96	(0.68, 1.36)	0.99	(0.69, 1.41)
	Sulphonamides and trimethoprim	1,375	(3.3%)	7	5,169	135.4	(64.6, 284.1)	0.93	(0.45, 1.95)	0.89	(0.43, 1.85)
	Nitrofurantoin	802	(1.0%)	6	2,900	206.9	(93.0, 460.5)	1.42	(0.65, 3.13)	1.31	(0.58, 2.92)
	Metronidazole and tinidazole	1,033	(2.5%)	9	3,987	225.7	(117.4, 433.8)	1.56	(0.82, 2.99)	1.5	(0.78, 2.88)
	Macrolides	2,749	(6.7%)	28	11,012	254.3	(175.6, 368.3)	1.77**	(1.18, 2.68)	1.78**	(1.18, 2.69)
NUMBER‡—No. antibiotic prescriptions (n = 195,909 women with any number of prescriptions)	0	131,286	(67.0%)	739	514,030	143.8	(133.8, 154.5)	[Ref]		[Ref]	
	1	42,065	(21.5%)	252	163,203	154.4	(136.5, 174.7)	1.07	(0.92, 1.25)	0.98	(0.84, 1.14)
	2	14,166	(7.2%)	96	54,385	176.5	(144.5, 215.6)	1.22	(1.00, 1.50)	1.04	(0.84, 1.29)
	3+	8,392	(4.3%)	83	31,792	261.1	(210.5, 323.7)	1.80***	(1.41, 2.31)	1.40*	(1.07, 1.83)
TIMING†—by lunar month of antibiotic prescription (n = 42,065 women with single prescriptions)	M1	4,182	(9.9%)	24	16,452	145.9	(97.8, 217.6)	[Ref]		[Ref]	
	M2	3,689	(8.8%)	18	13,888	129.6	(81.7, 205.7)	0.88	(0.47, 1.65)	0.89	(0.47, 1.67)
	M3	3,655	(8.7%)	22	13,591	161.9	(106.6, 245.8)	1.09	(0.63, 1.90)	1.12	(0.64, 1.94)
	M4	4,387	(10.4%)	23	16,746	137.3	(91.3, 206.7)	0.93	(0.51, 1.73)	0.95	(0.51, 1.76)
	M5	4,356	(10.4%)	19	16,819	113	(72.1, 177.1)	0.77	(0.43, 1.38)	0.78	(0.43, 1.42)
	M6	4,227	(10.0%)	31	16,345	189.7	(133.4, 269.7)	1.3	(0.76, 2.22)	1.3	(0.75, 2.23)
	M7	4,246	(10.1%)	31	16,757	185	(130.1, 263.1)	1.27	(0.76, 2.13)	1.29	(0.77, 2.16)
	M8	4,222	(10.0%)	35	16,580	211.1	(151.7, 294.0)	1.45	(0.89, 2.34)	1.45	(0.89, 2.36)
	M9	4,434	(10.5%)	26	17,515	148.4	(101.1, 218.0)	1.02	(0.58, 1.79)	1.03	(0.58, 1.82)
	M10	4,667	(11.1%)	23	18,511	124.3	(82.6, 187.0)	0.85	(0.48, 1.53)	0.87	(0.48, 1.56)

* p<0.05

** p<0.01

*** p<0.001

*† Analyses on TYPE and TIMING of prescription—Cox regression models adjusted for year of delivery, Townsend quintile, and potentially neurologically-damaging infection recorded during pregnancy*.

*‡ Analysis on NUMBER of prescriptions—Cox regression model adjusted for maternal age, Townsend quintile, year of delivery, smoking/tobacco use, alcohol problems, obesity, illicit drug use, treatment of chronic medical conditions and potentially neurologically-damaging infection during pregnancy*.

Children whose mothers were prescribed macrolides had a 78% increased risk (adj.HR 1.78; 95%CI 1.18–2.69; p = 0.006) compared to those prescribed penicillins (Table 3). The incidence of cerebral palsy or epilepsy in children whose mothers were prescribed macrolides was 254.6 per 100,000 CYAR (95%CI 175.6–368.3) compared to 143.6 (95%CI 122.8–169.0) in children whose mothers were prescribed penicillin. The number need to harm was 153 (95%CI 71–671) at age seven years. The post-hoc analysis which looked at epilepsy as the outcome, found that macrolide versus penicillin prescribing in pregnancy was associated with a doubling in the risk of epilepsy in childhood (adj.HR 2.02; 95%CI 1.30–3.14; p = 0.002).

## DISCUSSION

We found no evidence for an increased risk of cerebral palsy and/or epilepsy in children of women prescribed any antibiotics in pregnancy compared with no antibiotics after taking into account maternal risk factors for treatment. Children of women prescribed three courses or more of antibiotics compared with none did have an increased risk of cerebral palsy or epilepsy which may reflect the risks associated with recurrent infections. We also found an increased risk of cerebral palsy or epilepsy in children of women prescribed macrolides compared with penicillins.

Strengths of our study are the large cohort, the fact that THIN captures all primary-care prescribing (though not dispensing), and the longitudinal records allowing follow up into childhood. One weakness, inherent in observational studies, is confounding of the relationship between antibiotic prescribing and childhood cerebral palsy or epilepsy by maternal risk factors (such as parity which is not reliably recorded in UK primary care), and particularly by maternal infections that indicated antibiotic treatment.

We adjusted analyses for maternal risk factors including maternal infections for which there was evidence of an association with fetal neuro-damage (PNDI). We considered maternal infection to be a confounder in the analyses as there is evidence from numerous studies that certain maternal infections cause fetal neurological damage[32,33] and mothers with infections are more likely to receive antibiotics. Failure to adjust for maternal infections can produce spurious associations that reflect the infection not the treatment. For example, two Danish population-based studies used antibiotic prescribing in pregnancy as a proxy for maternal infection. They reported associations between prenatal antibiotic prescribing and adverse neurological outcome in childhood, which are likely to reflect adverse effects of infections in pregnancy.[36,37] We also endeavoured to minimise the potential adverse effects of antibiotics due to their beneficial effects on infection by conducting a pre-planned sensitivity analysis restricted to women with ‘treated’ and ‘untreated’ respiratory tract infections. Because respiratory tract infections diagnosed in primary-care are usually of viral aetiology[38], any benefits due to antibiotic treatment of bacterial respiratory tract infection would be small or negligible, thereby increasing the chance of detecting potential adverse effects of antibiotic treatment. However, we were unable to measure infection severity—more severe infections would be linked to adverse fetal effects but would be more likely to be treated, thereby introducing bias in favour of an apparent harmful effect of antibiotics. Our comparison of different classes of antibiotics reduced confounding due to the indication for antibiotic treatment (i.e. infection) whereby an apparent association between prenatal antibiotics and adverse neurological outcome in childhood could result from the underlying infection which is a cause for both the antibiotic treatment and the brain damage manifesting as cerebral palsy and epilepsy. By examining only patients with an antibiotic prescription (and thus a likely infection) we were able to minimise such indication bias. This was particularly so for the comparison of macrolides versus penicillins as macrolides are used as alternative treatment for the same indications in penicillin-allergic patients.[21]

Another limitation of the study is the measurement of antibiotic exposure. Firstly, we could not capture antibiotics prescribed by maternity services. The prevalence of antibiotics prescribed intrapartum and not recorded in primary-care is estimated to be less than 5% of pregnancies[28] but is not routinely documented in national data. Misclassification of women treated by maternity services as untreated in primary-care would likely have had negligible effect due to small numbers among women delivering at or after term. Secondly, we could not measure which women actually had their prescriptions dispensed and which women took antibiotics as prescribed. Few studies have quantified non-compliance in pregnancy, though this may be as high as 39%.[39]

Our two-step approach to identifying children with cerebral palsy and epilepsy combined a sensitive algorithm with manual review of full medical records to ensure we only classified cases based on patterns of care in the longitudinal record that were consistent with cerebral palsy or epilepsy. Our criteria were highly specific so it is possible that we missed some cases. However, insensitive measurement of the outcome would not bias the relative effect.[40] Because antibiotics are frequently used in pregnancy, prescribing of antibiotics in primary-care is unlikely to have an impact on ascertainment of cerebral palsy or epilepsy and also unlikely to bias length of follow-up with the primary-care practice.

Using a composite outcome gave our analyses greater statistical power which enabled us to explore the impact of timing, number of courses and type of antibiotic prescribed. Disadvantages of using a composite outcome include the difficulty of determining specificity of adverse effects—for cerebral palsy and/or epilepsy separately. Our post-hoc analyses suggest the findings apply to epilepsy, but further studies are needed to explore a range of neurological outcomes.

### Mechanisms

Our study was not designed to replicate the OCS II which focused on women in spontaneous preterm labour. However, it is intriguing that we found a similar association between maternal macrolide prescribing and cerebral palsy or epilepsy in children born at term. We propose several potential mechanisms for adverse effects of prenatal antibiotics—possibly arising from biases due to failure to account for ‘damaged-survivors’ and possibly through direct effects of macrolides. However, we would be cautious to draw a causal effect based on observational data.

First, prenatal antibiotic treatment could shift the spectrum of fetal neurological-damage caused by infection towards less severe outcomes—the ‘damaged-survivor’ hypothesis. This mechanism could manifest by increased survival and less severe neurological manifestations in survivors of treated versus untreated women. In OCS II, a shift may have affected a minority of infected and neurologically-damaged fetuses, resulting in insufficient imbalance in numbers of stillbirths, postnatal deaths or losses to follow-up (likely to be higher in more severely affected survivors[41]), to reach statistical significance. A similar selection bias may have happened in our cohort study and may have masked any benefits of prenatal antibiotics.

Second, partially treated infection may prolong exposure of the fetal brain to inflammation, thereby increasing the risk of neuro-damage. This mechanism could be specific to macrolides if women stopped taking erythromycin because of gastrointestinal side-effects,[42,43] or because of high rates of treatment failure from macrolide-resistant bacteria[44] or due to poor transplacental passage of macrolide antibiotics (in comparison to ampicillin, [45,46] the most commonly prescribed penicillin in pregnancy in UK primary-care[47]).

Third, macrolides may have a specific adverse effect on the fetus. There is a growing body of evidence that macrolides are associated with adverse outcomes—including cardiovascular events (including death) in adults [48–50], cardiotoxicity[51], and miscarriage[52]. Some studies have observed an association with macrolides and birth defects[53–55], whilst others have not [56,57]. The exact mechanism for harm is unclear, though arrhythmia resulting from inhibition of a cardiac potassium channel (IKr) and prolongation of the QT interval may play a role.[58] In animal studies, drugs which block the IKr channel (e.g. macrolide antibiotics[51]) cause embryonic bradycardia, arrhythmia and cardiac arrest, even at concentrations that do not affect the maternal heart.[59] The resulting temporary hypoxia (from interrupted/decreased oxygen supply) causes tissue damage.[60] Hypoxia in the first trimester is teratogenic[61], resulting in an increased risk of transverse limb deficiencies and cardiac anomalies with IKr-blocking drugs (e.g. macrolides[53,54], clomipramine and paroxetine[62], phenytoin[61]). In addition to teratogenic effects, hypoxia is associated with failure of the fetus to grow and thrive in the uterus,[63] which is associated with cerebral palsy.[64] Hypoxia, in association with ischaemia and/or infection, is also thought to play a role in the aetiology of cerebral palsy and epilepsy.[65–67]

### Conclusions

We found no overall association between prescribing of any antibiotics during pregnancy and cerebral palsy or epilepsy in childhood. However, we found that prenatal prescribing of macrolides versus penicillins was associated with an increase in the relative risk of cerebral palsy or epilepsy in childhood, though the absolute risk remained low. This finding of harm associated with macrolide use in pregnancy is consistent with findings from the OCS II trial and with findings of adverse effects in other patient groups.

## Supporting Information

S1 Appendix Codes used to identify women with antibiotic prescriptions in pregnancy. (DOCX)

S2 Appendix Description of algorithm and codes used to identify children with epilepsy. (DOCX)

S3 Appendix Description of algorithm and codes used to identify children with cerebral palsy. (DOCX)

S4 Appendix Read codes used to identify women with potentially neurologically-damaging infections in pregnancy. (DOCX)

S5 Appendix Read codes used to identify women with respiratory tract infections in pregnancy. (DOCX)
