# Supplementary material for: Association between Antibiotic Prescribing in Pregnancy and Cerebral Palsy or Epilepsy in Children Born at Term: A Cohort Study Using The Health Improvement Network
Source: PLoS One. 2015 Mar 25;10(3):e0122034. doi: 10.1371/journal.pone.0122034 (PMC4373729; doi:10.1371/journal.pone.0122034)
Supplement: S1 Appendix — (DOCX) [file pone.0122034.s001.docx]

**S1 Appendix – Encrypted multiplex codes used to identify women with antibiotic prescriptions in pregnancy.**

*NB. Encrypted multiplex codes were were used in The Health Improvement Network (THIN) to classify medicinal products and devices prior to their switch to Gemscript codes.*

| **Code** | **Description** |
| --- | --- |
| 82568998 | GENTAMICIN crm 0.1% |
| 83215998 | TOBRAMYCIN neb soln 300mg/4ml |
| 83216998 | TOBRAMYCIN neb soln 300mg/4ml |
| 83975998 | TOBRAMYCIN inj 80mg/2ml |
| 84108998 | NEOMYCIN SULPHATE oral liq |
| 84622998 | GENTAMICIN implant 225mg |
| 84623998 | GENTAMICIN implant 75mg |
| 84625998 | GENTAMICIN implant 225mg |
| 84626998 | GENTAMICIN implant 75mg |
| 84679998 | GENTAMICIN oral liq |
| 85191998 | TOBRAMYCIN inj 240mg/6ml |
| 85192998 | TOBRAMYCIN inj 80mg/2ml |
| 85193998 | TOBRAMYCIN inj 40mg/1ml |
| 85726998 | GENTAMICIN inj 80mg/2ml |
| 85727998 | GENTAMICIN inj 40mg/1ml |
| 85892998 | NETILMICIN inj 100mg/1ml |
| 85893998 | NETILMICIN inj 100mg/1ml |
| 85894998 | NETILMICIN inj 200mg/2ml |
| 85895998 | NETILMICIN inj 200mg/2ml |
| 85896998 | NETILMICIN inj 150mg/1.5ml |
| 85897998 | NETILMICIN inj 150mg/1.5ml |
| 87362998 | GENTAMICIN inj 80mg/2ml |
| 88186998 | NEOMYCIN SULPHATE pwdr |
| 89348998 | TOBRAMYCIN neb soln 300mg/5ml |
| 92560998 | BETAMETHASONE DIPROPIONATE + FLUOCINONIDE, GENTAMICIN & SALICYLIC ACID oint |
| 92944990 | TOBRAMYCIN inj 80mg/2ml |
| 92945990 | TOBRAMYCIN inj 40mg/1ml |
| 93155990 | TOBRAMYCIN inj 80mg/2ml |
| 93156990 | TOBRAMYCIN inj 40mg/1ml |
| 93157990 | TOBRAMYCIN inj 240mg/6ml |
| 93158990 | TOBRAMYCIN inj 80mg/2ml |
| 93159990 | TOBRAMYCIN inj 40mg/1ml |
| 93205996 | TOBRAMYCIN neb soln 300mg/5ml |
| 93205997 | TOBRAMYCIN inj 40mg/ml |
| 93205998 | TOBRAMYCIN inj 10mg/ml |
| 93297990 | GENTAMICIN inj 80mg/2ml |
| 93298990 | GENTAMICIN inj 40mg/1ml |
| 94113992 | CIDOMYCIN 160 MG INJ |
| 94206992 | KANNASYN soln 1 GM INJ |
| 94242992 | MYCIFRADIN STERILE pwdr 350 MG POW |
| 94465992 | GARAMYCIN 40 MG INJ |
| 94481992 | KANTREX 1 GM INJ |
| 94482992 | KANAMYCIN 250 MG INJ |
| 94508992 | MYCIFRADIN .5 GM TAB |
| 94808990 | GENTAMICIN intrathec inj 5mg/1ml |
| 94977990 | GENTAMICIN inj 20mg/2ml |
| 95037990 | GENTAMICIN inj 40mg/ml |
| 95055992 | GENTICIN 25 MG INJ |
| 95059997 | AMIKACIN SULPHATE inj 500mg/2ml |
| 95059998 | AMIKACIN SULPHATE inj 100mg/2ml |
| 95060997 | AMIKACIN SULPHATE inj 500mg/2ml |
| 95060998 | AMIKACIN SULPHATE inj 100mg/2ml |
| 95081992 | GUANIMYCIN SUS |
| 95154997 | TOBRAMYCIN inj 40mg/ml |
| 95154998 | TOBRAMYCIN inj 10mg/ml |
| 95183992 | KANTREX 250 MG CAP |
| 95295992 | MYCIGUENT 500 MG TAB |
| 95697990 | TOBRAMYCIN inj 40mg/ml |
| 95731996 | NETILMICIN inj 100mg/ml |
| 95731997 | NETILMICIN inj 50mg/1ml |
| 95731998 | NETILMICIN inj 15mg/1.5ml |
| 95732996 | NETILMICIN inj 100mg/ml |
| 95732997 | NETILMICIN inj 50mg/1ml |
| 95732998 | NETILMICIN inj 15mg/1.5ml |
| 95737998 | NEOMYCIN SULPHATE tabs 500mg |
| 95748997 | NEOMYCIN SULPHATE elixir 100mg/5ml |
| 95748998 | NEOMYCIN SULPHATE tabs 500mg |
| 96044997 | KANAMYCIN soln 250mg/ml |
| 96044998 | KANAMYCIN pwdr 1g/vial |
| 96080992 | NETILLIN 150 MG INJ |
| 96199992 | DIHYDROSTREPTOMYCIN SULPH/KAOLIN LIGHT/S .25 GM SUS |
| 96258992 | GARAMYCIN PAED 10 MG INJ |
| 96261992 | GARAMYCIN 80 MG INJ |
| 96285997 | GENTAMICIN sterile pwdr 1g/vial |
| 96285998 | GENTAMICIN intrathec inj 1mg/ml |
| 96287998 | GENTAMICIN inj 80mg/ml |
| 96288998 | GENTAMICIN inj 40mg/ml |
| 96292998 | GENTAMICIN sterile pwdr 1g/vial |
| 96293996 | GENTAMICIN intrathec inj 5mg/1ml |
| 96293997 | GENTAMICIN intrathec inj 1mg/ml |
| 96293998 | GENTAMICIN inj 80mg/ml |
| 96294996 | GENTAMICIN inj 60mg/ml |
| 96294997 | GENTAMICIN inj 40mg/ml |
| 96294998 | GENTAMICIN inj 20mg/2ml |
| 96306992 | KANAMYCIN 333 MG INJ |
| 96354992 | NEOMYCIN SULPHATE 500 MG SYR |
| 96355992 | NEOMYCIN 500 MG CAP |
| 96356992 | NETILMICIN SULPHATE 150 MG INJ |
| 96479997 | FRAMYCETIN pwdr 500mg/vial |
| 96479998 | FRAMYCETIN tabs 250mg |
| 96485997 | FRAMYCETIN pwdr 500mg/vial |
| 96487998 | FRAMYCETIN tabs 250mg |
| 96671992 | GENTAMYCIN 40 MG INJ |
| 96672992 | GENTAMICIN 160 MG INJ |
| 96738990 | TOBRAMYCIN inj 40mg/ml |
| 96746990 | GENTAMICIN inj 40mg/ml |
| 97194990 | NEOMYCIN SULPHATE pwdr |
| 97262990 | AMIKACIN SULPHATE inj 500mg/2ml |
| 97335998 | GENTAMICIN inj 20mg/2ml |
| 97336998 | GENTAMICIN inj 80mg/2ml |
| 97518992 | GENTICIN 80 MG INJ |
| 97632992 | KANAMYCIN 250 MG CAP |
| 98283998 | GENTAMICIN inj 20mg/2ml |
| 98645998 | KANAMYCIN soln 250mg/ml |
| 99351997 | NEOMYCIN SULPHATE elixir 100mg/5ml |
| 99351998 | NEOMYCIN SULPHATE tabs 500mg |
| 99485988 | NEOMYCIN SULPHATE pwdr |
| 99485989 | NEOMYCIN SULPHATE tabs 500mg |
| 99520998 | KANAMYCIN pwdr 1g/vial |
| 99827996 | GENTAMICIN sterile pwdr 1g/vial |
| 99827997 | GENTAMICIN intrathec inj 5mg/1ml |
| 99827998 | GENTAMICIN inj 40mg/ml |
| 99850992 | AMIKIN paed 100 MG INJ |
| 99851992 | AMIKIN 500 MG INJ |
| 99934992 | KANAMYCIN 1 GM SYR |
| 93398992 | DAPSONE 5 MG TAB |
| 95218990 | DAPSONE tabs 100mg |
| 95219990 | DAPSONE tabs 50mg |
| 96175992 | DAPSONE 12.5 MG TAB |
| 96176992 | DAPSONE 25 MG TAB |
| 96643998 | CLOFAZIMINE caps 100mg |
| 97234992 | DAPSONE 200 MG TAB |
| 97235992 | DAPSONE 75 MG TAB |
| 97986997 | DAPSONE tabs 100mg |
| 97986998 | DAPSONE tabs 50mg |
| 98995989 | DAPSONE tabs 100mg |
| 98995990 | DAPSONE tabs 50mg |
| 99498998 | CLOFAZIMINE caps 100mg |
| 82519998 | PARA-AMINOSALICYLIC ACID grans 4g |
| 83454998 | RIFAMPICIN + ISONIAZID tabs 150mg + 100mg |
| 83455998 | RIFAMPICIN + ISONIAZID tabs 300mg + 150mg |
| 83822998 | RIFAMPICIN oral liq |
| 83823998 | RIFABUTIN oral liq |
| 84825998 | PYRAZINAMIDE oral liq |
| 84957998 | ISONIAZID tabs |
| 85006998 | ETHAMBUTOL oral liq |
| 85531998 | ISONIAZID oral liq |
| 86472998 | RIFAMPICIN caps 150mg |
| 86674998 | RIFAMPICIN caps 300mg |
| 86740998 | PYRAZINAMIDE tabs 500mg |
| 86741998 | PYRAZINAMIDE tabs 500mg |
| 92156990 | ISONIAZID tabs 100mg |
| 92157990 | ISONIAZID tabs 50mg |
| 92876990 | CAPREOMYCIN inj 1g/vial |
| 92877998 | RIFABUTIN caps 150mg |
| 92878998 | RIFABUTIN caps 150mg |
| 93305992 | ISONIAZID 150 MG TAB |
| 93406992 | PYRAZINAMIDE 50 MG TAB |
| 94148998 | CAPREOMYCIN inj 1g/vial |
| 94168997 | CYCLOSERINE caps 250mg |
| 94168998 | CYCLOSERINE caps 125mg |
| 95247998 | STREPTOMYCIN SULPHATE inj 1g/vial |
| 95311998 | RIFAMPICIN inf 300mg/vial |
| 95314997 | RIFAMPICIN + ISONIAZID tabs 300mg + 150mg |
| 95314998 | RIFAMPICIN + ISONIAZID tabs 150mg + 100mg |
| 95315998 | RIFAMPICIN + ISONIAZID & PYRAZINAMIDE tabs |
| 95316997 | RIFAMPICIN syrp 100mg/5ml |
| 95316998 | RIFAMPICIN caps 300mg |
| 95317996 | RIFAMPICIN caps 150mg |
| 95317997 | RIFAMPICIN pow conc sol inf 600mg |
| 95317998 | RIFAMPICIN inf 300mg/vial |
| 95364998 | PYRAZINAMIDE tabs 500mg |
| 95365998 | PYRAZINAMIDE + RIFAMPICIN & ISONIAZID tabs |
| 95366998 | PYRAZINAMIDE tabs 500mg |
| 95996992 | SOD AMINOSAL 1.6G/33.3MG ISONIAZID CAP |
| 96071992 | ETHIONAMIDE 125 MG TAB |
| 96093998 | RIFAMPICIN + ISONIAZID & PYRAZINAMIDE tabs |
| 96094998 | ISONIAZID + ETHAMBUTOL tabs 100mg + 365mg |
| 96095996 | ISONIAZID + ETHAMBUTOL tabs 100mg + 300mg |
| 96095997 | ISONIAZID + ETHAMBUTOL tabs 100mg + 250mg |
| 96095998 | ISONIAZID + ETHAMBUTOL tabs 100mg + 200mg |
| 96096998 | ISONIAZID + RIFAMPICIN & PYRAZINAMIDE tabs |
| 96097997 | ISONIAZID + RIFAMPICIN tabs 150mg + 300mg |
| 96097998 | ISONIAZID + RIFAMPICIN tabs 100mg + 150mg |
| 96098998 | ISONIAZID inj 50mg/2ml |
| 96133990 | CYCLOSERINE caps 250mg |
| 96300992 | ISONIAZID 130 MG ELI |
| 96301992 | ISONIAZID 60 MG SYR |
| 96302992 | ISONIAZID 10 MG ELI |
| 96303992 | ISONIAZID 500 MG ELI |
| 96304992 | ISONIAZID 50 MG SUS |
| 96305992 | ISONIAZID / PYRAZINAMIDE / RIFAMPICIN 50 MG TAB |
| 96420992 | PYRAZINAMIDE 375 MG CAP |
| 96443992 | SOD AMINOSAL 2G/50MG ISONIAZID PAED GM CAP |
| 96444992 | SOD AMINOSAL 1.5G/33MG ISONIAZID GM CAP |
| 96627992 | CYCLOSERINE 250 MG PUL |
| 96650992 | ETHAMBUTOL HCl 400 MG TAB |
| 96693992 | ISONIAZID 300 MG TAB |
| 96694992 | ISONIAZID 100 MG SYR |
| 96771998 | ETHAMBUTOL + ISONIAZID tabs 365mg + 100mg |
| 96772996 | ETHAMBUTOL + ISONIAZID tabs 300mg + 100mg |
| 96772997 | ETHAMBUTOL + ISONIAZID tabs 250mg + 100mg |
| 96772998 | ETHAMBUTOL + ISONIAZID tabs 200mg + 100mg |
| 96773996 | ETHAMBUTOL pwdr |
| 96773997 | ETHAMBUTOL tabs 400mg |
| 96773998 | ETHAMBUTOL tabs 100mg |
| 96785990 | ISONIAZID inj 50mg/2ml |
| 96786992 | TREVINTIX TAB |
| 96836989 | RIFAMPICIN caps 300mg |
| 96836990 | RIFAMPICIN caps 150mg |
| 96999989 | ISONIAZID tabs 100mg |
| 96999990 | ISONIAZID tabs 50mg |
| 97224992 | CYCLOSERINE 25 MG SUP |
| 97225992 | CYCLOSERINE 250 MG TAB |
| 97384992 | ETHAMBUTOL 250 MG SYR |
| 97385992 | ETHAMBUTOL SYR |
| 97386992 | ETHAMBUTOL 500 MG SYR |
| 97611992 | ISONIAZID 200 MG TAB |
| 97619992 | ISONIAZID 10 MG TAB |
| 97663989 | ETHAMBUTOL tabs 400mg |
| 97663990 | ETHAMBUTOL tabs 100mg |
| 97930989 | RIFAMPICIN caps 300mg |
| 97930990 | RIFAMPICIN caps 150mg |
| 97960992 | PROTHIONAMIDE 500 MG TAB |
| 97961992 | PROTHIONAMIDE 125 MG TAB |
| 97973990 | STREPTOMYCIN SULPHATE inj 1g/vial |
| 97984998 | ISONIAZID sf oral soln 50mg/5ml |
| 97985997 | ISONIAZID tabs 100mg |
| 97985998 | ISONIAZID tabs 50mg |
| 98040992 | SOD AMINOSAL 6G/150MG ISONIAZID 6 GM CAP |
| 98041992 | SOD AMINOSAL 1.5G/50MG ISONIAZID GM CAP |
| 98042992 | SOD AMINOSAL 1.5G/25MG ISONIAZID GM CAP |
| 98043992 | SODIUM AMINOSALICYLATE POW |
| 98137992 | STREPTOMYCIN 250 MG CAP |
| 98138992 | STREPTOMYCIN 500 MG CAP |
| 98168992 | THIACETAZONE TAB |
| 98198992 | TREVINTIX 500 MG TAB |
| 98339998 | ISONIAZID + ETHAMBUTOL tabs 100mg + 365mg |
| 98362989 | RIFAMPICIN caps 300mg |
| 98362990 | RIFAMPICIN caps 150mg |
| 98529998 | ISONIAZID inj 50mg/2ml |
| 98684997 | RIFAMPICIN + ISONIAZID tabs 300mg + 150mg |
| 98684998 | RIFAMPICIN + ISONIAZID tabs 150mg + 100mg |
| 98685990 | ISONIAZID sf oral soln 50mg/5ml |
| 98685998 | RIFAMPICIN pow conc sol inf 600mg |
| 98694998 | RIFAMPICIN + ISONIAZID tabs 300mg + 150mg |
| 98819997 | CYCLOSERINE caps 250mg |
| 98819998 | CYCLOSERINE caps 125mg |
| 99176990 | ISONIAZID tabs 100mg |
| 99177989 | ISONIAZID tabs 100mg |
| 99177990 | ISONIAZID tabs 50mg |
| 99178998 | RIFAMPICIN + ISONIAZID tabs 150mg + 100mg |
| 99179996 | RIFAMPICIN syrp 100mg/5ml |
| 99179997 | RIFAMPICIN caps 300mg |
| 99179998 | RIFAMPICIN caps 150mg |
| 99183996 | RIFAMPICIN syrp 100mg/5ml |
| 99183997 | RIFAMPICIN caps 300mg |
| 99183998 | RIFAMPICIN caps 150mg |
| 99386996 | ISONIAZID + ETHAMBUTOL tabs 100mg + 300mg |
| 99386997 | ISONIAZID + ETHAMBUTOL tabs 100mg + 250mg |
| 99386998 | ISONIAZID + ETHAMBUTOL tabs 100mg + 200mg |
| 99388996 | ETHAMBUTOL pwdr |
| 99388997 | ETHAMBUTOL tabs 400mg |
| 99388998 | ETHAMBUTOL tabs 100mg |
| 99547989 | ISONIAZID tabs 100mg |
| 99547990 | ISONIAZID tabs 50mg |
| 99854998 | CAPREOMYCIN inj 1g/vial |
| 99924992 | ISONIAZID 50 MG SYR |
| 99925992 | ISONIAZID 60 MG ELI |
| 99926992 | ISONIAZID 25 MG ELI |
| 99927992 | ISONIAZID 150 MG SYR |
| 99928992 | ISONIAZID 25 MG SYR |
| 99929992 | ISONIAZID 100 MG ELI |
| 99930992 | ISONIAZID 200 MG ELI |
| 99931992 | ISONIAZID 75 MG SYR |
| 99932992 | ISONIAZID 200 MG SYR |
| 99981992 | ISONIAZID ELI |
| 99982992 | ISONIAZID 150 MG ELI |
| 99983992 | ISONIAZID 500 MG SUS |
| 99984992 | ISONIAZID 150 MG SUS |
| 82074998 | AZTREONAM pdr for soln for neb 75mg |
| 82085998 | AZTREONAM pdr for soln for neb 75mg |
| 82660998 | CEFALEXIN oral susp 500mg/5ml |
| 82661998 | CEFALEXIN oral susp 250mg/5ml |
| 82662998 | CEFALEXIN oral susp 125mg/5ml |
| 82663998 | CEFALEXIN caps 500mg |
| 82664998 | CEFALEXIN caps 250mg |
| 82665998 | CEFALEXIN tabs 500mg |
| 82666998 | CEFALEXIN tabs 250mg |
| 83486998 | DORIPENEM pdr/inf.soln. 500mg/vial |
| 83487998 | DORIPENEM pdr/inf.soln. 500mg/vial |
| 83656998 | CEFUROXIME (AS SODIUM SALT) inj 1.5g/vial |
| 83657998 | CEFUROXIME (AS SODIUM SALT) inj 750mg/vial |
| 85280998 | CEFUROXIME (AS AXETIL) (IPU) tabs 500mg |
| 85939998 | CEFTAZIDIME pwdr/inj.soln 2g/vial |
| 85940998 | CEFTAZIDIME pwdr/inj.soln 1g/vial |
| 85943998 | CEFALEXIN oral susp 250mg/5ml |
| 85944998 | CEFALEXIN oral susp 125mg/5ml |
| 85945998 | CEFALEXIN caps 500mg |
| 85946998 | CEFALEXIN caps 250mg |
| 85947998 | CEFALEXIN tabs 500mg |
| 85948998 | CEFALEXIN tabs 250mg |
| 85949998 | CEFACLOR caps 500mg |
| 85950998 | CEFACLOR susp 250mg/5ml |
| 85951998 | CEFACLOR susp 125mg/5ml |
| 85952998 | CEFACLOR mr tab 375mg |
| 87657998 | CEFALEXIN sf oral susp 125mg/5ml |
| 87658998 | CEFALEXIN sf oral susp 250mg/5ml |
| 88222998 | CEFACLOR susp 250mg/5ml |
| 88245996 | CEFACLOR susp 125mg/5ml |
| 88245997 | CEFACLOR caps 500mg |
| 88245998 | CEFACLOR caps 250mg |
| 88392997 | CEFRADINE caps 500mg |
| 88392998 | CEFRADINE caps 250mg |
| 88818998 | CEFACLOR sf susp 250mg/5ml |
| 89041996 | CEFOTAXIME pwdr/inj.soln 500mg/vial |
| 89041997 | CEFOTAXIME pwdr/inj.soln 2g/vial |
| 89041998 | CEFOTAXIME pwdr/inj.soln 1g/vial |
| 89252996 | CEFPROZIL susp 250mg/5ml |
| 89252997 | CEFPROZIL tabs 500mg |
| 89252998 | CEFPROZIL tabs 250mg |
| 89452996 | CEFACLOR sf susp 125mg/5ml |
| 89452997 | CEFACLOR caps 500mg |
| 89452998 | CEFACLOR caps 250mg |
| 89653998 | ERTAPENEM pow conc sol inf 1g/vial |
| 89654998 | ERTAPENEM pow conc sol inf 1g/vial |
| 90028997 | CEFALEXIN oral susp 250mg/5ml |
| 90028998 | CEFALEXIN oral susp 125mg/5ml |
| 90029996 | CEFALEXIN tabs 500mg |
| 90029997 | CEFALEXIN caps 500mg |
| 90029998 | CEFALEXIN caps 250mg |
| 90961998 | CEFACLOR mr tab 375mg |
| 91252997 | CEFPIROME inj 2g |
| 91252998 | CEFPIROME inj 1g |
| 91253997 | CEFPIROME inj 2g |
| 91253998 | CEFPIROME inj 1g |
| 91365998 | IMIPENEM + CILASTATIN pwdr for inj.suspn. 500mg + 500mg |
| 91366998 | IMIPENEM + CILASTATIN pwdr for inj.suspn. 500mg + 500mg |
| 91439996 | MEROPENEM pwdr/inj.soln 1g/vial |
| 91439997 | MEROPENEM pwdr/inj.soln 500mg/vial |
| 91439998 | MEROPENEM inj 250mg |
| 91440996 | MEROPENEM pwdr/inj.soln 1g/vial |
| 91440997 | MEROPENEM pwdr/inj.soln 500mg/vial |
| 91440998 | MEROPENEM inj 250mg |
| 91561996 | CEFPROZIL susp 250mg/5ml |
| 91561997 | CEFPROZIL tabs 500mg |
| 91561998 | CEFPROZIL tabs 250mg |
| 92076996 | CEFALEXIN caps 250mg |
| 92076997 | CEFALEXIN tabs 500mg |
| 92076998 | CEFALEXIN tabs 250mg |
| 92077996 | CEFALEXIN oral susp 250mg/5ml |
| 92077997 | CEFALEXIN oral susp 125mg/5ml |
| 92077998 | CEFALEXIN caps 500mg |
| 92432990 | CEFTAZIDIME pwdr/inj.soln 2g/vial |
| 92520998 | CEFTAZIDIME pdr/inf.soln. 2g/vial |
| 92531996 | CEFTAZIDIME pwdr/inj.soln 2g/vial |
| 92531997 | CEFTAZIDIME pwdr/inj.soln 1g/vial |
| 92531998 | CEFTAZIDIME pwdr/inj.soln 500mg/vial |
| 92634996 | CEFTIBUTEN susp 180mg/5ml |
| 92634997 | CEFTIBUTEN susp 90mg/5ml |
| 92634998 | CEFTIBUTEN caps 400mg |
| 92635996 | CEFTIBUTEN susp 180mg/5ml |
| 92635997 | CEFTIBUTEN susp 90mg/5ml |
| 92635998 | CEFTIBUTEN caps 400mg |
| 92999992 | FORTUM MONOVIAL 2G |
| 93030998 | CEFUROXIME & SALINE inf 750mg |
| 93031998 | CEFUROXIME + METRONIDAZOLE inf 750mg + 500mg |
| 93032998 | CEFTAZIDIME & SALINE inf 2g |
| 93033998 | CEFTAZIDIME & SALINE inf 2g |
| 93034998 | CEFUROXIME & SALINE inf 750mg |
| 93035998 | CEFUROXIME + METRONIDAZOLE inf 750mg + 500mg |
| 93101990 | CEFALEXIN tabs 500mg |
| 93102990 | CEFALEXIN tabs 250mg |
| 93103990 | CEFALEXIN oral susp 250mg/5ml |
| 93104990 | CEFALEXIN oral susp 125mg/5ml |
| 93193998 | CEFUROXIME (AS SODIUM SALT) inj 1.5g/vial |
| 93204990 | CEFUROXIME (AS AXETIL) tabs 250mg |
| 93234990 | CEFUROXIME (AS AXETIL) tabs 250mg |
| 93245997 | CEFIXIME susp 100mg/5ml |
| 93245998 | CEFIXIME tabs 200mg |
| 93246997 | CEFIXIME susp 100mg/5ml |
| 93246998 | CEFIXIME tabs 200mg |
| 93393992 | CEPHRADINE 250 MG INJ |
| 93529997 | CEFALEXIN caps 500mg |
| 93529998 | CEFALEXIN caps 250mg |
| 93533998 | CEFALEXIN tabs 1g |
| 93534998 | CEFALEXIN tabs 1g |
| 93612990 | CEFOTAXIME pwdr/inj.soln 1g/vial |
| 93613990 | CEFOTAXIME pwdr/inj.soln 500mg/vial |
| 93750990 | CEFUROXIME (AS SODIUM SALT) inj 1.5g/vial |
| 93751990 | CEFUROXIME (AS SODIUM SALT) inj 750mg/vial |
| 93776996 | CEFTRIAXONE inj 2g |
| 93776997 | CEFTRIAXONE inj 1g |
| 93776998 | CEFTRIAXONE inj 250mg |
| 93803990 | CEFTAZIDIME pwdr/inj.soln 2g/vial |
| 93804990 | CEFTAZIDIME pwdr/inj.soln 1g/vial |
| 93858990 | CEFUROXIME (AS AXETIL) tabs 250mg |
| 93901997 | CEFACLOR mr tab 375mg |
| 93901998 | CEFACLOR mr tab 500mg |
| 94173990 | CEFALEXIN caps 500mg |
| 94174990 | CEFALEXIN caps 250mg |
| 94208992 | KEFLEX-C 125 MG TAB |
| 94212992 | LATAMOXEF SODIUM 500 MG INJ |
| 94300990 | CEFACLOR sf susp 250mg/5ml |
| 94301990 | CEFACLOR sf susp 125mg/5ml |
| 94373990 | CEFADROXIL caps 500mg |
| 94484992 | KEFLEX-C 250 MG TAB |
| 94528990 | CEFADROXIL caps 500mg |
| 94565992 | CEFLACOR MR 375 MG TAB |
| 94567990 | CEFTAZIDIME pwdr/inj.soln 2g/vial |
| 94568990 | CEFTAZIDIME pwdr/inj.soln 1g/vial |
| 94569990 | CEFTAZIDIME pwdr/inj.soln 500mg/vial |
| 94622990 | CEFALEXIN tabs 500mg |
| 94639997 | IMIPENEM + CILASTATIN pdr/inf.soln. 500mg + 500mg |
| 94639998 | IMIPENEM + CILASTATIN inj 250mg + 250mg |
| 94640997 | IMIPENEM + CILASTATIN pdr/inf.soln. 500mg + 500mg |
| 94640998 | IMIPENEM + CILASTATIN inj 250mg + 250mg |
| 94641990 | CEFALEXIN tabs 500mg |
| 94642990 | CEFALEXIN tabs 250mg |
| 94643990 | CEFALEXIN oral susp 250mg/5ml |
| 94644990 | CEFALEXIN oral susp 125mg/5ml |
| 94645990 | CEFALEXIN caps 500mg |
| 94646990 | CEFALEXIN caps 250mg |
| 94647990 | CEFACLOR caps 500mg |
| 94648990 | CEFACLOR caps 250mg |
| 94667996 | CEFAMANDOLE inj 2g/vial |
| 94667997 | CEFAMANDOLE inj 1g/vial |
| 94667998 | CEFAMANDOLE inj 500mg/vial |
| 94728998 | CEFALEXIN chewable tab 250mg |
| 94729996 | CEFALEXIN oral susp 250mg/5ml |
| 94729997 | CEFALEXIN oral susp 125mg/5ml |
| 94729998 | CEFALEXIN caps 500mg |
| 94730996 | CEFALEXIN caps 250mg |
| 94730997 | CEFALEXIN tabs 500mg |
| 94730998 | CEFALEXIN tabs 250mg |
| 94753990 | CEFUROXIME + METRONIDAZOLE inf 750mg + 500mg |
| 94766990 | CEFADROXIL caps 500mg |
| 94791997 | CEFACLOR mr tab 500mg |
| 94791998 | CEFACLOR mr tab 375mg |
| 94870998 | CEFACLOR caps 500mg |
| 94873996 | CEFACLOR sf susp 250mg/5ml |
| 94873997 | CEFACLOR sf susp 125mg/5ml |
| 94873998 | CEFACLOR caps 500mg |
| 94904996 | CEFUROXIME (AS AXETIL) susp 125mg/5ml |
| 94904997 | CEFUROXIME (AS AXETIL) tabs 250mg |
| 94904998 | CEFUROXIME (AS AXETIL) tabs 125mg |
| 94915996 | CEFUROXIME (AS AXETIL) susp 125mg/5ml |
| 94915997 | CEFUROXIME (AS AXETIL) tabs 250mg |
| 94915998 | CEFUROXIME (AS AXETIL) tabs 125mg |
| 95203992 | LATAMOXEF SODIUM 2 GM INJ |
| 95621990 | CEFRADINE caps 500mg |
| 95622990 | CEFRADINE caps 250mg |
| 95723992 | VELOSEF 125 MG SYR |
| 95758990 | CEFTRIAXONE inj 1g |
| 95798990 | CEFRADINE caps 500mg |
| 95799990 | CEFRADINE caps 250mg |
| 95815990 | CEFRADINE caps 500mg |
| 95816990 | CEFRADINE caps 250mg |
| 96027996 | LATAMOXEF DISODIUM inj 2g/vial |
| 96027997 | LATAMOXEF DISODIUM inj 1g/vial |
| 96027998 | LATAMOXEF DISODIUM inj 500mg/vial |
| 96028996 | LATAMOXEF DISODIUM inj 2g/vial |
| 96028997 | LATAMOXEF DISODIUM inj 1g/vial |
| 96028998 | LATAMOXEF DISODIUM inj 500mg/vial |
| 96039990 | CEFTRIAXONE inj 2g |
| 96040990 | CEFTRIAXONE inj 1g |
| 96146992 | CEPHALEXIN 125 MG TAB |
| 96147992 | CEPHRADINE 125 MG SYR |
| 96148992 | CEFAMANDOLE 2 GM INJ |
| 96149992 | CEPHALORIDINE 250 MG INJ |
| 96213990 | CEFOTAXIME pwdr/inj.soln 500mg/vial |
| 96214990 | CEFOTAXIME pwdr/inj.soln 2g/vial |
| 96215990 | CEFACLOR susp 250mg/5ml |
| 96223990 | CEFOTAXIME pwdr/inj.soln 1g/vial |
| 96224990 | CEFACLOR susp 125mg/5ml |
| 96239989 | CEFTRIAXONE inj 2g |
| 96239990 | CEFTRIAXONE inj 1g |
| 96275990 | CEFALEXIN tabs 250mg |
| 96277990 | CEFALEXIN oral susp 250mg/5ml |
| 96293988 | CEFOTAXIME pwdr/inj.soln 2g/vial |
| 96293989 | CEFOTAXIME pwdr/inj.soln 1g/vial |
| 96293990 | CEFOTAXIME pwdr/inj.soln 500mg/vial |
| 96431989 | CEFRADINE caps 500mg |
| 96431990 | CEFRADINE caps 250mg |
| 96433988 | CEFALEXIN caps 500mg |
| 96433989 | CEFALEXIN caps 250mg |
| 96433990 | CEFALEXIN oral susp 250mg/5ml |
| 96435988 | CEFALEXIN oral susp 125mg/5ml |
| 96435989 | CEFALEXIN tabs 500mg |
| 96435990 | CEFALEXIN tabs 250mg |
| 96639990 | CEFACLOR mr tab 375mg |
| 96649988 | CEFUROXIME (AS SODIUM SALT) inj 1.5g/vial |
| 96649989 | CEFUROXIME (AS SODIUM SALT) inj 750mg/vial |
| 96649990 | CEFUROXIME (AS SODIUM SALT) inj 250mg/vial |
| 96688989 | CEFRADINE caps 500mg |
| 96688990 | CEFRADINE caps 250mg |
| 96714992 | MONASPOR I/M & LIGNOCAINE 0.5% 1 GM INJ |
| 96833992 | CEFTAZIDIME INTERMATE inf DEVICE |
| 96834998 | CEFALEXIN paed drops 125mg/1.25ml |
| 96835997 | CEFALEXIN susp 250mg/5ml |
| 96835998 | CEFALEXIN susp 125mg/5ml |
| 96836996 | CEFALEXIN oral susp 500mg/5ml |
| 96836997 | CEFALEXIN oral susp 250mg/5ml |
| 96836998 | CEFALEXIN oral susp 125mg/5ml |
| 96837996 | CEFALEXIN chewable tab 250mg |
| 96837997 | CEFALEXIN tabs 500mg |
| 96837998 | CEFALEXIN tabs 250mg |
| 96838996 | CEFUROXIME (AS SODIUM SALT) inf 1.5g/vial |
| 96838997 | CEFUROXIME (AS SODIUM SALT) inj 750mg/vial |
| 96838998 | CEFUROXIME (AS SODIUM SALT) inj 250mg/vial |
| 96839996 | CEFTIZOXIME IV inj 2000mg/vial |
| 96839997 | CEFTIZOXIME IV inj 1000mg/vial |
| 96839998 | CEFTIZOXIME IV inj 500mg/vial |
| 96840996 | CEFTIZOXIME inj 2000mg/vial |
| 96840997 | CEFTIZOXIME inj 1000mg/vial |
| 96840998 | CEFTIZOXIME inj 500mg/vial |
| 96841996 | CEFTAZIDIME pwdr/inj.soln 3g/vial |
| 96841997 | CEFTAZIDIME pwdr/inj.soln 2g/vial |
| 96841998 | CEFTAZIDIME pdr/inf.soln. 2g/vial |
| 96842996 | CEFTAZIDIME pwdr/inj.soln 1g/vial |
| 96842997 | CEFTAZIDIME pwdr/inj.soln 500mg/vial |
| 96842998 | CEFTAZIDIME pwdr/inj.soln 250mg/vial |
| 96843997 | CEFSULODIN sterile pwdr 1g/vial |
| 96843998 | CEFSULODIN sterile pwdr 500mg/vial |
| 96844997 | CEFSULODIN sterile pwdr 1g/vial |
| 96844998 | CEFSULODIN sterile pwdr 500mg/vial |
| 96845997 | CEFRADINE inj 1000mg/vial |
| 96845998 | CEFRADINE inj 500mg/vial |
| 96846996 | CEFRADINE syrp 250mg/5ml |
| 96846997 | CEFRADINE caps 500mg |
| 96846998 | CEFRADINE caps 250mg |
| 96847997 | CEFOXITIN inj 2g/vial |
| 96847998 | CEFOXITIN inj 1g/vial |
| 96848997 | CEFOXITIN inj 2g/vial |
| 96848998 | CEFOXITIN inj 1g/vial |
| 96849996 | CEFOTAXIME pwdr/inj.soln 2g/vial |
| 96849997 | CEFOTAXIME pwdr/inj.soln 1g/vial |
| 96849998 | CEFOTAXIME pwdr/inj.soln 500mg/vial |
| 96850997 | CEFAZOLIN inj 1g/vial |
| 96850998 | CEFAZOLIN inj 500mg/vial |
| 96851998 | CEFALOTIN inj 1g |
| 96852997 | CEFALOTIN inj 1g |
| 96852998 | CEFALOTIN inj 1g/vial |
| 96853996 | CEFALORIDINE inj 1g |
| 96853997 | CEFALORIDINE inj 500mg |
| 96853998 | CEFALORIDINE inj 250mg |
| 96854996 | CEFALORIDINE inj 1g |
| 96854997 | CEFALORIDINE inj 500mg |
| 96854998 | CEFALORIDINE inj 250mg |
| 96855996 | CEFADROXIL susp 250mg/5ml |
| 96855997 | CEFADROXIL susp 125mg/5ml |
| 96855998 | CEFADROXIL caps 500mg |
| 96856996 | CEFACLOR susp 250mg/5ml |
| 96856997 | CEFACLOR susp 125mg/5ml |
| 96856998 | CEFACLOR caps 250mg |
| 96909988 | CEFALEXIN oral susp 250mg/5ml |
| 96909989 | CEFALEXIN oral susp 125mg/5ml |
| 96909990 | CEFALEXIN caps 500mg |
| 96911988 | CEFALEXIN caps 250mg |
| 96911989 | CEFALEXIN tabs 500mg |
| 96911990 | CEFALEXIN tabs 250mg |
| 96923989 | CEFACLOR mr tab 375mg |
| 96923990 | CEFACLOR sf susp 250mg/5ml |
| 96925988 | CEFACLOR sf susp 125mg/5ml |
| 96925989 | CEFACLOR caps 500mg |
| 96925990 | CEFACLOR caps 250mg |
| 96926998 | AZTREONAM inf 2g/vial |
| 96927996 | AZTREONAM pwdr/inj.soln 2g/vial |
| 96927997 | AZTREONAM pwdr/inj.soln 1g/vial |
| 96927998 | AZTREONAM pwdr/inj.soln 500mg/vial |
| 96928998 | AZTREONAM inf 2g/vial |
| 96929996 | AZTREONAM pwdr/inj.soln 2g/vial |
| 96929997 | AZTREONAM pwdr/inj.soln 1g/vial |
| 96929998 | AZTREONAM pwdr/inj.soln 500mg/vial |
| 97024989 | CEFRADINE caps 500mg |
| 97024990 | CEFRADINE caps 250mg |
| 97035988 | CEFACLOR caps 250mg |
| 97035989 | CEFACLOR sf susp 125mg/5ml |
| 97035990 | CEFACLOR sf susp 250mg/5ml |
| 97038990 | CEFACLOR caps 500mg |
| 97097992 | CEFTIZOXIME SODIUM 2 GM INJ |
| 97098992 | CEFTIZOXIME SODIUM 1 GM INJ |
| 97099992 | CEFTIZOXIME SODIUM .5 GM INJ |
| 97100992 | CEFIZOX 1 GM INJ |
| 97102992 | CEFSULODIN SODIUM & LIGNOCAINE 0.5% 1 GM INJ |
| 97103992 | CEFAMANDOLE 1 GM INJ |
| 97104992 | CEFAMANDOLE 500 MG INJ |
| 97105992 | CEPHALORIDINE 1 GM INJ |
| 97106992 | CEPHALORIDINE 500 MG INJ |
| 97171988 | CEFALEXIN oral susp 125mg/5ml |
| 97171989 | CEFALEXIN caps 500mg |
| 97171990 | CEFALEXIN caps 250mg |
| 97237989 | CEFALEXIN sf oral susp 250mg/5ml |
| 97237990 | CEFALEXIN sf oral susp 125mg/5ml |
| 97310998 | CEFADROXIL susp 500mg/5ml |
| 97311996 | CEFADROXIL susp 250mg/5ml |
| 97311997 | CEFADROXIL susp 125mg/5ml |
| 97311998 | CEFADROXIL caps 500mg |
| 97328997 | CEFACLOR susp 250mg/5ml |
| 97328998 | CEFACLOR susp 125mg/5ml |
| 97347996 | CEFAMANDOLE inj 2g/vial |
| 97347997 | CEFAMANDOLE inj 1g/vial |
| 97347998 | CEFAMANDOLE inj 500mg/vial |
| 97348997 | CEFAZOLIN inj 1g/vial |
| 97348998 | CEFAZOLIN inj 500mg/vial |
| 97349998 | CEFALOTIN inj 1g/vial |
| 97535990 | CEFACLOR susp 250mg/5ml |
| 97542990 | CEFACLOR sf susp 250mg/5ml |
| 97612998 | CEFUROXIME (AS SODIUM SALT) inf 1.5g/vial |
| 97613996 | CEFUROXIME (AS SODIUM SALT) inj 1.5g/vial |
| 97613997 | CEFUROXIME (AS SODIUM SALT) inj 750mg/vial |
| 97613998 | CEFUROXIME (AS SODIUM SALT) inj 250mg/vial |
| 97619996 | CEFTAZIDIME pwdr/inj.soln 3g/vial |
| 97619997 | CEFTAZIDIME pwdr/inj.soln 2g/vial |
| 97619998 | CEFTAZIDIME pdr/inf.soln. 2g/vial |
| 97620996 | CEFTAZIDIME pwdr/inj.soln 1g/vial |
| 97620997 | CEFTAZIDIME pwdr/inj.soln 500mg/vial |
| 97620998 | CEFTAZIDIME pwdr/inj.soln 250mg/vial |
| 97635992 | KEFLEX 500 MG INJ |
| 97649992 | LATAMOXEF SODIUM 1 GM INJ |
| 97650996 | CEFTRIAXONE inj 2g |
| 97650997 | CEFTRIAXONE inj 1g |
| 97650998 | CEFTRIAXONE inj 250mg |
| 97694988 | CEFACLOR susp 125mg/5ml |
| 97694989 | CEFACLOR caps 500mg |
| 97694990 | CEFACLOR caps 250mg |
| 97696988 | CEFACLOR sf susp 125mg/5ml |
| 97696989 | CEFACLOR caps 500mg |
| 97696990 | CEFACLOR caps 250mg |
| 97778988 | CEFALEXIN oral susp 250mg/5ml |
| 97778989 | CEFALEXIN oral susp 125mg/5ml |
| 97778990 | CEFALEXIN tabs 500mg |
| 97806997 | CEFPODOXIME susp 40mg/5ml |
| 97806998 | CEFPODOXIME tabs 100mg |
| 97839990 | CEFACLOR susp 250mg/5ml |
| 97840988 | CEFACLOR susp 125mg/5ml |
| 97840989 | CEFACLOR caps 500mg |
| 97840990 | CEFACLOR caps 250mg |
| 97859988 | CEFALEXIN caps 500mg |
| 97859989 | CEFALEXIN caps 250mg |
| 97859990 | CEFALEXIN tabs 250mg |
| 97885998 | CEFALEXIN paed drops 125mg/1.25ml |
| 97886996 | CEFALEXIN oral susp 250mg/5ml |
| 97886997 | CEFALEXIN oral susp 125mg/5ml |
| 97886998 | CEFALEXIN oral susp 500mg/5ml |
| 97887996 | CEFALEXIN oral susp 250mg/5ml |
| 97887997 | CEFALEXIN oral susp 125mg/5ml |
| 97887998 | CEFALEXIN caps 500mg |
| 97888996 | CEFALEXIN caps 250mg |
| 97888997 | CEFALEXIN tabs 500mg |
| 97888998 | CEFALEXIN tabs 250mg |
| 97950992 | PRIMAXIN INJ |
| 97990988 | CEFALEXIN oral susp 250mg/5ml |
| 97990989 | CEFALEXIN oral susp 125mg/5ml |
| 97990990 | CEFALEXIN caps 500mg |
| 97991988 | CEFALEXIN caps 250mg |
| 97991989 | CEFALEXIN tabs 500mg |
| 97991990 | CEFALEXIN tabs 250mg |
| 98010990 | CEFALEXIN tabs 250mg |
| 98015989 | CEFALEXIN caps 250mg |
| 98015990 | CEFALEXIN tabs 250mg |
| 98141990 | CEFALEXIN tabs 250mg |
| 98195988 | CEFALEXIN oral susp 250mg/5ml |
| 98195989 | CEFALEXIN caps 500mg |
| 98195990 | CEFALEXIN caps 250mg |
| 98479998 | CEFODIZIME inj 1g/vial |
| 98494998 | CEFODIZIME inj 1g/vial |
| 98634996 | CEFTIZOXIME inj 2000mg/vial |
| 98634997 | CEFTIZOXIME inj 1000mg/vial |
| 98634998 | CEFTIZOXIME inj 500mg/vial |
| 98635988 | CEFALEXIN oral susp 250mg/5ml |
| 98635989 | CEFALEXIN oral susp 125mg/5ml |
| 98635990 | CEFALEXIN tabs 500mg |
| 98635996 | CEFTIZOXIME inj 2000mg/vial |
| 98635997 | CEFTIZOXIME inj 1000mg/vial |
| 98635998 | CEFTIZOXIME inj 500mg/vial |
| 98638988 | CEFALEXIN tabs 250mg |
| 98638989 | CEFALEXIN caps 500mg |
| 98638990 | CEFALEXIN caps 250mg |
| 98813998 | CEFADROXIL susp 500mg/5ml |
| 98940997 | CEFPODOXIME susp 40mg/5ml |
| 98940998 | CEFPODOXIME tabs 100mg |
| 98983997 | CEFRADINE inj 1000mg/vial |
| 98983998 | CEFRADINE inj 500mg/vial |
| 98984996 | CEFRADINE syrp 250mg/5ml |
| 98984997 | CEFRADINE caps 500mg |
| 98984998 | CEFRADINE caps 250mg |
| 99255990 | CEFALEXIN caps 500mg |
| 99698988 | CEFALEXIN oral susp 250mg/5ml |
| 99698989 | CEFALEXIN oral susp 125mg/5ml |
| 99698990 | CEFALEXIN tabs 500mg |
| 99699988 | CEFALEXIN tabs 250mg |
| 99699989 | CEFALEXIN caps 500mg |
| 99699990 | CEFALEXIN caps 250mg |
| 99700988 | CEFALEXIN tabs 500mg |
| 99700989 | CEFALEXIN caps 500mg |
| 99700990 | CEFALEXIN caps 250mg |
| 99701988 | CEFALEXIN caps 250mg |
| 99701989 | CEFALEXIN tabs 500mg |
| 99701990 | CEFALEXIN tabs 250mg |
| 99741996 | CEFACLOR susp 250mg/5ml |
| 99741997 | CEFACLOR susp 125mg/5ml |
| 99741998 | CEFACLOR caps 250mg |
| 99825996 | CEFOTAXIME pwdr/inj.soln 2g/vial |
| 99825997 | CEFOTAXIME pwdr/inj.soln 1g/vial |
| 99825998 | CEFOTAXIME pwdr/inj.soln 500mg/vial |
| 85035998 | CLINDAMYCIN oral liq |
| 85338998 | CLINDAMYCIN PHOSPHATE inj 600mg/4ml |
| 85339998 | CLINDAMYCIN PHOSPHATE inj 300mg/2ml |
| 85340998 | CLINDAMYCIN PHOSPHATE inj 600mg/4ml |
| 85341998 | CLINDAMYCIN PHOSPHATE inj 300mg/2ml |
| 88708998 | CLINDAMYCIN PHOSPHATE inj 150mg/ml |
| 95331990 | CLINDAMYCIN HCl caps 150mg |
| 95558990 | CLINDAMYCIN HCl caps 150mg |
| 96009996 | LINCOMYCIN HCl inj 300mg/ml |
| 96009997 | LINCOMYCIN HCl syrp 250mg/5ml |
| 96009998 | LINCOMYCIN HCl caps 500mg |
| 96010996 | LINCOMYCIN HCl inj 300mg/ml |
| 96010997 | LINCOMYCIN HCl syrp 250mg/5ml |
| 96010998 | LINCOMYCIN HCl caps 500mg |
| 96654998 | CLINDAMYCIN HCl inj 150mg/ml |
| 96655996 | CLINDAMYCIN HCl grans for susp 75mg/5ml |
| 96655997 | CLINDAMYCIN HCl caps 150mg |
| 96655998 | CLINDAMYCIN HCl caps 75mg |
| 97924998 | CLINDAMYCIN PHOSPHATE inj 150mg/ml |
| 97925996 | CLINDAMYCIN HCl grans for susp 75mg/5ml |
| 97925997 | CLINDAMYCIN HCl caps 150mg |
| 97925998 | CLINDAMYCIN HCl caps 75mg |
| 83061998 | ERYTHROMYCIN ETHYLSUCCINATE sf susp 125mg/5ml |
| 83062998 | ERYTHROMYCIN ETHYLSUCCINATE sf susp 500mg/5ml |
| 83063998 | ERYTHROMYCIN ETHYLSUCCINATE sf susp 250mg/5ml |
| 83064998 | ERYTHROMYCIN ETHYLSUCCINATE tabs 500mg |
| 83065998 | ERYTHROMYCIN STEARATE tabs 500mg |
| 83066998 | ERYTHROMYCIN STEARATE tabs 250mg |
| 83472998 | CLARITHROMYCIN paed susp 250mg/5ml |
| 83474998 | CLARITHROMYCIN paed susp 125mg/5ml |
| 83543998 | AZITHROMYCIN tabs 500mg |
| 85194998 | ERYTHROMYCIN LACTOBIONATE pow conc sol inf 1g |
| 85408998 | AZITHROMYCIN tabs 250mg |
| 86473998 | CLARITHROMYCIN oral susp granules 250mg/straw |
| 86474998 | CLARITHROMYCIN oral susp granules 187.5mg/straw |
| 86475998 | CLARITHROMYCIN oral susp granules 125mg/straw |
| 86476998 | CLARITHROMYCIN oral susp granules 250mg/straw |
| 86477998 | CLARITHROMYCIN oral susp granules 187.5mg/straw |
| 86478998 | CLARITHROMYCIN oral susp granules 125mg/straw |
| 87506998 | ERYTHROMYCIN caps(ec grans) 250mg |
| 88231997 | ERYTHROMYCIN ETHYLSUCCINATE susp 250mg/5ml |
| 88231998 | ERYTHROMYCIN ETHYLSUCCINATE susp 125mg/5ml |
| 88378997 | CLARITHROMYCIN paed susp 250mg/5ml |
| 88378998 | CLARITHROMYCIN grans for susp 250mg/sachet |
| 88961998 | SPIRAMYCIN tabs 500mg |
| 88962998 | SPIRAMYCIN tabs 500mg |
| 89246998 | CLARITHROMYCIN mr tab 500mg |
| 89421998 | ERYTHROMYCIN ETHYLSUCCINATE (COATED) sf oral susp 250mg/5ml |
| 89423998 | ERYTHROMYCIN ETHYLSUCCINATE (COATED) sf oral susp 250mg/5ml |
| 89630996 | ERYTHROMYCIN ETHYLSUCCINATE susp 500mg/5ml |
| 89630997 | ERYTHROMYCIN ETHYLSUCCINATE susp 250mg/5ml |
| 89630998 | ERYTHROMYCIN ETHYLSUCCINATE susp 125mg/5ml |
| 90499996 | ERYTHROMYCIN ETHYLSUCCINATE sf susp 500mg/5ml |
| 90499997 | ERYTHROMYCIN ETHYLSUCCINATE sf susp 250mg/5ml |
| 90499998 | ERYTHROMYCIN ETHYLSUCCINATE sf susp 125mg/5ml |
| 90567998 | ERYTHROMYCIN ETHYLSUCCINATE sf susp 500mg/5ml |
| 90844998 | ERYTHROMYCIN caps(ec grans) 250mg |
| 92104998 | ERYTHROMYCIN caps(ec grans) 250mg |
| 92247990 | AZITHROMYCIN tabs 250mg |
| 92247998 | TELITHROMYCIN tabs 400mg |
| 92248990 | AZITHROMYCIN tabs 500mg |
| 92248998 | TELITHROMYCIN tabs 400mg |
| 92495996 | CLARITHROMYCIN paed susp 250mg/5ml |
| 92495997 | CLARITHROMYCIN grans for susp 250mg/sachet |
| 92495998 | CLARITHROMYCIN mr tab 500mg |
| 92595990 | AZITHROMYCIN caps 250mg |
| 92605990 | AZITHROMYCIN caps 250mg |
| 92631990 | CLARITHROMYCIN tabs 500mg |
| 92632990 | CLARITHROMYCIN tabs 250mg |
| 92661990 | AZITHROMYCIN susp 40mg/ml |
| 92763990 | ERYTHROMYCIN ETHYLSUCCINATE sf susp 500mg/5ml |
| 92764990 | ERYTHROMYCIN ETHYLSUCCINATE sf susp 250mg/5ml |
| 92765990 | ERYTHROMYCIN ETHYLSUCCINATE sf susp 125mg/5ml |
| 92798990 | AZITHROMYCIN susp 40mg/ml |
| 92853990 | CLARITHROMYCIN pow conc sol inf 500mg/vial |
| 92883998 | CLARITHROMYCIN pow conc sol inf 500mg/vial |
| 92884998 | CLARITHROMYCIN pow conc sol inf 500mg/vial |
| 92997990 | AZITHROMYCIN tabs 500mg |
| 93003992 | ERYMIN 250 MG/5ML SUS |
| 93147997 | ERYTHROMYCIN ETHYLSUCCINATE sach 250mg |
| 93147998 | ERYTHROMYCIN ETHYLSUCCINATE sach 125mg |
| 93171990 | AZITHROMYCIN tabs 250mg |
| 93177990 | AZITHROMYCIN tabs 500mg |
| 93316992 | ERYTHROMYCIN ETHYLSUCCINATE SF 125 MG/5ML SUS |
| 93317992 | ERYTHROMYCIN SF sach 250 MG |
| 93856990 | CLARITHROMYCIN paed susp 250mg/5ml |
| 93857990 | CLARITHROMYCIN paed susp 125mg/5ml |
| 93914990 | ERYTHROMYCIN ec tab 250mg |
| 94151990 | CLARITHROMYCIN paed susp 250mg/5ml |
| 94152990 | CLARITHROMYCIN paed susp 125mg/5ml |
| 94153992 | ERYCEN 250 MG SUS |
| 94154992 | ERMYSIN E/C 250 MG TAB |
| 94185990 | CLARITHROMYCIN tabs 250mg |
| 94215990 | ERYTHROMYCIN ETHYLSUCCINATE sf susp 500mg/5ml |
| 94216990 | ERYTHROMYCIN ETHYLSUCCINATE sf susp 250mg/5ml |
| 94217990 | ERYTHROMYCIN ETHYLSUCCINATE sf susp 125mg/5ml |
| 94218990 | ERYTHROMYCIN ETHYLSUCCINATE susp 500mg/5ml |
| 94219990 | ERYTHROMYCIN ETHYLSUCCINATE susp 250mg/5ml |
| 94220990 | ERYTHROMYCIN ETHYLSUCCINATE susp 125mg/5ml |
| 94358990 | ERYTHROMYCIN ec tab 250mg |
| 94370990 | CLARITHROMYCIN tabs 500mg |
| 94371990 | CLARITHROMYCIN paed susp 250mg/5ml |
| 94372990 | CLARITHROMYCIN paed susp 125mg/5ml |
| 94426990 | ERYTHROMYCIN ec tab 250mg |
| 94457992 | ERYCEN 125 MG SUS |
| 94495998 | ERYTHROMYCIN SPRINKLE caps 125mg |
| 94496998 | ERYTHROMYCIN SPRINKLE caps 125mg |
| 94504990 | CLARITHROMYCIN tabs 500mg |
| 94505990 | CLARITHROMYCIN tabs 250mg |
| 94512996 | AZITHROMYCIN tabs 500mg |
| 94512997 | AZITHROMYCIN susp 40mg/ml |
| 94512998 | AZITHROMYCIN caps 250mg |
| 94523996 | AZITHROMYCIN tabs 500mg |
| 94523997 | AZITHROMYCIN susp 40mg/ml |
| 94523998 | AZITHROMYCIN caps 250mg |
| 94529990 | CLARITHROMYCIN tabs 500mg |
| 94530990 | CLARITHROMYCIN tabs 250mg |
| 94530996 | CLARITHROMYCIN tabs 500mg |
| 94530997 | CLARITHROMYCIN paed susp 125mg/5ml |
| 94530998 | CLARITHROMYCIN tabs 250mg |
| 94531996 | CLARITHROMYCIN tabs 500mg |
| 94531997 | CLARITHROMYCIN paed susp 125mg/5ml |
| 94531998 | CLARITHROMYCIN tabs 250mg |
| 94541990 | CLARITHROMYCIN tabs 500mg |
| 94542990 | CLARITHROMYCIN tabs 250mg |
| 94550990 | CLARITHROMYCIN tabs 500mg |
| 94551990 | CLARITHROMYCIN tabs 250mg |
| 94693990 | ERYTHROMYCIN ETHYLSUCCINATE sf susp 250mg/5ml |
| 94694990 | ERYTHROMYCIN ETHYLSUCCINATE sf susp 125mg/5ml |
| 94819997 | ERYTHROMYCIN ETHYLSUCCINATE tabs 500mg |
| 94819998 | ERYTHROMYCIN ETHYLSUCCINATE sach 1g |
| 94820996 | ERYTHROMYCIN ETHYLSUCCINATE sach 500mg |
| 94820997 | ERYTHROMYCIN ETHYLSUCCINATE tabs 500mg |
| 94820998 | ERYTHROMYCIN ETHYLSUCCINATE sach 1g |
| 94977992 | ERYMAX 500 MG CAP |
| 94978992 | ERYMAX 125 MG SYR |
| 94979992 | ERMYSIN E/C 500 MG TAB |
| 94980992 | ERYTHROMYCIN 500 MG CAP |
| 94984992 | ERYTHROPED 250 MG TAB |
| 95305990 | ERYTHROMYCIN ec tab 250mg |
| 95920990 | ERYTHROMYCIN caps(ec grans) 250mg |
| 95965990 | ERYTHROMYCIN caps(ec grans) 250mg |
| 96175990 | ERYTHROMYCIN ETHYLSUCCINATE susp 500mg/5ml |
| 96176990 | ERYTHROMYCIN ETHYLSUCCINATE susp 250mg/5ml |
| 96177990 | ERYTHROMYCIN ETHYLSUCCINATE susp 125mg/5ml |
| 96229992 | ERYTHROMYCIN 100 MG SYR |
| 96230992 | ERYTHROCIN 100 MG SYR |
| 96250990 | ERYTHROMYCIN ETHYLSUCCINATE sf susp 500mg/5ml |
| 96309992 | KLARICID IV 500MG VIAL DRY 500 MG INJ |
| 96352988 | ERYTHROMYCIN ETHYLSUCCINATE sf susp 250mg/5ml |
| 96352989 | ERYTHROMYCIN ETHYLSUCCINATE sf susp 125mg/5ml |
| 96352990 | ERYTHROMYCIN ec tab 250mg |
| 96373990 | ERYTHROMYCIN ec tab 250mg |
| 96374988 | ERYTHROMYCIN ETHYLSUCCINATE susp 500mg/5ml |
| 96374989 | ERYTHROMYCIN ETHYLSUCCINATE susp 250mg/5ml |
| 96374990 | ERYTHROMYCIN ETHYLSUCCINATE susp 125mg/5ml |
| 96466992 | SPIRAMYCIN 250 MG TAB |
| 96648992 | ERYTHROMYCIN 12 MG SYR |
| 96649992 | ERYTHROMYCIN I/V 300 MG INJ |
| 96650990 | ERYTHROMYCIN LACTOBIONATE pow conc sol inf 1g |
| 96721988 | ERYTHROMYCIN ETHYLSUCCINATE sf susp 125mg/5ml |
| 96721989 | ERYTHROMYCIN ETHYLSUCCINATE sf susp 500mg/5ml |
| 96721990 | ERYTHROMYCIN ETHYLSUCCINATE sf susp 250mg/5ml |
| 96781997 | ERYTHROMYCIN STEARATE tabs 500mg |
| 96781998 | ERYTHROMYCIN STEARATE tabs 250mg |
| 96782998 | ERYTHROMYCIN LACTOBIONATE pow conc sol inf 1g |
| 96783996 | ERYTHROMYCIN ETHYLSUCCINATE sf susp 250mg/5ml |
| 96783997 | ERYTHROMYCIN ETHYLSUCCINATE sf susp 125mg/5ml |
| 96783998 | ERYTHROMYCIN ETHYLSUCCINATE sf grans 250mg |
| 96784996 | ERYTHROMYCIN ETHYLSUCCINATE susp 500mg/5ml |
| 96784997 | ERYTHROMYCIN ETHYLSUCCINATE susp 250mg/5ml |
| 96784998 | ERYTHROMYCIN ETHYLSUCCINATE susp 125mg/5ml |
| 96785996 | ERYTHROMYCIN ESTOLATE susp 125mg/5ml |
| 96785997 | ERYTHROMYCIN ESTOLATE tabs 500mg |
| 96785998 | ERYTHROMYCIN ESTOLATE caps 250mg |
| 96786997 | ERYTHROMYCIN ec tab 500mg |
| 96786998 | ERYTHROMYCIN ec tab 250mg |
| 97095996 | ERYTHROMYCIN ETHYLSUCCINATE susp 500mg/5ml |
| 97095997 | ERYTHROMYCIN ETHYLSUCCINATE susp 250mg/5ml |
| 97095998 | ERYTHROMYCIN ETHYLSUCCINATE susp 125mg/5ml |
| 97096998 | ERYTHROMYCIN ec tab 250mg |
| 97117996 | ERYTHROMYCIN susp 500mg/5ml |
| 97117997 | ERYTHROMYCIN susp 250mg/5ml |
| 97117998 | ERYTHROMYCIN susp 125mg/5ml |
| 97118996 | ERYTHROMYCIN caps(ec grans) 250mg |
| 97118997 | ERYTHROMYCIN ec tab 500mg |
| 97118998 | ERYTHROMYCIN ec tab 250mg |
| 97119988 | ERYTHROMYCIN ETHYLSUCCINATE susp 500mg/5ml |
| 97119989 | ERYTHROMYCIN ETHYLSUCCINATE susp 250mg/5ml |
| 97119990 | ERYTHROMYCIN ETHYLSUCCINATE susp 125mg/5ml |
| 97360998 | ERYTHROMYCIN ETHYLSUCCINATE susp 250mg/5ml |
| 97361997 | ERYTHROMYCIN STEARATE tabs 500mg |
| 97361998 | ERYTHROMYCIN STEARATE tabs 250mg |
| 97366992 | ERYTHROCIN 300 MG INJ |
| 97375992 | ERYTHROMYCIN 250 MG MIX |
| 97376992 | ERYTHROMYCIN I/V 1 GM INJ |
| 97377992 | ERYTHROMYCIN 50 MG INJ |
| 97378992 | ERYTHROCIN A 1 GM TAB |
| 97379992 | ERYTHROCIN 125 MG SYR |
| 97380992 | ERYTHROCIN 250 250 MG TAB |
| 97381992 | ERYTHROCIN B-PACK 10 FILMTABS 500 MG TAB |
| 97519998 | ERYTHROMYCIN ETHYLSUCCINATE sf grans 250mg |
| 97757989 | ERYTHROMYCIN ETHYLSUCCINATE susp 500mg/5ml |
| 97757990 | ERYTHROMYCIN ESTOLATE caps 250mg |
| 97759988 | ERYTHROMYCIN ETHYLSUCCINATE susp 250mg/5ml |
| 97759989 | ERYTHROMYCIN ETHYLSUCCINATE susp 125mg/5ml |
| 97759990 | ERYTHROMYCIN ec tab 250mg |
| 97887990 | ERYTHROMYCIN LACTOBIONATE pow conc sol inf 1g |
| 97898990 | ERYTHROMYCIN ETHYLSUCCINATE sf susp 500mg/5ml |
| 97932998 | ERYTHROMYCIN caps(ec grans) 250mg |
| 97947988 | ERYTHROMYCIN ETHYLSUCCINATE sf susp 250mg/5ml |
| 97947989 | ERYTHROMYCIN ETHYLSUCCINATE sf susp 125mg/5ml |
| 97947990 | ERYTHROMYCIN ETHYLSUCCINATE susp 500mg/5ml |
| 97994992 | ROVAMYCIN 250 MG TAB |
| 98166988 | ERYTHROMYCIN ec tab 250mg |
| 98166989 | ERYTHROMYCIN ec tab 500mg |
| 98166990 | ERYTHROMYCIN ETHYLSUCCINATE sf susp 500mg/5ml |
| 98345989 | ERYTHROMYCIN ETHYLSUCCINATE sf susp 500mg/5ml |
| 98345990 | ERYTHROMYCIN ec tab 500mg |
| 98353988 | ERYTHROMYCIN ec tab 250mg |
| 98353989 | ERYTHROMYCIN ETHYLSUCCINATE sf susp 125mg/5ml |
| 98353990 | ERYTHROMYCIN ETHYLSUCCINATE sf susp 250mg/5ml |
| 98557988 | ERYTHROMYCIN ETHYLSUCCINATE susp 500mg/5ml |
| 98557989 | ERYTHROMYCIN ETHYLSUCCINATE susp 125mg/5ml |
| 98557990 | ERYTHROMYCIN ETHYLSUCCINATE susp 250mg/5ml |
| 98558990 | ERYTHROMYCIN ec tab 500mg |
| 98559988 | ERYTHROMYCIN ec tab 250mg |
| 98559989 | ERYTHROMYCIN ETHYLSUCCINATE susp 250mg/5ml |
| 98559990 | ERYTHROMYCIN ETHYLSUCCINATE susp 125mg/5ml |
| 98751996 | ERYTHROMYCIN ETHYLSUCCINATE sf susp 500mg/5ml |
| 98751997 | ERYTHROMYCIN ETHYLSUCCINATE sach 500mg |
| 98751998 | ERYTHROMYCIN ETHYLSUCCINATE susp 500mg/5ml |
| 98752998 | ERYTHROMYCIN ec tab 500mg |
| 98753998 | ERYTHROMYCIN LACTOBIONATE pow conc sol inf 1g |
| 98754998 | ERYTHROMYCIN STEARATE tabs 500mg |
| 98846998 | ERYTHROMYCIN ESTOLATE susp 250mg/5ml |
| 99103996 | ERYTHROMYCIN ec tab 500mg |
| 99103997 | ERYTHROMYCIN ec tab 250mg |
| 99103998 | ERYTHROMYCIN ETHYLSUCCINATE sf susp 125mg/5ml |
| 99210989 | ERYTHROMYCIN ETHYLSUCCINATE susp 500mg/5ml |
| 99210990 | ERYTHROMYCIN ec tab 500mg |
| 99212990 | ERYTHROMYCIN ETHYLSUCCINATE susp 500mg/5ml |
| 99433990 | ERYTHROMYCIN ec tab 500mg |
| 99434990 | ERYTHROMYCIN ec tab 500mg |
| 99435989 | ERYTHROMYCIN ec tab 500mg |
| 99435990 | ERYTHROMYCIN ec tab 250mg |
| 99540998 | ERYTHROMYCIN ec tab 250mg |
| 99541998 | ERYTHROMYCIN ESTOLATE susp 250mg/5ml |
| 99542996 | ERYTHROMYCIN ESTOLATE susp 125mg/5ml |
| 99542997 | ERYTHROMYCIN ec tab 500mg |
| 99542998 | ERYTHROMYCIN ESTOLATE caps 250mg |
| 99604988 | ERYTHROMYCIN ec tab 250mg |
| 99604989 | ERYTHROMYCIN ETHYLSUCCINATE susp 250mg/5ml |
| 99604990 | ERYTHROMYCIN ETHYLSUCCINATE susp 125mg/5ml |
| 99605988 | ERYTHROMYCIN ec tab 250mg |
| 99605989 | ERYTHROMYCIN ETHYLSUCCINATE susp 250mg/5ml |
| 99605990 | ERYTHROMYCIN ETHYLSUCCINATE susp 125mg/5ml |
| 99606988 | ERYTHROMYCIN ETHYLSUCCINATE susp 500mg/5ml |
| 99606989 | ERYTHROMYCIN ETHYLSUCCINATE susp 250mg/5ml |
| 99606990 | ERYTHROMYCIN ETHYLSUCCINATE susp 125mg/5ml |
| 99607988 | ERYTHROMYCIN ec tab 250mg |
| 99607989 | ERYTHROMYCIN ETHYLSUCCINATE susp 250mg/5ml |
| 99607990 | ERYTHROMYCIN ETHYLSUCCINATE susp 125mg/5ml |
| 99679996 | ERYTHROMYCIN ETHYLSUCCINATE sf susp 125mg/5ml |
| 99679997 | ERYTHROMYCIN ETHYLSUCCINATE sach 125mg |
| 99679998 | ERYTHROMYCIN ETHYLSUCCINATE susp 125mg/5ml |
| 99680996 | ERYTHROMYCIN ETHYLSUCCINATE sf susp 250mg/5ml |
| 99680997 | ERYTHROMYCIN ETHYLSUCCINATE sach 250mg |
| 99680998 | ERYTHROMYCIN ETHYLSUCCINATE susp 250mg/5ml |
| 99681998 | ERYTHROMYCIN ec tab 250mg |
| 99682998 | ERYTHROMYCIN STEARATE tabs 250mg |
| 99683997 | ERYTHROMYCIN ec tab 500mg |
| 99683998 | ERYTHROMYCIN ec tab 250mg |
| 83940998 | METRONIDAZOLE oral liq |
| 86711998 | METRONIDAZOLE inf 100mg/20ml |
| 86712998 | METRONIDAZOLE inf 500mg/100ml |
| 86713998 | METRONIDAZOLE inf 500mg/100ml |
| 86714998 | METRONIDAZOLE inf 100mg/20ml |
| 89786998 | METRONIDAZOLE tabs 500mg |
| 90802998 | NYSTATIN + METRONIDAZOLE vaginal inserts+tabs 100,000iu + 400mg |
| 90803998 | METRONIDAZOLE + NYSTATIN tabs+vaginal inserts 400mg + 100,000iu |
| 92036998 | METRONIDAZOLE inf 500mg/100ml |
| 92227990 | METRONIDAZOLE tabs 500mg |
| 92561990 | METRONIDAZOLE inf 500mg/100ml |
| 92806998 | METRONIDAZOLE susp 200mg/5ml |
| 93349990 | METRONIDAZOLE tabs 400mg |
| 93350990 | METRONIDAZOLE tabs 200mg |
| 93594998 | METRONIDAZOLE susp 200mg/5ml |
| 93609990 | METRONIDAZOLE inf 500mg/100ml |
| 93726992 | FASIGYN I/V 2 MG SOL |
| 93874992 | FLAGYL GEL |
| 93936997 | METRONIDAZOLE supp 1g |
| 93936998 | METRONIDAZOLE supp 500mg |
| 94156990 | METRONIDAZOLE tabs 400mg |
| 94385998 | METRONIDAZOLE inf 5mg/ml |
| 94516990 | METRONIDAZOLE tabs 400mg |
| 94517990 | METRONIDAZOLE tabs 200mg |
| 94534990 | METRONIDAZOLE tabs 400mg |
| 94535990 | METRONIDAZOLE tabs 200mg |
| 95158997 | TINIDAZOLE inf 2mg/ml |
| 95158998 | TINIDAZOLE tabs 500mg |
| 95295990 | METRONIDAZOLE tabs 200mg |
| 95816998 | METRONIDAZOLE inf 5mg/ml |
| 95817997 | METRONIDAZOLE supp 1g |
| 95817998 | METRONIDAZOLE supp 500mg |
| 95818997 | METRONIDAZOLE tabs 400mg |
| 95818998 | METRONIDAZOLE tabs 200mg |
| 95819998 | METRONIDAZOLE tabs 200mg |
| 95820996 | METRONIDAZOLE inf 500mg/100ml |
| 95820997 | METRONIDAZOLE tabs 400mg |
| 95820998 | METRONIDAZOLE tabs 200mg |
| 95821997 | METRONIDAZOLE supp 1g |
| 95821998 | METRONIDAZOLE supp 500mg |
| 95822997 | METRONIDAZOLE supp 1g |
| 95822998 | METRONIDAZOLE supp 500mg |
| 95823997 | METRONIDAZOLE treatment pack |
| 95823998 | METRONIDAZOLE tabs 400mg |
| 96248990 | METRONIDAZOLE inf 100mg/20ml |
| 96309989 | METRONIDAZOLE tabs 400mg |
| 96309990 | METRONIDAZOLE tabs 200mg |
| 96483990 | METRONIDAZOLE inf 500mg/100ml |
| 96574990 | METRONIDAZOLE susp 200mg/5ml |
| 96711992 | METRONIDAZOLE TAB/NYSTATIN PES COMBIPACK |
| 96847989 | METRONIDAZOLE tabs 400mg |
| 96847990 | METRONIDAZOLE tabs 200mg |
| 97020998 | METRONIDAZOLE tabs 200mg |
| 97108989 | METRONIDAZOLE supp 1g |
| 97108990 | METRONIDAZOLE supp 500mg |
| 97748992 | METRONIDAZOLE .5 % SOL |
| 97749992 | METRONIDAZOLE .9 % SOL |
| 97752992 | METRONIDAZOLE POW |
| 97753992 | METRONIDAZOLE 200 MG MIX |
| 97879990 | METRONIDAZOLE tabs 500mg |
| 98320998 | METRONIDAZOLE inf 500mg/100ml |
| 98321996 | METRONIDAZOLE supp 1g |
| 98321997 | METRONIDAZOLE supp 500mg |
| 98321998 | METRONIDAZOLE tabs 200mg |
| 98439998 | METRONIDAZOLE inf 500mg/100ml |
| 98871998 | METRONIDAZOLE susp 200mg/5ml |
| 99434998 | METRONIDAZOLE inf 500mg/100ml |
| 99495988 | METRONIDAZOLE susp 200mg/5ml |
| 99495989 | METRONIDAZOLE tabs 400mg |
| 99495990 | METRONIDAZOLE tabs 200mg |
| 99496989 | METRONIDAZOLE tabs 400mg |
| 99496990 | METRONIDAZOLE tabs 200mg |
| 99497989 | METRONIDAZOLE tabs 400mg |
| 99497990 | METRONIDAZOLE tabs 200mg |
| 99498988 | METRONIDAZOLE supp 1g |
| 99498989 | METRONIDAZOLE supp 500mg |
| 99498990 | METRONIDAZOLE tabs 200mg |
| 99499989 | METRONIDAZOLE tabs 400mg |
| 99499990 | METRONIDAZOLE tabs 200mg |
| 99639997 | METRONIDAZOLE tabs 400mg |
| 99639998 | METRONIDAZOLE tabs 200mg |
| 99640998 | METRONIDAZOLE + NYSTATIN tabs+vaginal inserts 400mg + 100,000iu |
| 99656998 | TINIDAZOLE tabs 500mg |
| 87274998 | CEFUROXIME + METRONIDAZOLE inf 1.5g + 500mg |
| 88007998 | LANSOPRAZOLE + AMOXICILLIN & CLARITHROMYCIN triple pack 30mg + 500mg + 500mg |
| 88008998 | CLARITHROMYCIN + LANSOPRAZOLE & AMOXICILLIN triple pack 500mg + 30mg + 500mg |
| 92073998 | AMOXICILLIN + CLARITHROMYCIN & LANSOPRAZOLE triple pack 500mg + 500mg + 30mg |
| 92075998 | AMOXICILLIN + CLARITHROMYCIN & LANSOPRAZOLE triple pack 500mg + 500mg + 30mg |
| 94752990 | CEFUROXIME + METRONIDAZOLE inf 1.5g + 500mg |
| 98310998 | CLARITHROMYCIN + METRONIDAZOLE & LANSOPRAZOLE triple pack 500mg + 400mg + 30mg |
| 99218998 | CLARITHROMYCIN + METRONIDAZOLE & LANSOPRAZOLE triple pack 500mg + 400mg + 30mg |
| 82592998 | FLUCLOXACILLIN sf oral soln 125mg/5ml |
| 82593998 | FLUCLOXACILLIN sf oral soln 250mg/5ml |
| 82935998 | FLUCLOXACILLIN caps 500mg |
| 82936998 | FLUCLOXACILLIN caps 250mg |
| 84355998 | AMPICILLIN susp 250mg/5ml |
| 84356998 | AMPICILLIN susp 125mg/5ml |
| 84786998 | PHENOXYMETHYLPENICILLIN (AS CALCIUM SALT) (IPU) tabs 666mg |
| 84787998 | PHENOXYMETHYLPENICILLIN (AS CALCIUM SALT) (IPU) tabs 333mg |
| 84788998 | PHENOXYMETHYLPENICILLIN (AS CALCIUM SALT) (IPU) oral susp 250mg/5ml |
| 85182998 | AMOXICILLIN + CLAVULANIC ACID (IPU) oral susp 125mg + 62.5mg/5ml |
| 85277998 | AMOXICILLIN + CLAVULANIC ACID (IPU) tabs 875mg + 125mg |
| 86034998 | CLAVULANIC ACID + AMOXICILLIN oral susp 31mg + 125mg/5ml |
| 86035998 | AMOXICILLIN + CLAVULANIC ACID oral susp 125mg + 31mg/5ml |
| 86036998 | CLAVULANIC ACID + AMOXICILLIN oral susp 62mg + 250mg/5ml |
| 86037998 | AMOXICILLIN + CLAVULANIC ACID oral susp 250mg + 62mg/5ml |
| 86055998 | CO-AMOXICLAV oral susp 125mg + 31mg/5ml |
| 86056998 | CO-AMOXICLAV oral susp 250mg + 62mg/5ml |
| 86418998 | TEMOCILLIN pwdr/inj.soln 1g/vial |
| 86675998 | AMPICILLIN caps 250mg |
| 86777998 | AMPICILLIN caps 500mg |
| 86912998 | PENICILLIN V sf oral soln 250mg/5ml |
| 86913998 | PHENOXYMETHYLPENICILLIN sf oral soln 250mg/5ml |
| 86914998 | PENICILLIN V sf oral soln 125mg/5ml |
| 86915998 | PHENOXYMETHYLPENICILLIN sf oral soln 125mg/5ml |
| 88382998 | AMOXICILLIN sf oral susp 250mg/5ml |
| 88536998 | CO-AMOXICLAV tabs 250mg+125mg |
| 88548996 | FLUCLOXACILLIN elixir 125mg/5ml |
| 88548997 | FLUCLOXACILLIN caps 500mg |
| 88548998 | FLUCLOXACILLIN caps 250mg |
| 88549996 | AMPICILLIN susp 125mg/5ml |
| 88549997 | AMPICILLIN caps 500mg |
| 88549998 | AMPICILLIN caps 250mg |
| 88556997 | AMOXICILLIN caps 500mg |
| 88556998 | AMOXICILLIN caps 250mg |
| 88557996 | AMOXICILLIN sf oral susp 125mg/5ml |
| 88557997 | AMOXICILLIN syrp 250mg/5ml |
| 88557998 | AMOXICILLIN syrp 125mg/5ml |
| 89177997 | AMOXICILLIN caps 500mg |
| 89177998 | AMOXICILLIN caps 250mg |
| 89520997 | FLUCLOXACILLIN caps 500mg |
| 89520998 | FLUCLOXACILLIN caps 250mg |
| 89889997 | AMOXICILLIN sf oral susp 250mg/5ml |
| 89889998 | AMOXICILLIN sf oral susp 125mg/5ml |
| 90855998 | BENZYLPENICILLIN SODIUM + BENEMETHAMINE PENICILLIN & PROCAINE BENZYLPENICILLIN inj |
| 90856997 | BENZYLPENICILLIN pwdr/inj.soln 1200mg |
| 90856998 | BENZYLPENICILLIN inj 6g/vial |
| 90857998 | BENZYLPENICILLIN intrathec inj 12mg/vial |
| 90862997 | PHENOXYMETHYLPENICILLIN oral soln 250mg/5ml |
| 90862998 | PHENOXYMETHYLPENICILLIN oral soln 125mg/5ml |
| 90863997 | PHENOXYMETHYLPENICILLIN syrp 62.5mg/5ml |
| 90863998 | PHENOXYMETHYLPENICILLIN caps 250mg |
| 90864997 | PHENOXYMETHYLPENICILLIN tabs 250mg |
| 90864998 | PHENOXYMETHYLPENICILLIN tabs 125mg |
| 91014998 | CO-AMOXICLAV sf susp 400mg + 57mg/5ml |
| 91166996 | PHENOXYMETHYLPENICILLIN oral soln 250mg/5ml |
| 91166997 | PHENOXYMETHYLPENICILLIN oral soln 125mg/5ml |
| 91166998 | PHENOXYMETHYLPENICILLIN tabs 250mg |
| 91260996 | BENZYLPENICILLIN syrp 250mg/5ml |
| 91260997 | BENZYLPENICILLIN syrp 125mg/5ml |
| 91260998 | BENZYLPENICILLIN tabs 250mg |
| 92238990 | FLUCLOXACILLIN sf oral soln 250mg/5ml |
| 92239990 | FLUCLOXACILLIN sf oral soln 125mg/5ml |
| 92308990 | FLUCLOXACILLIN sf oral soln 250mg/5ml |
| 92309990 | FLUCLOXACILLIN sf oral soln 125mg/5ml |
| 92339998 | CO-AMOXICLAV sf susp 250mg + 62mg/5ml |
| 92340996 | CO-AMOXICLAV sf susp 125mg + 31mg/5ml |
| 92340997 | CO-AMOXICLAV tabs 500mg+125mg |
| 92340998 | CO-AMOXICLAV tabs 250mg+125mg |
| 92344990 | PIPERACILLIN + TAZOBACTAM pwdr/inj.soln 4g + 500mg/vial |
| 92345990 | PIPERACILLIN + TAZOBACTAM pwdr/inj.soln 2g + 250mg/vial |
| 92395990 | CO-AMOXICLAV sf susp 250mg + 62mg/5ml |
| 92396990 | CO-AMOXICLAV sf susp 125mg + 31mg/5ml |
| 92429990 | PIPERACILLIN + TAZOBACTAM pwdr/inj.soln 4g + 500mg/vial |
| 92430990 | PIPERACILLIN + TAZOBACTAM pwdr/inj.soln 2g + 250mg/vial |
| 92455990 | AMOXICILLIN caps 500mg |
| 92456990 | AMOXICILLIN caps 250mg |
| 92973997 | AMPICILLIN inj 500mg |
| 92973998 | AMPICILLIN inj 250mg |
| 92974997 | FLUCLOXACILLIN elixir 125mg/5ml |
| 92974998 | FLUCLOXACILLIN pwdr/inj.soln 500mg/vial |
| 92990990 | PHENOXYMETHYLPENICILLIN sf oral soln 250mg/5ml |
| 92991990 | PHENOXYMETHYLPENICILLIN sf oral soln 125mg/5ml |
| 93040990 | AMOXICILLIN sf oral susp 250mg/5ml |
| 93041990 | AMOXICILLIN sf oral susp 125mg/5ml |
| 93108997 | PIPERACILLIN + TAZOBACTAM pwdr/inj.soln 4g + 500mg/vial |
| 93108998 | PIPERACILLIN + TAZOBACTAM pwdr/inj.soln 2g + 250mg/vial |
| 93109997 | PIPERACILLIN + TAZOBACTAM pwdr/inj.soln 4g + 500mg/vial |
| 93109998 | PIPERACILLIN + TAZOBACTAM pwdr/inj.soln 2g + 250mg/vial |
| 93150998 | PROCAINE BENZYLPENICILLIN inj |
| 93213998 | CO-FLUAMPICIL inj 500mg+500mg |
| 93214996 | CO-FLUAMPICIL inj 250mg+250mg |
| 93214997 | CO-FLUAMPICIL syrp 125mg+125mg |
| 93214998 | CO-FLUAMPICIL caps 250mg+250mg |
| 93224996 | CO-AMOXICLAV sf susp 400mg + 57mg/5ml |
| 93224997 | CO-AMOXICLAV tabs 500mg+125mg |
| 93224998 | CO-AMOXICLAV sf susp 250mg + 62mg/5ml |
| 93225996 | CO-AMOXICLAV sf susp 125mg + 31mg/5ml |
| 93225997 | CO-AMOXICLAV disp tab 250mg+125mg |
| 93225998 | CO-AMOXICLAV tabs 250mg+125mg |
| 93253992 | AMOXIL SF 125 MG/5ML SYR |
| 93372996 | AMOXICILLIN sf oral susp 125mg/5ml |
| 93372997 | AMOXICILLIN caps 500mg |
| 93372998 | AMOXICILLIN caps 250mg |
| 93375998 | AMOXICILLIN sf oral susp 250mg/5ml |
| 93377996 | AMOXICILLIN sf oral susp 125mg/5ml |
| 93377997 | AMOXICILLIN caps 500mg |
| 93377998 | AMOXICILLIN caps 250mg |
| 93394996 | AMPICILLIN + SULBACTAM inj 2g + 1g |
| 93394997 | AMPICILLIN + SULBACTAM inj 1g + 500mg |
| 93394998 | AMPICILLIN + SULBACTAM inj 500mg + 250mg |
| 93395996 | AMPICILLIN + SULBACTAM inj 2g + 1g |
| 93395997 | AMPICILLIN + SULBACTAM inj 1g + 500mg |
| 93395998 | AMPICILLIN + SULBACTAM inj 500mg + 250mg |
| 93404998 | AMOXICILLIN sf oral susp 250mg/5ml |
| 93405992 | PIPRIL 2 MG INJ |
| 93407996 | AMOXICILLIN sf oral susp 125mg/5ml |
| 93407997 | AMOXICILLIN caps 500mg |
| 93407998 | AMOXICILLIN caps 250mg |
| 93446998 | AMOXICILLIN sf oral susp 250mg/5ml |
| 93465996 | AMOXICILLIN syrp 125mg/5ml |
| 93465997 | AMOXICILLIN caps 500mg |
| 93465998 | AMOXICILLIN caps 250mg |
| 93466998 | AMOXICILLIN syrp 250mg/5ml |
| 93543997 | AZLOCILLIN inf 5g/vial |
| 93543998 | AZLOCILLIN inj 2g/vial |
| 93548990 | CO-AMOXICLAV inj 1000mg+200mg |
| 93549990 | CO-AMOXICLAV inj 500mg+100mg |
| 93556998 | BENZYLPENICILLIN SODIUM + PROCAINE BENZYLPENICILLIN inj |
| 93564998 | PROCAINE BENZYLPENICILLIN + BENZYLPENICILLIN SODIUM inj |
| 93567990 | CO-AMOXICLAV inj 1000mg+200mg |
| 93568990 | CO-AMOXICLAV inj 500mg+100mg |
| 93588990 | PHENOXYMETHYLPENICILLIN oral soln 250mg/5ml |
| 93589990 | PHENOXYMETHYLPENICILLIN oral soln 125mg/5ml |
| 93591990 | AMOXICILLIN sf oral susp 250mg/5ml |
| 93592990 | AMOXICILLIN sf oral susp 125mg/5ml |
| 93601997 | TEMOCILLIN pwdr/inj.soln 1g/vial |
| 93601998 | TEMOCILLIN sterile pwdr 500mg/vial |
| 93602997 | TEMOCILLIN pwdr/inj.soln 1g/vial |
| 93602998 | TEMOCILLIN sterile pwdr 500mg/vial |
| 93629992 | BENETHAMINE PENICILLIN G /PEN.G SODIUM/ 475 MG INJ |
| 93686992 | AMOXIDIN 250 MG CAP |
| 93736992 | CICLACILLIN 125 MG SYR |
| 93737992 | CRYSTAPEN (SOD SALT) 3 GM INJ |
| 93763990 | CO-FLUAMPICIL caps 250mg+250mg |
| 93786990 | PHENOXYMETHYLPENICILLIN tabs 250mg |
| 93787990 | PHENOXYMETHYLPENICILLIN sf oral soln 250mg/5ml |
| 93788990 | PHENOXYMETHYLPENICILLIN sf oral soln 125mg/5ml |
| 93849997 | CO-AMOXICLAV inj 1000mg+200mg |
| 93849998 | CO-AMOXICLAV inj 500mg+100mg |
| 93890990 | PHENOXYMETHYLPENICILLIN tabs 250mg |
| 93910990 | FLUCLOXACILLIN pwdr/inj.soln 1g/vial |
| 93911990 | FLUCLOXACILLIN pwdr/inj.soln 500mg/vial |
| 93929990 | AMPICILLIN inj 500mg |
| 93949992 | PENIDURAL LA INJ |
| 94014992 | V-CIL-K PEDIPACS SACHETS 125 MG |
| 94025990 | CO-AMOXICLAV sf susp 250mg + 62mg/5ml |
| 94026990 | CO-AMOXICLAV sf susp 125mg + 31mg/5ml |
| 94027990 | CO-AMOXICLAV tabs 250mg+125mg |
| 94054990 | PHENOXYMETHYLPENICILLIN sf oral soln 250mg/5ml |
| 94055990 | PHENOXYMETHYLPENICILLIN sf oral soln 125mg/5ml |
| 94068992 | AMOXIDIN 500 MG CAP |
| 94069992 | AMOXYCILLIN 125 MG CAP |
| 94070990 | AMOXICILLIN syrp 250mg/5ml |
| 94070992 | AMPILAR 250 MG SYR |
| 94071990 | AMOXICILLIN syrp 125mg/5ml |
| 94073990 | AMOXICILLIN caps 500mg |
| 94074990 | AMOXICILLIN caps 250mg |
| 94113990 | PHENOXYMETHYLPENICILLIN tabs 250mg |
| 94126992 | CRYSTAPEN V 250 MG TAB |
| 94127992 | CRYSTAPEN V 125 MG SUS |
| 94128992 | CRYSTAPEN (SOD SALT) 6 GM INJ |
| 94129992 | CRYSTAPEN (SOD SALT) 300 GM INJ |
| 94154990 | FLUCLOXACILLIN caps 500mg |
| 94155990 | FLUCLOXACILLIN caps 250mg |
| 94157996 | BENZYLPENICILLIN inj 3g/vial |
| 94157997 | BENZYLPENICILLIN pwdr/inj.soln 600mg/vial |
| 94157998 | BENZYLPENICILLIN inj 300mg/vial |
| 94189990 | PHENOXYMETHYLPENICILLIN sf oral soln 250mg/5ml |
| 94190990 | PHENOXYMETHYLPENICILLIN sf oral soln 125mg/5ml |
| 94233990 | PHENOXYMETHYLPENICILLIN sf oral soln 250mg/5ml |
| 94234990 | PHENOXYMETHYLPENICILLIN sf oral soln 125mg/5ml |
| 94277997 | FLUCLOXACILLIN caps 500mg |
| 94277998 | FLUCLOXACILLIN caps 250mg |
| 94296990 | CO-FLUAMPICIL caps 250mg+250mg |
| 94316992 | PROCAINE PENICILLIN/BENZYLPENICILLIN 3 GM INJ |
| 94325992 | PENICILLIN G 250 MG SYR |
| 94363992 | TEMOPEN 500 MG INJ |
| 94394992 | ABBOFLOX 250 MG CAP |
| 94413990 | PHENOXYMETHYLPENICILLIN tabs 250mg |
| 94455990 | FLUCLOXACILLIN caps 500mg |
| 94457990 | FLUCLOXACILLIN caps 250mg |
| 94519992 | PENBRITIN PAED 125 MG TAB |
| 94532992 | PENICILLIN G 125 MG SYR |
| 94533992 | PENICILLIN V 500 MG TAB |
| 94641992 | AMPILAR 500 MG CAP |
| 94642992 | AMPILAR 250 MG CAP |
| 94643992 | AMPILAR 125 MG SYR |
| 94651997 | FLUCLOXACILLIN pwdr/inj.soln 1g/vial |
| 94651998 | FLUCLOXACILLIN pwdr/inj.soln 500mg/vial |
| 94652996 | FLUCLOXACILLIN pwdr/inj.soln 250mg/vial |
| 94652997 | FLUCLOXACILLIN caps 500mg |
| 94652998 | FLUCLOXACILLIN caps 250mg |
| 94664996 | CLOXACILLIN + AMPICILLIN susp 30mg + 60mg/0.6ml |
| 94664997 | CLOXACILLIN + AMPICILLIN inj 25mg + 50mg |
| 94664998 | CLOXACILLIN + AMPICILLIN inj 250mg + 250mg |
| 94698996 | AMOXICILLIN inj 500mg |
| 94698997 | AMOXICILLIN inj 250mg |
| 94698998 | AMOXICILLIN syrp 250mg/5ml |
| 94699996 | AMOXICILLIN syrp 125mg/5ml |
| 94699997 | AMOXICILLIN caps 500mg |
| 94699998 | AMOXICILLIN caps 250mg |
| 94738990 | CO-AMOXICLAV inj 500mg+100mg |
| 94742990 | CO-AMOXICLAV inj 1000mg+200mg |
| 94744992 | BROXIL 250 MG TAB |
| 94759992 | CALTHOR 250 MG SYR |
| 94760992 | CALTHOR 125 MG SYR |
| 94762997 | PENICILLIN V elixir 250mg/5ml |
| 94762998 | PENICILLIN V elixir 125mg/5ml |
| 94792997 | AMOXICILLIN sf oral susp 250mg/5ml |
| 94792998 | AMOXICILLIN sf oral susp 125mg/5ml |
| 94794997 | AMOXICILLIN syrp 250mg/5ml |
| 94794998 | AMOXICILLIN syrp 125mg/5ml |
| 94828992 | CICLACILLIN 250 MG SYR |
| 94845996 | PENICILLIN V syrp 250mg/5ml |
| 94845997 | PENICILLIN V syrp 62.5mg/5ml |
| 94845998 | PENICILLIN V caps 250mg |
| 94871998 | PIVMECILLINAM + PIVAMPICILLIN tabs 200mg + 250mg |
| 94872998 | PIVMECILLINAM + PIVAMPICILLIN tabs 200mg + 250mg |
| 94875992 | CRYSTAPEN V 125 MG TAB |
| 94876992 | CRYSTAPEN V 250 MG SYR |
| 94877992 | CRYSTAPEN V 125 MG SYR |
| 94878992 | CRYSTAPEN G 250 MG SYR |
| 94879992 | CRYSTAPEN G 125 MG SYR |
| 94898992 | DEPOCILLIN 3 INJ |
| 94922996 | CLAVULANIC ACID + TICARCILLIN inf 200mg + 3g |
| 94922997 | CLAVULANIC ACID + TICARCILLIN inf 100mg + 1.5g |
| 94922998 | CLAVULANIC ACID + TICARCILLIN inf 50mg + 750mg |
| 94923996 | TICARCILLIN + CLAVULANIC ACID inf 750mg + 50mg |
| 94923997 | TICARCILLIN + CLAVULANIC ACID inf 1.5g + 100mg |
| 94923998 | TICARCILLIN + CLAVULANIC ACID pow conc sol inf 3g + 200mg |
| 94926990 | PHENOXYMETHYLPENICILLIN tabs 250mg |
| 94936992 | DISTAQUAINE V-K (DQV-K) 125 MG TAB |
| 94936996 | AMPICILLIN + CLOXACILLIN syrp 250mg/5ml |
| 94936997 | AMPICILLIN + CLOXACILLIN caps 500mg |
| 94936998 | AMPICILLIN + CLOXACILLIN inj 250mg + 250mg/vial |
| 94945998 | AMOXICILLIN inj 1g |
| 95009998 | BENETHAMINE PENICILLIN + PROCAINE BENZYLPENICILLIN & BENZYLPENICILLIN SODIUM inj |
| 95027992 | FLUCLOXACILLIN 125 MG CAP |
| 95028996 | AMOXICILLIN + CLAVULANIC ACID inj 1g + 200mg/vial |
| 95028997 | AMOXICILLIN + CLAVULANIC ACID inj 500mg + 100mg/vial |
| 95028998 | AMOXICILLIN + CLAVULANIC ACID susp 250mg + 62mg/5ml |
| 95029996 | AMOXICILLIN + CLAVULANIC ACID susp 125mg + 31mg/5ml |
| 95029997 | AMOXICILLIN + CLAVULANIC ACID disp tab |
| 95029998 | AMOXICILLIN + CLAVULANIC ACID tabs 250mg + 125mg |
| 95030998 | AMPICILLIN + FLUCLOXACILLIN inj 500mg+500mg |
| 95031996 | AMPICILLIN + FLUCLOXACILLIN inj 250mg+250mg |
| 95031997 | AMPICILLIN + FLUCLOXACILLIN syrp 125mg+125mg |
| 95031998 | AMPICILLIN + FLUCLOXACILLIN caps 250mg+250mg |
| 95032997 | AMPICILLIN + CLOXACILLIN inj 50mg + 25mg/vial |
| 95032998 | AMPICILLIN + CLOXACILLIN sf susp 60mg + 30mg/0.6ml |
| 95033997 | AMPICILLIN inj 250mg |
| 95033998 | AMPICILLIN inj 500mg |
| 95034998 | AMPICILLIN paed susp 125mg/1.25ml |
| 95039990 | CO-AMOXICLAV sf susp 250mg + 62mg/5ml |
| 95040990 | CO-AMOXICLAV sf susp 125mg + 31mg/5ml |
| 95070990 | CO-AMOXICLAV tabs 500mg+125mg |
| 95076996 | CLAVULANIC ACID + AMOXICILLIN inj 200mg + 1g/vial |
| 95076997 | CLAVULANIC ACID + AMOXICILLIN inj 100mg + 500mg/vial |
| 95076998 | CLAVULANIC ACID + AMOXICILLIN susp 62mg + 125mg/5ml |
| 95077997 | AMOXICILLIN sol tab 750mg |
| 95077998 | AMOXICILLIN sol tab 375mg |
| 95086996 | AMOXICILLIN sf pwdr 125mg |
| 95086997 | AMOXICILLIN sol tab 750mg |
| 95086998 | AMOXICILLIN sol tab 375mg |
| 95096992 | HAVAPEN 350 MG TAB |
| 95163998 | TICARCILLIN inf 5g/bottle |
| 95164996 | TICARCILLIN inj 5g/vial |
| 95164997 | TICARCILLIN inj 3g/vial |
| 95164998 | TICARCILLIN inj 1g/vial |
| 95165998 | TICARCILLIN inf 5g/bottle |
| 95166996 | TICARCILLIN inj 5g/vial |
| 95166997 | TICARCILLIN inj 3g/vial |
| 95166998 | TICARCILLIN inj 1g/vial |
| 95218997 | TALAMPICILLIN syrp 125mg/5ml |
| 95218998 | TALAMPICILLIN tabs 250mg |
| 95224990 | AMOXICILLIN caps 500mg |
| 95225990 | AMOXICILLIN caps 250mg |
| 95246990 | FLUCLOXACILLIN elixir 125mg/5ml |
| 95261990 | PHENOXYMETHYLPENICILLIN tabs 250mg |
| 95262990 | PHENOXYMETHYLPENICILLIN oral soln 250mg/5ml |
| 95263990 | PHENOXYMETHYLPENICILLIN oral soln 125mg/5ml |
| 95293990 | PHENOXYMETHYLPENICILLIN tabs 250mg |
| 95294990 | PHENOXYMETHYLPENICILLIN oral soln 125mg/5ml |
| 95314990 | CO-AMOXICLAV oral susp 250mg + 62mg/5ml |
| 95315990 | CO-AMOXICLAV oral susp 125mg + 31mg/5ml |
| 95399998 | PROCAINE BENZYLPENICILLIN + BENETHAMINE PENICILLIN & BENZYLPENICILLIN SODIUM inj |
| 95431992 | PHENOXYMETHYLPENICILLIN 300 MG TAB |
| 95489996 | PIVMECILLINAM + PIVAMPICILLIN susp 46.2mg + 62.5mg/5ml |
| 95489997 | PIVMECILLINAM + PIVAMPICILLIN tabs 200mg + 250mg |
| 95489998 | PIVMECILLINAM + PIVAMPICILLIN tabs 100mg + 125mg |
| 95490997 | PIVMECILLINAM tabs 200mg |
| 95490998 | PIVMECILLINAM susp 100mg/sachet |
| 95491998 | PIVMECILLINAM + PIVAMPICILLIN tabs 200mg + 250mg |
| 95494996 | PIVAMPICILLIN + PIVMECILLINAM susp 62.5mg + 46.2mg/5ml |
| 95494997 | PIVAMPICILLIN + PIVMECILLINAM tabs 250mg + 200mg |
| 95494998 | PIVAMPICILLIN + PIVMECILLINAM tabs 125mg + 100mg |
| 95495996 | PIVAMPICILLIN sach 175mg |
| 95495997 | PIVAMPICILLIN susp 175mg/5ml |
| 95495998 | PIVAMPICILLIN tabs 500mg |
| 95507992 | PROCAINE PENICILLIN 3 GM INJ |
| 95510996 | PIPERACILLIN inf 4g/infusion bottle |
| 95510997 | PIPERACILLIN inj 2g/vial |
| 95510998 | PIPERACILLIN inj 1g/vial |
| 95529992 | PENICILLIN G 250 MG TAB |
| 95557997 | PHENETHICILLIN syrp 125mg/5ml |
| 95557998 | PHENETHICILLIN caps 250mg |
| 95570998 | SULTAMICILLIN tabs 375mg |
| 95571998 | SULTAMICILLIN tabs 375mg |
| 95585990 | CO-AMOXICLAV tabs 500mg+125mg |
| 95586990 | CO-AMOXICLAV sf susp 250mg + 62mg/5ml |
| 95586998 | PHENOXYMETHYLPENICILLIN caps 250mg |
| 95587998 | PHENOXYMETHYLPENICILLIN oral soln 250mg/5ml |
| 95588996 | PHENOXYMETHYLPENICILLIN oral soln 125mg/5ml |
| 95588997 | PHENOXYMETHYLPENICILLIN syrp 62.5mg/5ml |
| 95588998 | PHENOXYMETHYLPENICILLIN tabs 250mg |
| 95591998 | PENICILLIN G + BENEMETHAMINE PENICILLIN & PROCAINE BENZYLPENICILLIN inj |
| 95592998 | PENICILLIN G intrathec inj 12mg/vial |
| 95593998 | PENICILLIN G inj 6g/vial |
| 95594996 | PENICILLIN G inj 3g/vial |
| 95594997 | PENICILLIN G inj 600mg/vial |
| 95594998 | PENICILLIN G inj 300mg/vial |
| 95604990 | FLUCLOXACILLIN elixir 250mg/5ml |
| 95618990 | CO-AMOXICLAV tabs 500mg+125mg |
| 95719992 | V-CIL-K 500 MG PUL |
| 95801990 | CO-FLUAMPICIL caps 250mg+250mg |
| 95810998 | MEZLOCILLIN inf 5g/vial |
| 95811996 | MEZLOCILLIN inj 2g/vial |
| 95811997 | MEZLOCILLIN inj 1g/vial |
| 95811998 | MEZLOCILLIN inj 500mg/vial |
| 95842998 | METICILLIN SODIUM inj 1g/vial |
| 95843998 | METICILLIN SODIUM inj 1g/vial |
| 95872990 | CO-AMOXICLAV tabs 250mg+125mg |
| 95883992 | CARBENICILLIN 1 INJ |
| 95891992 | CLOXACILLIN TAB |
| 95921997 | MECILLINAM inj 400mg/vial |
| 95921998 | MECILLINAM inj 200mg/vial |
| 95976996 | BENZATHINE PENICILLIN inj 229mg/ml |
| 95976997 | BENZATHINE PENICILLIN drops 115mg/ml |
| 95976998 | BENZATHINE PENICILLIN susp 229mg/5ml |
| 96092992 | AMOXYCILLIN FIZTAB 125 MG TAB |
| 96137990 | CO-AMOXICLAV tabs 250mg+125mg |
| 96156990 | AMOXICILLIN sf pwdr 3g |
| 96183990 | CO-FLUAMPICIL caps 250mg+250mg |
| 96185998 | AMPICILLIN + SULBACTAM inj 500mg + 250mg |
| 96189990 | CO-AMOXICLAV tabs 250mg+125mg |
| 96239996 | AMOXICILLIN syrp 125mg/5ml |
| 96239997 | AMOXICILLIN caps 500mg |
| 96239998 | AMOXICILLIN caps 250mg |
| 96246992 | FLUCLOXACILLIN 125 MG POW |
| 96255998 | AMOXICILLIN syrp 250mg/5ml |
| 96256996 | AMOXICILLIN sf chewable tab 500mg |
| 96256997 | AMOXICILLIN sf chewable tab 250mg |
| 96256998 | AMOXICILLIN sf chewable tab 125mg |
| 96306990 | AMOXICILLIN syrp 250mg/5ml |
| 96307988 | AMOXICILLIN syrp 125mg/5ml |
| 96307989 | AMOXICILLIN caps 500mg |
| 96307990 | AMOXICILLIN caps 250mg |
| 96309997 | AMOXICILLIN sf chewable tab 500mg |
| 96309998 | AMOXICILLIN sf chewable tab 250mg |
| 96313996 | AMOXICILLIN syrp 125mg/5ml |
| 96313997 | AMOXICILLIN caps 500mg |
| 96313998 | AMOXICILLIN caps 250mg |
| 96343990 | CO-AMOXICLAV tabs 250mg+125mg |
| 96365998 | AMOXICILLIN syrp 250mg/5ml |
| 96371989 | FLUCLOXACILLIN caps 500mg |
| 96371990 | FLUCLOXACILLIN caps 250mg |
| 96377990 | AMOXICILLIN syrp 250mg/5ml |
| 96383988 | AMOXICILLIN syrp 125mg/5ml |
| 96383989 | AMOXICILLIN caps 500mg |
| 96383990 | AMOXICILLIN caps 250mg |
| 96390992 | PHENOXYMETHYLPENICILLIN SACHETS 125 MG GRA |
| 96391990 | CO-AMOXICLAV tabs 500mg+125mg |
| 96391992 | PHENOXYMETHYLPENICILLIN 150 MG SYR |
| 96421992 | PYOPEN 250 MG INJ |
| 96428988 | FLUCLOXACILLIN elixir 125mg/5ml |
| 96428989 | FLUCLOXACILLIN caps 500mg |
| 96428990 | FLUCLOXACILLIN caps 250mg |
| 96457990 | AMPICILLIN susp 250mg/5ml |
| 96467992 | STABILLIN V-K 125 MG SYR |
| 96477992 | TALPEN 250 MG SYR |
| 96478992 | TALAMPICILLIN 250 MG SYR |
| 96529996 | FLUCLOXACILLIN pwdr/inj.soln 500mg/vial |
| 96529997 | FLUCLOXACILLIN caps 500mg |
| 96529998 | FLUCLOXACILLIN caps 250mg |
| 96530998 | FLUCLOXACILLIN + AMPICILLIN inj 500mg+500mg |
| 96531996 | FLUCLOXACILLIN + AMPICILLIN inj 250mg+250mg |
| 96531997 | FLUCLOXACILLIN + AMPICILLIN syrp 125mg+125mg |
| 96531998 | FLUCLOXACILLIN + AMPICILLIN caps 250mg+250mg |
| 96532997 | FLUCLOXACILLIN pwdr/inj.soln 1g/vial |
| 96532998 | FLUCLOXACILLIN IV inj 500mg/vial |
| 96533996 | FLUCLOXACILLIN IV inj 250mg/vial |
| 96533997 | FLUCLOXACILLIN pwdr/inj.soln 500mg/vial |
| 96533998 | FLUCLOXACILLIN pwdr/inj.soln 250mg/vial |
| 96538990 | CO-AMOXICLAV sf susp 250mg + 62mg/5ml |
| 96539988 | CO-AMOXICLAV sf susp 125mg + 31mg/5ml |
| 96539989 | CO-AMOXICLAV tabs 500mg+125mg |
| 96539990 | CO-AMOXICLAV tabs 250mg+125mg |
| 96547990 | AMPICILLIN caps 250mg |
| 96554989 | PHENOXYMETHYLPENICILLIN oral soln 250mg/5ml |
| 96554990 | PHENOXYMETHYLPENICILLIN oral soln 125mg/5ml |
| 96555988 | AMPICILLIN caps 500mg |
| 96555989 | AMPICILLIN susp 250mg/5ml |
| 96555990 | AMPICILLIN susp 125mg/5ml |
| 96556990 | PHENOXYMETHYLPENICILLIN tabs 250mg |
| 96582992 | AMPICILLIN 125 MG TAB |
| 96609992 | CARFECILLIN SODIUM 500 MG TAB |
| 96617992 | CLOXACILLIN 250 MG INJ |
| 96619997 | CLOXACILLIN IV inj 1g/vial |
| 96619998 | CLOXACILLIN IV inj 500mg/vial |
| 96620996 | CLOXACILLIN IV inj 250mg/vial |
| 96620997 | CLOXACILLIN IM inj 500mg/vial |
| 96620998 | CLOXACILLIN IM inj 250mg/vial |
| 96621996 | CLOXACILLIN syrp 125mg/5ml |
| 96621997 | CLOXACILLIN caps 500mg |
| 96621998 | CLOXACILLIN caps 250mg |
| 96637989 | CO-AMOXICLAV tabs 500mg+125mg |
| 96637990 | CO-AMOXICLAV tabs 250mg+125mg |
| 96659992 | FLUCLOXACILLIN 1 GM CAP |
| 96660996 | CLAVULANIC ACID + AMOXICILLIN sf susp 31mg + 125mg/5ml |
| 96660997 | CLAVULANIC ACID + AMOXICILLIN disp tab |
| 96660998 | CLAVULANIC ACID + AMOXICILLIN tabs 125mg + 250mg |
| 96665998 | CICLACILLIN susp 250mg/5ml |
| 96666996 | CICLACILLIN susp 125mg/5ml |
| 96666997 | CICLACILLIN tabs 500mg |
| 96666998 | CICLACILLIN tabs 250mg |
| 96716990 | AMPICILLIN susp 250mg/5ml |
| 96717988 | AMPICILLIN susp 125mg/5ml |
| 96717989 | AMPICILLIN caps 500mg |
| 96717990 | AMPICILLIN caps 250mg |
| 96731988 | CO-AMOXICLAV sf susp 250mg + 62mg/5ml |
| 96731989 | CO-AMOXICLAV sf susp 125mg + 31mg/5ml |
| 96731990 | CO-AMOXICLAV tabs 250mg+125mg |
| 96757988 | PHENOXYMETHYLPENICILLIN oral soln 250mg/5ml |
| 96757989 | PHENOXYMETHYLPENICILLIN oral soln 125mg/5ml |
| 96757990 | PHENOXYMETHYLPENICILLIN tabs 250mg |
| 96778992 | SULTAMICILLIN TOSYLATE 375 MG TAB |
| 96780997 | FLUCLOXACILLIN caps 500mg |
| 96780998 | FLUCLOXACILLIN caps 250mg |
| 96808988 | FLUCLOXACILLIN pwdr/inj.soln 500mg/vial |
| 96808989 | FLUCLOXACILLIN pwdr/inj.soln 250mg/vial |
| 96808990 | FLUCLOXACILLIN pwdr/inj.soln 1g/vial |
| 96814990 | AMPICILLIN susp 250mg/5ml |
| 96815990 | AMPICILLIN susp 125mg/5ml |
| 96822992 | AMOXYCILLIN FIZTAB 250 MG TAB |
| 96865988 | FLUCLOXACILLIN elixir 125mg/5ml |
| 96865989 | FLUCLOXACILLIN caps 500mg |
| 96865990 | FLUCLOXACILLIN caps 250mg |
| 96865998 | CARFECILLIN tabs 500mg |
| 96879997 | CARBENICILLIN sterile pwdr 5g |
| 96879998 | CARBENICILLIN sterile pwdr 1g |
| 96880997 | CARBENICILLIN sterile pwdr 5g |
| 96880998 | CARBENICILLIN sterile pwdr 1g |
| 96896990 | CO-FLUAMPICIL caps 250mg+250mg |
| 96897990 | FLUCLOXACILLIN elixir 250mg/5ml |
| 96908990 | CO-FLUAMPICIL caps 250mg+250mg |
| 96918998 | BACAMPICILLIN HCl tabs 400mg |
| 96919998 | BACAMPICILLIN HCl tabs 400mg |
| 96926989 | AMPICILLIN caps 500mg |
| 96926990 | AMPICILLIN caps 250mg |
| 96928988 | AMOXICILLIN sf oral susp 250mg/5ml |
| 96928989 | AMOXICILLIN sf oral susp 125mg/5ml |
| 96928990 | AMOXICILLIN syrp 250mg/5ml |
| 96930996 | AZLOCILLIN inj 5g/vial |
| 96930997 | AZLOCILLIN inj 1g/vial |
| 96930998 | AZLOCILLIN inj 500mg/vial |
| 96931988 | AMOXICILLIN syrp 125mg/5ml |
| 96931989 | AMOXICILLIN caps 500mg |
| 96931990 | AMOXICILLIN caps 250mg |
| 96931996 | AZLOCILLIN inj 5g/vial |
| 96931997 | AZLOCILLIN inj 1g/vial |
| 96931998 | AZLOCILLIN inj 500mg/vial |
| 96942989 | FLUCLOXACILLIN caps 500mg |
| 96942990 | FLUCLOXACILLIN caps 250mg |
| 96943996 | AMOXICILLIN inj 500mg |
| 96943997 | AMOXICILLIN inj 250mg |
| 96943998 | AMOXICILLIN sf pwdr 3g |
| 96944992 | AMPICILLIN 125 MG CAP |
| 96944996 | AMOXICILLIN sf pwdr 750mg |
| 96944997 | AMOXICILLIN paed susp 125mg/1.25ml |
| 96944998 | AMOXICILLIN disp tab 500mg |
| 96965990 | AMOXICILLIN syrp 250mg/5ml |
| 96974990 | CO-AMOXICLAV sf susp 250mg + 62mg/5ml |
| 96975988 | CO-AMOXICLAV sf susp 125mg + 31mg/5ml |
| 96975989 | CO-AMOXICLAV tabs 500mg+125mg |
| 96975990 | CO-AMOXICLAV tabs 250mg+125mg |
| 96990989 | AMOXICILLIN caps 500mg |
| 96990990 | AMOXICILLIN caps 250mg |
| 97009990 | CO-AMOXICLAV sf susp 250mg + 62mg/5ml |
| 97010988 | CO-AMOXICLAV sf susp 125mg + 31mg/5ml |
| 97010989 | CO-AMOXICLAV tabs 500mg+125mg |
| 97010990 | CO-AMOXICLAV tabs 250mg+125mg |
| 97036990 | FLUCLOXACILLIN elixir 250mg/5ml |
| 97047990 | AMOXICILLIN inj 500mg |
| 97050989 | AMOXICILLIN caps 500mg |
| 97050990 | AMOXICILLIN caps 250mg |
| 97090997 | PHENOXYMETHYLPENICILLIN tabs 250mg |
| 97090998 | PHENOXYMETHYLPENICILLIN tabs 125mg |
| 97110997 | PENICILLIN V tabs 250mg |
| 97110998 | PENICILLIN V tabs 125mg |
| 97115998 | FLUCLOXACILLIN elixir 250mg/5ml |
| 97116996 | FLUCLOXACILLIN elixir 125mg/5ml |
| 97116997 | FLUCLOXACILLIN caps 500mg |
| 97116998 | FLUCLOXACILLIN caps 250mg |
| 97118989 | FLUCLOXACILLIN caps 500mg |
| 97118990 | FLUCLOXACILLIN caps 250mg |
| 97129996 | AMPICILLIN sf susp 250mg/5ml |
| 97129997 | AMPICILLIN sf susp 125mg/5ml |
| 97129998 | AMPICILLIN susp 250mg/5ml |
| 97130996 | AMPICILLIN susp 125mg/5ml |
| 97130997 | AMPICILLIN caps 500mg |
| 97130998 | AMPICILLIN caps 250mg |
| 97131996 | AMOXICILLIN sf chewable tab 125mg |
| 97131997 | AMOXICILLIN caps 500mg |
| 97131998 | AMOXICILLIN caps 250mg |
| 97132989 | AMPICILLIN caps 500mg |
| 97132990 | AMPICILLIN caps 250mg |
| 97132997 | AMPICILLIN caps 500mg |
| 97132998 | AMPICILLIN caps 250mg |
| 97133988 | AMOXICILLIN sf oral susp 250mg/5ml |
| 97133989 | AMOXICILLIN sf oral susp 125mg/5ml |
| 97133990 | AMOXICILLIN caps 250mg |
| 97133998 | CO-FLUAMPICIL caps 250mg+250mg |
| 97159998 | PHENOXYMETHYLPENICILLIN oral soln 250mg/5ml |
| 97167990 | CO-FLUAMPICIL caps 250mg+250mg |
| 97172992 | CO-CAPS PENICILLIN V-K 250 MG CAP |
| 97208990 | PHENOXYMETHYLPENICILLIN tabs 250mg |
| 97241992 | DEDOXIL 500 MG CAP |
| 97242988 | PHENOXYMETHYLPENICILLIN oral soln 250mg/5ml |
| 97242989 | PHENOXYMETHYLPENICILLIN oral soln 125mg/5ml |
| 97242990 | PHENOXYMETHYLPENICILLIN tabs 250mg |
| 97242992 | DEDOXIL 250 MG CAP |
| 97245996 | TICARCILLIN + CLAVULANIC ACID inf 750mg + 50mg |
| 97245997 | TICARCILLIN + CLAVULANIC ACID inf 1.5g + 100mg |
| 97245998 | TICARCILLIN + CLAVULANIC ACID pow conc sol inf 3g + 200mg |
| 97254998 | CO-FLUAMPICIL syrp 125mg+125mg |
| 97260989 | FLUCLOXACILLIN elixir 250mg/5ml |
| 97260990 | FLUCLOXACILLIN susp 125mg/5ml |
| 97266998 | BENZATHINE PENICILLIN inj 229mg/ml |
| 97290997 | BENZATHINE PENICILLIN drops 115mg/ml |
| 97290998 | BENZATHINE PENICILLIN susp 229mg/5ml |
| 97309996 | MEZLOCILLIN inj 2g/vial |
| 97309997 | MEZLOCILLIN inj 1g/vial |
| 97309998 | MEZLOCILLIN inj 500mg/vial |
| 97432997 | FLUCLOXACILLIN susp 250mg/5ml |
| 97432998 | FLUCLOXACILLIN susp 125mg/5ml |
| 97536989 | PHENOXYMETHYLPENICILLIN oral soln 250mg/5ml |
| 97536990 | PHENOXYMETHYLPENICILLIN oral soln 125mg/5ml |
| 97553990 | FLUCLOXACILLIN elixir 125mg/5ml |
| 97591997 | PHENOXYMETHYLPENICILLIN oral soln 250mg/5ml |
| 97591998 | PHENOXYMETHYLPENICILLIN oral soln 125mg/5ml |
| 97592998 | PHENOXYMETHYLPENICILLIN tabs 250mg |
| 97614998 | BENZYLPENICILLIN intrathec inj 12mg/vial |
| 97615996 | BENZYLPENICILLIN inj 300mg/vial |
| 97615997 | BENZYLPENICILLIN inj 6g/vial |
| 97615998 | BENZYLPENICILLIN inj 3g/vial |
| 97616997 | BENZYLPENICILLIN pwdr/inj.soln 1200mg |
| 97616998 | BENZYLPENICILLIN pwdr/inj.soln 600mg/vial |
| 97646992 | LEDERCILLIN 250 MG TAB |
| 97656989 | PHENOXYMETHYLPENICILLIN oral soln 250mg/5ml |
| 97656990 | PHENOXYMETHYLPENICILLIN oral soln 125mg/5ml |
| 97701998 | PHENOXYMETHYLPENICILLIN tabs 250mg |
| 97708996 | CLAVULANIC ACID + AMOXICILLIN sf susp 57mg + 400mg/5ml |
| 97708997 | CLAVULANIC ACID + AMOXICILLIN tabs 125mg+500mg |
| 97708998 | CLAVULANIC ACID + AMOXICILLIN sf susp 62mg + 250mg/5ml |
| 97754988 | FLUCLOXACILLIN elixir 125mg/5ml |
| 97754989 | FLUCLOXACILLIN caps 500mg |
| 97754990 | FLUCLOXACILLIN caps 250mg |
| 97770990 | CO-FLUAMPICIL caps 250mg+250mg |
| 97776996 | AMPICILLIN susp 125mg/5ml |
| 97776997 | AMPICILLIN caps 500mg |
| 97776998 | AMPICILLIN caps 250mg |
| 97787988 | AMPICILLIN caps 500mg |
| 97787989 | AMPICILLIN caps 250mg |
| 97787990 | AMPICILLIN susp 250mg/5ml |
| 97788988 | AMPICILLIN susp 125mg/5ml |
| 97788989 | AMPICILLIN caps 500mg |
| 97788990 | AMPICILLIN caps 250mg |
| 97789990 | AMOXICILLIN sf oral susp 250mg/5ml |
| 97790988 | AMOXICILLIN sf oral susp 125mg/5ml |
| 97790989 | AMOXICILLIN sf pwdr 3g |
| 97790990 | AMOXICILLIN caps 250mg |
| 97792997 | CO-AMOXICLAV inj 1000mg+200mg |
| 97792998 | CO-AMOXICLAV inj 500mg+100mg |
| 97802998 | AMPICILLIN susp 250mg/5ml |
| 97808997 | AMOXICILLIN syrp 125mg/5ml |
| 97808998 | AMOXICILLIN caps 250mg |
| 97832992 | ORBENIN 500 MG INJ |
| 97833992 | ORBENIN 250 MG INJ |
| 97868996 | AMOXICILLIN sf pwdr 750mg |
| 97868997 | AMOXICILLIN sf oral susp 250mg/5ml |
| 97868998 | AMOXICILLIN sf oral susp 125mg/5ml |
| 97871992 | PENAMECILLIN 350 MG TAB |
| 97880989 | PHENOXYMETHYLPENICILLIN oral soln 250mg/5ml |
| 97880990 | PHENOXYMETHYLPENICILLIN oral soln 125mg/5ml |
| 97881992 | PHENETHICILLIN POTASSIUM 250 MG TAB |
| 97881997 | FLUCLOXACILLIN susp 250mg/5ml |
| 97881998 | FLUCLOXACILLIN susp 125mg/5ml |
| 97882998 | FLUCLOXACILLIN pwdr/inj.soln 1g/vial |
| 97883996 | FLUCLOXACILLIN pwdr/inj.soln 500mg/vial |
| 97883997 | FLUCLOXACILLIN pwdr/inj.soln 250mg/vial |
| 97883998 | FLUCLOXACILLIN pwdr/inj.soln 500mg/vial |
| 97884996 | FLUCLOXACILLIN pwdr/inj.soln 250mg/vial |
| 97884997 | FLUCLOXACILLIN caps 500mg |
| 97884998 | FLUCLOXACILLIN caps 250mg |
| 97889996 | PIPERACILLIN inf 4g/infusion bottle |
| 97889997 | PIPERACILLIN inj 2g/vial |
| 97889998 | PIPERACILLIN inj 1g/vial |
| 97890992 | PHENOXYMETHYLPENICILLIN 125 MG SYR |
| 97891992 | PHENOXYMETHYLPENICILLIN 125 MG CAP |
| 97918992 | PONDOCILLIN sach 175 MG |
| 97956990 | AMOXICILLIN sf pwdr 3g |
| 97969992 | PENICILLIN V 500 MG CAP |
| 97970992 | PENICILLIN 500 MG TAB |
| 97975990 | CO-FLUAMPICIL caps 250mg+250mg |
| 98061990 | PHENOXYMETHYLPENICILLIN tabs 250mg |
| 98089997 | AZLOCILLIN inf 5g/vial |
| 98089998 | AZLOCILLIN inj 2g/vial |
| 98113990 | PHENOXYMETHYLPENICILLIN tabs 250mg |
| 98114989 | PHENOXYMETHYLPENICILLIN oral soln 250mg/5ml |
| 98114990 | PHENOXYMETHYLPENICILLIN oral soln 125mg/5ml |
| 98168997 | FLUCLOXACILLIN caps 500mg |
| 98168998 | FLUCLOXACILLIN caps 250mg |
| 98218990 | AMPICILLIN caps 500mg |
| 98218998 | AMPICILLIN + CLOXACILLIN inj 50mg + 25mg/vial |
| 98334988 | AMOXICILLIN sf oral susp 250mg/5ml |
| 98334989 | AMOXICILLIN sf oral susp 125mg/5ml |
| 98334990 | AMOXICILLIN caps 500mg |
| 98334998 | MEZLOCILLIN inf 5g/vial |
| 98353996 | AMOXICILLIN inj 1g |
| 98353997 | AMOXICILLIN inj 500mg |
| 98353998 | AMOXICILLIN inj 250mg |
| 98354988 | FLUCLOXACILLIN elixir 125mg/5ml |
| 98354989 | FLUCLOXACILLIN caps 500mg |
| 98354990 | FLUCLOXACILLIN caps 250mg |
| 98354998 | AMOXICILLIN paed susp 125mg/1.25ml |
| 98355998 | TALAMPICILLIN syrp 125mg/5ml |
| 98356997 | AMPICILLIN inj 500mg |
| 98356998 | AMPICILLIN inj 250mg |
| 98357998 | AMPICILLIN paed susp 125mg/1.25ml |
| 98362997 | CO-FLUAMPICIL inj 500mg+500mg |
| 98362998 | CO-FLUAMPICIL inj 250mg+250mg |
| 98363998 | CO-AMOXICLAV sf susp 125mg + 31mg/5ml |
| 98364998 | CO-AMOXICLAV sf susp 250mg + 62mg/5ml |
| 98382998 | PHENOXYMETHYLPENICILLIN oral soln 125mg/5ml |
| 98383998 | PHENOXYMETHYLPENICILLIN oral soln 125mg/5ml |
| 98435990 | PHENOXYMETHYLPENICILLIN tabs 250mg |
| 98450998 | PIVMECILLINAM + PIVAMPICILLIN susp 46.2mg + 62.5mg/5ml |
| 98451998 | PIVMECILLINAM + PIVAMPICILLIN tabs 100mg + 125mg |
| 98552989 | FLUCLOXACILLIN caps 500mg |
| 98552990 | FLUCLOXACILLIN caps 250mg |
| 98566988 | AMPICILLIN caps 250mg |
| 98566989 | AMPICILLIN susp 250mg/5ml |
| 98566990 | AMPICILLIN susp 125mg/5ml |
| 98595990 | AMPICILLIN susp 250mg/5ml |
| 98622990 | FLUCLOXACILLIN pwdr/inj.soln 500mg/vial |
| 98627988 | FLUCLOXACILLIN pwdr/inj.soln 250mg/vial |
| 98627989 | FLUCLOXACILLIN caps 500mg |
| 98627990 | FLUCLOXACILLIN caps 250mg |
| 98673998 | AMOXICILLIN sf pwdr 3g |
| 98737990 | PHENOXYMETHYLPENICILLIN tabs 250mg |
| 98741996 | FLUCLOXACILLIN pwdr/inj.soln 250mg/vial |
| 98741997 | FLUCLOXACILLIN caps 500mg |
| 98741998 | FLUCLOXACILLIN caps 250mg |
| 98778988 | AMPICILLIN inj 500mg |
| 98778989 | AMPICILLIN susp 250mg/5ml |
| 98778990 | AMPICILLIN susp 125mg/5ml |
| 98779989 | AMOXICILLIN inj 250mg |
| 98779990 | AMOXICILLIN syrp 250mg/5ml |
| 98802988 | AMPICILLIN inj 250mg |
| 98802989 | AMPICILLIN caps 500mg |
| 98802990 | AMPICILLIN caps 250mg |
| 98803988 | AMOXICILLIN syrp 125mg/5ml |
| 98803989 | AMOXICILLIN caps 500mg |
| 98803990 | AMOXICILLIN caps 250mg |
| 98820997 | PIVAMPICILLIN sach 175mg |
| 98820998 | PIVAMPICILLIN susp 175mg/5ml |
| 98823998 | PIVMECILLINAM tabs 200mg |
| 98825989 | PHENOXYMETHYLPENICILLIN oral soln 250mg/5ml |
| 98825990 | PHENOXYMETHYLPENICILLIN oral soln 125mg/5ml |
| 98826989 | PHENOXYMETHYLPENICILLIN oral soln 250mg/5ml |
| 98826990 | PHENOXYMETHYLPENICILLIN oral soln 125mg/5ml |
| 98827989 | PHENOXYMETHYLPENICILLIN oral soln 250mg/5ml |
| 98827990 | PHENOXYMETHYLPENICILLIN oral soln 125mg/5ml |
| 98883997 | AMPICILLIN inj 500mg |
| 98883998 | AMPICILLIN inj 250mg |
| 98884998 | AMPICILLIN susp 250mg/5ml |
| 98885998 | AMPICILLIN susp 125mg/5ml |
| 98967998 | AMPICILLIN susp 250mg/5ml |
| 98968996 | AMPICILLIN susp 125mg/5ml |
| 98968997 | AMPICILLIN caps 500mg |
| 98968998 | AMPICILLIN caps 250mg |
| 98986996 | PHENOXYMETHYLPENICILLIN oral soln 250mg/5ml |
| 98986997 | PHENOXYMETHYLPENICILLIN oral soln 125mg/5ml |
| 98986998 | PHENOXYMETHYLPENICILLIN syrp 62.5mg/5ml |
| 98987996 | PHENOXYMETHYLPENICILLIN tabs 250mg |
| 98987997 | PHENOXYMETHYLPENICILLIN tabs 125mg |
| 98987998 | PHENOXYMETHYLPENICILLIN caps 250mg |
| 99001998 | CARFECILLIN tabs 500mg |
| 99018998 | BENZYLPENICILLIN SODIUM + BENEMETHAMINE PENICILLIN & PROCAINE BENZYLPENICILLIN inj |
| 99075998 | TALAMPICILLIN tabs 250mg |
| 99110998 | CO-AMOXICLAV tabs 500mg+125mg |
| 99111997 | AMOXICILLIN + CLAVULANIC ACID sf susp 400mg + 57mg/5ml |
| 99111998 | AMOXICILLIN + CLAVULANIC ACID tabs 500mg+125mg |
| 99146997 | MECILLINAM inj 400mg/vial |
| 99146998 | MECILLINAM inj 200mg/vial |
| 99147998 | PIVMECILLINAM susp 100mg/sachet |
| 99171996 | AMPICILLIN + CLOXACILLIN inj 250mg + 250mg/vial |
| 99171997 | AMPICILLIN + CLOXACILLIN syrp 250mg/5ml |
| 99171998 | AMPICILLIN + CLOXACILLIN caps 500mg |
| 99236998 | PIVAMPICILLIN tabs 500mg |
| 99274998 | AMPICILLIN susp 250mg/5ml |
| 99275996 | AMPICILLIN susp 125mg/5ml |
| 99275997 | AMPICILLIN caps 500mg |
| 99275998 | AMPICILLIN caps 250mg |
| 99311998 | CLOXACILLIN IV inj 500mg/vial |
| 99312996 | CLOXACILLIN IV inj 250mg/vial |
| 99312997 | CLOXACILLIN IM inj 500mg/vial |
| 99312998 | CLOXACILLIN IM inj 250mg/vial |
| 99313996 | CLOXACILLIN syrp 125mg/5ml |
| 99313997 | CLOXACILLIN caps 500mg |
| 99313998 | CLOXACILLIN caps 250mg |
| 99452998 | CO-FLUAMPICIL caps 250mg+250mg |
| 99465990 | PHENOXYMETHYLPENICILLIN tabs 250mg |
| 99466990 | PHENOXYMETHYLPENICILLIN tabs 250mg |
| 99467990 | PHENOXYMETHYLPENICILLIN tabs 250mg |
| 99468990 | PHENOXYMETHYLPENICILLIN tabs 250mg |
| 99469989 | PHENOXYMETHYLPENICILLIN oral soln 250mg/5ml |
| 99469990 | PHENOXYMETHYLPENICILLIN oral soln 125mg/5ml |
| 99590988 | FLUCLOXACILLIN elixir 125mg/5ml |
| 99590989 | FLUCLOXACILLIN caps 500mg |
| 99590990 | FLUCLOXACILLIN caps 250mg |
| 99591989 | FLUCLOXACILLIN caps 500mg |
| 99591990 | FLUCLOXACILLIN caps 250mg |
| 99592988 | FLUCLOXACILLIN elixir 125mg/5ml |
| 99592989 | FLUCLOXACILLIN caps 500mg |
| 99592990 | FLUCLOXACILLIN caps 250mg |
| 99736997 | PHENOXYMETHYLPENICILLIN syrp 62.5mg/5ml |
| 99736998 | PHENOXYMETHYLPENICILLIN oral soln 250mg/5ml |
| 99737996 | PHENOXYMETHYLPENICILLIN oral soln 125mg/5ml |
| 99737997 | PHENOXYMETHYLPENICILLIN tabs 250mg |
| 99737998 | PHENOXYMETHYLPENICILLIN tabs 125mg |
| 99743992 | AMOXYCILLIN 500 MG TAB |
| 99759992 | APSIN VK 125 MG SYR |
| 99774998 | PROCAINE BENZYLPENICILLIN inj |
| 99789992 | AMPIMED 250 MG CAP |
| 99792992 | AMPLICLOX pwdr FOR syrp 250 MG/5ML POW |
| 99793992 | AMPICLOX MG INJ |
| 99800996 | BENZYLPENICILLIN syrp 250mg/5ml |
| 99800997 | BENZYLPENICILLIN syrp 125mg/5ml |
| 99800998 | BENZYLPENICILLIN tabs 250mg |
| 99821988 | AMPICILLIN sf susp 250mg/5ml |
| 99821989 | AMPICILLIN sf susp 125mg/5ml |
| 99821990 | AMPICILLIN susp 250mg/5ml |
| 99822988 | AMPICILLIN susp 125mg/5ml |
| 99822989 | AMPICILLIN caps 500mg |
| 99822990 | AMPICILLIN caps 250mg |
| 99823990 | AMPICILLIN susp 250mg/5ml |
| 99824988 | AMPICILLIN susp 125mg/5ml |
| 99824989 | AMPICILLIN caps 500mg |
| 99824990 | AMPICILLIN caps 250mg |
| 99825990 | AMPICILLIN susp 250mg/5ml |
| 99826988 | AMPICILLIN susp 125mg/5ml |
| 99826989 | AMPICILLIN caps 500mg |
| 99826990 | AMPICILLIN caps 250mg |
| 99827990 | AMOXICILLIN syrp 250mg/5ml |
| 99828990 | AMOXICILLIN sf oral susp 250mg/5ml |
| 99829990 | AMOXICILLIN syrp 250mg/5ml |
| 99830988 | AMOXICILLIN sf pwdr 125mg |
| 99830989 | AMOXICILLIN sf pwdr 3g |
| 99830990 | AMOXICILLIN syrp 250mg/5ml |
| 99831988 | AMOXICILLIN sf oral susp 250mg/5ml |
| 99831989 | AMOXICILLIN sf oral susp 125mg/5ml |
| 99831990 | AMOXICILLIN syrp 250mg/5ml |
| 99832988 | AMOXICILLIN sf oral susp 250mg/5ml |
| 99832989 | AMOXICILLIN sf oral susp 125mg/5ml |
| 99832990 | AMOXICILLIN syrp 250mg/5ml |
| 99833990 | AMOXICILLIN syrp 250mg/5ml |
| 99834990 | AMOXICILLIN syrp 250mg/5ml |
| 99836988 | AMOXICILLIN syrp 125mg/5ml |
| 99836989 | AMOXICILLIN caps 500mg |
| 99836990 | AMOXICILLIN caps 250mg |
| 99836992 | AMOXYCILLIN 125MG/62MG CLAVULANIC ACID SYR |
| 99837988 | AMOXICILLIN syrp 125mg/5ml |
| 99837989 | AMOXICILLIN caps 500mg |
| 99837990 | AMOXICILLIN caps 250mg |
| 99838988 | AMOXICILLIN sf oral susp 125mg/5ml |
| 99838989 | AMOXICILLIN caps 500mg |
| 99838990 | AMOXICILLIN caps 250mg |
| 99839988 | AMOXICILLIN syrp 125mg/5ml |
| 99839989 | AMOXICILLIN caps 500mg |
| 99839990 | AMOXICILLIN caps 250mg |
| 99840988 | AMOXICILLIN syrp 250mg/5ml |
| 99840989 | AMOXICILLIN syrp 125mg/5ml |
| 99840990 | AMOXICILLIN caps 500mg |
| 99841988 | AMOXICILLIN syrp 125mg/5ml |
| 99841989 | AMOXICILLIN caps 500mg |
| 99841990 | AMOXICILLIN caps 250mg |
| 99842988 | AMOXICILLIN syrp 125mg/5ml |
| 99842989 | AMOXICILLIN caps 500mg |
| 99842990 | AMOXICILLIN caps 250mg |
| 99843988 | AMOXICILLIN syrp 125mg/5ml |
| 99843989 | AMOXICILLIN caps 500mg |
| 99843990 | AMOXICILLIN caps 250mg |
| 99844988 | AMOXICILLIN syrp 125mg/5ml |
| 99844989 | AMOXICILLIN caps 500mg |
| 99844990 | AMOXICILLIN caps 250mg |
| 99845988 | AMOXICILLIN syrp 125mg/5ml |
| 99845989 | AMOXICILLIN caps 500mg |
| 99845990 | AMOXICILLIN caps 250mg |
| 99846988 | AMOXICILLIN syrp 250mg/5ml |
| 99846989 | AMOXICILLIN syrp 125mg/5ml |
| 99846990 | AMOXICILLIN caps 500mg |
| 99847988 | AMOXICILLIN inj 250mg |
| 99847989 | AMOXICILLIN inj 1g |
| 99847990 | AMOXICILLIN syrp 250mg/5ml |
| 99848988 | AMOXICILLIN syrp 125mg/5ml |
| 99848989 | AMOXICILLIN caps 500mg |
| 99848990 | AMOXICILLIN caps 250mg |
| 99849988 | AMOXICILLIN caps 250mg |
| 99849989 | AMOXICILLIN syrp 250mg/5ml |
| 99849990 | AMOXICILLIN syrp 125mg/5ml |
| 99860998 | CICLACILLIN susp 250mg/5ml |
| 99861996 | CICLACILLIN susp 125mg/5ml |
| 99861997 | CICLACILLIN tabs 500mg |
| 99861998 | CICLACILLIN tabs 250mg |
| 99869997 | PHENETHICILLIN syrp 125mg/5ml |
| 99869998 | PHENETHICILLIN caps 250mg |
| 99886998 | PROCAINE BENZYLPENICILLIN + BENZYLPENICILLIN SODIUM inj |
| 99927997 | CO-AMOXICLAV disp tab 250mg+125mg |
| 99927998 | CO-AMOXICLAV tabs 250mg+125mg |
| 99960998 | AMPICILLIN + CLOXACILLIN sf susp 60mg + 30mg/0.6ml |
| 99962996 | AMOXICILLIN disp tab 500mg |
| 99962997 | AMOXICILLIN caps 500mg |
| 99962998 | AMOXICILLIN caps 250mg |
| 99964997 | AMPICILLIN caps 500mg |
| 99964998 | AMPICILLIN caps 250mg |
| 82286998 | MOXIFLOXACIN soln for inf 400mg/250ml |
| 83926998 | OFLOXACIN oral liq |
| 84062998 | CIPROFLOXACIN IN GLUCOSE 5% inf 400mg/200ml |
| 84063998 | CIPROFLOXACIN IN GLUCOSE 5% inf 200mg/100ml |
| 84350998 | CIPROFLOXACIN oral liq |
| 84614998 | MOXIFLOXACIN soln for inf 400mg/250ml |
| 84864998 | OFLOXACIN inf 200mg/100ml |
| 84865998 | OFLOXACIN inf 200mg/100ml |
| 87678998 | CIPROFLOXACIN IN SODIUM CHLORIDE 0.9% inf 400mg/200ml |
| 87679998 | CIPROFLOXACIN IN SODIUM CHLORIDE 0.9% inf 200mg/100ml |
| 87680998 | CIPROFLOXACIN IN SODIUM CHLORIDE 0.9% inf 100mg/50ml |
| 87681998 | CIPROFLOXACIN IN SODIUM CHLORIDE 0.9% inf 400mg/200ml |
| 87682998 | CIPROFLOXACIN IN SODIUM CHLORIDE 0.9% inf 200mg/100ml |
| 87683998 | CIPROFLOXACIN IN SODIUM CHLORIDE 0.9% inf 100mg/50ml |
| 88239997 | GREPAFLOXACIN tabs 600mg |
| 88239998 | GREPAFLOXACIN tabs 400mg |
| 88244997 | GREPAFLOXACIN tabs 600mg |
| 88244998 | GREPAFLOXACIN tabs 400mg |
| 88249998 | LEVOFLOXACIN iv inf 500mg |
| 88254998 | LEVOFLOXACIN iv inf 500mg |
| 88261997 | LEVOFLOXACIN FC tab 500mg |
| 88261998 | LEVOFLOXACIN FC tab 250mg |
| 88267997 | LEVOFLOXACIN FC tab 500mg |
| 88267998 | LEVOFLOXACIN FC tab 250mg |
| 89122998 | CIPROFLOXACIN IN SODIUM CHLORIDE 0.9% inf 2mg/ml |
| 89232998 | CIPROFLOXACIN IN GLUCOSE 5% inf 400mg/200ml |
| 89356998 | MOXIFLOXACIN tabs 400mg |
| 89361998 | MOXIFLOXACIN tabs 400mg |
| 91225998 | CIPROFLOXACIN IN GLUCOSE 5% inf 400mg/200ml |
| 91255997 | SPARFLOXACIN tabs 100mg |
| 91255998 | SPARFLOXACIN tabs 200mg |
| 91256997 | SPARFLOXACIN tabs 100mg |
| 91256998 | SPARFLOXACIN tabs 200mg |
| 92867990 | CIPROFLOXACIN IN GLUCOSE 5% inf 400mg/200ml |
| 92868990 | CIPROFLOXACIN IN GLUCOSE 5% inf 200mg/100ml |
| 93079996 | CIPROFLOXACIN susp 250mg/5ml |
| 93079997 | CIPROFLOXACIN tabs 100mg |
| 93079998 | CIPROFLOXACIN tabs 750mg |
| 93080996 | CIPROFLOXACIN susp 250mg/5ml |
| 93080997 | CIPROFLOXACIN tabs 100mg |
| 93080998 | CIPROFLOXACIN tabs 750mg |
| 93105990 | CIPROFLOXACIN IN SODIUM CHLORIDE 0.9% inf 400mg/200ml |
| 93106990 | CIPROFLOXACIN IN SODIUM CHLORIDE 0.9% inf 200mg/100ml |
| 93221990 | CIPROFLOXACIN IN SODIUM CHLORIDE 0.9% inf 400mg/200ml |
| 93222990 | CIPROFLOXACIN IN SODIUM CHLORIDE 0.9% inf 200mg/100ml |
| 93223990 | CIPROFLOXACIN IN SODIUM CHLORIDE 0.9% inf 100mg/50ml |
| 93255998 | NORFLOXACIN tabs 400mg |
| 93256998 | NORFLOXACIN tabs 400mg |
| 93552997 | OFLOXACIN tabs 400mg |
| 93552998 | OFLOXACIN tabs 200mg |
| 93554997 | OFLOXACIN tabs 400mg |
| 93554998 | OFLOXACIN tabs 200mg |
| 93757998 | NALIDIXIC ACID susp 300mg/5ml |
| 93830990 | CIPROFLOXACIN tabs 250mg |
| 94023990 | CIPROFLOXACIN tabs 500mg |
| 94024990 | CIPROFLOXACIN tabs 250mg |
| 94267990 | CIPROFLOXACIN tabs 750mg |
| 94268990 | CIPROFLOXACIN tabs 100mg |
| 94418990 | OFLOXACIN tabs 400mg |
| 94419990 | OFLOXACIN tabs 200mg |
| 94429998 | ENOXACIN tabs 200mg |
| 94430998 | ENOXACIN tabs 200mg |
| 94606990 | CIPROFLOXACIN tabs 500mg |
| 94607990 | CIPROFLOXACIN tabs 250mg |
| 94653990 | CIPROFLOXACIN tabs 750mg |
| 94691990 | CIPROFLOXACIN tabs 500mg |
| 94692990 | CIPROFLOXACIN tabs 250mg |
| 94912996 | CIPROFLOXACIN tabs 500mg |
| 94912997 | CIPROFLOXACIN IN SODIUM CHLORIDE 0.9% inf 2mg/ml |
| 94912998 | CIPROFLOXACIN tabs 250mg |
| 94913996 | CIPROFLOXACIN tabs 500mg |
| 94913997 | CIPROFLOXACIN inf 2mg/ml |
| 94913998 | CIPROFLOXACIN tabs 250mg |
| 95131990 | CIPROFLOXACIN tabs 500mg |
| 95132990 | CIPROFLOXACIN tabs 750mg |
| 95154990 | NORFLOXACIN tabs 400mg |
| 95181990 | CIPROFLOXACIN tabs 750mg |
| 95210990 | CIPROFLOXACIN tabs 750mg |
| 95211990 | CIPROFLOXACIN tabs 500mg |
| 95212990 | CIPROFLOXACIN tabs 250mg |
| 95266997 | TEMAFLOXACIN tabs 400mg |
| 95266998 | TEMAFLOXACIN tabs 300mg |
| 95274990 | OFLOXACIN tabs 400mg |
| 95275990 | OFLOXACIN tabs 200mg |
| 95305997 | TEMAFLOXACIN tabs 400mg |
| 95305998 | TEMAFLOXACIN tabs 300mg |
| 95344990 | OFLOXACIN tabs 400mg |
| 95355998 | ROSOXACIN caps 150mg |
| 95356998 | ROSOXACIN caps 150mg |
| 95366990 | NORFLOXACIN tabs 400mg |
| 95429990 | CIPROFLOXACIN tabs 500mg |
| 95430990 | CIPROFLOXACIN tabs 250mg |
| 95447990 | OFLOXACIN tabs 200mg |
| 95627990 | CIPROFLOXACIN tabs 750mg |
| 95628990 | CIPROFLOXACIN tabs 500mg |
| 95629990 | CIPROFLOXACIN tabs 250mg |
| 95686990 | CIPROFLOXACIN tabs 100mg |
| 95759998 | NALIDIXIC ACID + SODIUM CITRATE sach 660mg + 3750mg |
| 95760996 | NALIDIXIC ACID sf susp 300mg/5ml |
| 95760997 | NALIDIXIC ACID susp 300mg/5ml |
| 95760998 | NALIDIXIC ACID tabs 500mg |
| 95804990 | CIPROFLOXACIN tabs 100mg |
| 95821990 | CIPROFLOXACIN tabs 750mg |
| 95822990 | CIPROFLOXACIN tabs 500mg |
| 95823990 | CIPROFLOXACIN tabs 250mg |
| 95824990 | CIPROFLOXACIN tabs 100mg |
| 95865990 | CIPROFLOXACIN tabs 750mg |
| 95866990 | CIPROFLOXACIN tabs 500mg |
| 95867990 | CIPROFLOXACIN tabs 250mg |
| 95946990 | CIPROFLOXACIN tabs 750mg |
| 95947990 | CIPROFLOXACIN tabs 500mg |
| 95948990 | CIPROFLOXACIN tabs 250mg |
| 95949990 | CIPROFLOXACIN tabs 100mg |
| 95956990 | OFLOXACIN tabs 200mg |
| 95964990 | NORFLOXACIN tabs 400mg |
| 96022990 | CIPROFLOXACIN tabs 750mg |
| 96023990 | CIPROFLOXACIN tabs 500mg |
| 96024990 | CIPROFLOXACIN tabs 250mg |
| 96030990 | CIPROFLOXACIN tabs 750mg |
| 96031990 | CIPROFLOXACIN tabs 500mg |
| 96032990 | CIPROFLOXACIN tabs 250mg |
| 96033990 | CIPROFLOXACIN tabs 100mg |
| 96042990 | CIPROFLOXACIN tabs 750mg |
| 96043990 | CIPROFLOXACIN tabs 500mg |
| 96044990 | CIPROFLOXACIN tabs 250mg |
| 96045990 | CIPROFLOXACIN tabs 100mg |
| 96047990 | CIPROFLOXACIN tabs 750mg |
| 96048990 | CIPROFLOXACIN tabs 500mg |
| 96049990 | CIPROFLOXACIN tabs 250mg |
| 96050990 | CIPROFLOXACIN tabs 750mg |
| 96051990 | CIPROFLOXACIN tabs 500mg |
| 96052990 | CIPROFLOXACIN tabs 250mg |
| 96056990 | CIPROFLOXACIN tabs 750mg |
| 96057990 | CIPROFLOXACIN tabs 500mg |
| 96058990 | CIPROFLOXACIN tabs 250mg |
| 96066990 | CIPROFLOXACIN tabs 500mg |
| 96067990 | CIPROFLOXACIN tabs 250mg |
| 96099990 | OFLOXACIN tabs 400mg |
| 96100990 | OFLOXACIN tabs 200mg |
| 96185990 | OFLOXACIN tabs 400mg |
| 96186990 | OFLOXACIN tabs 400mg |
| 96187990 | OFLOXACIN tabs 200mg |
| 96188990 | OFLOXACIN tabs 200mg |
| 96661998 | CINOXACIN caps 500mg |
| 96705990 | NORFLOXACIN tabs 400mg |
| 97799998 | OFLOXACIN inf 2mg/ml |
| 97809998 | OFLOXACIN inf 2mg/ml |
| 98225992 | URIBEN 500 MG TAB |
| 98474998 | NALIDIXIC ACID + SODIUM CITRATE sach 660mg + 3750mg |
| 98478990 | NALIDIXIC ACID tabs 500mg |
| 99371997 | NALIDIXIC ACID sf susp 300mg/5ml |
| 99371998 | NALIDIXIC ACID tabs 500mg |
| 99826998 | CINOXACIN caps 500mg |
| 82529998 | RIFAXIMIN tabs 200mg |
| 84735998 | VANCOMYCIN oral liq |
| 85292998 | DAPTOMYCIN pwdr/inj.soln 500mg/vial |
| 85293998 | DAPTOMYCIN pwdr/inj.soln 500mg/vial |
| 85937998 | VANCOMYCIN pow conc sol inf 1g/vial |
| 85938998 | VANCOMYCIN pow conc sol inf 500mg/vial |
| 86166998 | DAPTOMYCIN pwdr/inj.soln 350mg/vial |
| 86167998 | DAPTOMYCIN pwdr/inj.soln 350mg/vial |
| 86181998 | VANCOMYCIN caps 125mg |
| 89025998 | LINEZOLID oral susp 100mg/5ml |
| 89354998 | COLISTIMETHATE SODIUM pdr for soln for neb 1 million units |
| 90390998 | CHLORAMPHENICOL INTRATHECAL (HUDDERSFIELD ROYAL INFIRMARY) inj 4mg/2ml |
| 91648998 | COLISTIMETHATE SODIUM pdr for soln for neb 1 million units |
| 91698998 | QUINUPRISTIN + DALFOPRISTIN pow conc sol inf 150mg + 350mg |
| 91882998 | COLISTIMETHATE SODIUM pdr/inj/neb soln 2 million units |
| 91885998 | COLISTIMETHATE SODIUM pdr/inj/neb soln 2 million units |
| 91886998 | COLISTIMETHATE SODIUM pwdr/inj.soln 1 million units |
| 91987998 | LINEZOLID oral susp 100mg/5ml |
| 92167998 | COLISTIMETHATE SODIUM pwdr/inj.soln 1 million units |
| 92483990 | VANCOMYCIN pow conc sol inf 500mg/vial |
| 92542990 | VANCOMYCIN pow conc sol inf 1g/vial |
| 92630998 | VANCOMYCIN pwdr 10g |
| 93260996 | VANCOMYCIN pow conc sol inf 1g/vial |
| 93260997 | VANCOMYCIN inj 250mg/vial |
| 93260998 | VANCOMYCIN pow conc sol inf 500mg/vial |
| 93603997 | TEICOPLANIN pwdr/inj.soln 400mg/vial |
| 93603998 | TEICOPLANIN pwdr/inj.soln 200mg/vial |
| 93604997 | TEICOPLANIN pwdr/inj.soln 400mg/vial |
| 93604998 | TEICOPLANIN pwdr/inj.soln 200mg/vial |
| 93829998 | FUSIDIC ACID inf |
| 94596990 | SODIUM FUSIDATE pow conc sol inf 500mg |
| 94790992 | CHLORAMPHENICOL POW |
| 94806992 | CHLOROMYCETIN POW |
| 95092997 | LINEZOLID inf 600mg |
| 95092998 | LINEZOLID tabs 600mg |
| 95147997 | LINEZOLID inf 600mg |
| 95147998 | LINEZOLID tabs 600mg |
| 95196990 | CHLORAMPHENICOL pwdr/inj.soln 1g/vial |
| 95262998 | SPECTINOMYCIN inj 2g/vial |
| 95263998 | SPECTINOMYCIN inj 2g/vial |
| 95291998 | SODIUM FUSIDATE tabs 250mg |
| 95475998 | POLYMYXIN B SULPHATE + NEOMYCIN & BACITRACIN irr soln |
| 95476998 | POLYMYXIN B SULPHATE inj 500000 units/vial |
| 95480998 | POLYMYXIN B SULPHATE + NEOMYCIN & BACITRACIN irr soln |
| 95481998 | POLYMYXIN B SULPHATE inj 500000 units/vial |
| 95565990 | VANCOMYCIN pow conc sol inf 1g/vial |
| 95573990 | VANCOMYCIN pow conc sol inf 500mg/vial |
| 95574990 | VANCOMYCIN caps 250mg |
| 95575990 | VANCOMYCIN caps 125mg |
| 95632996 | VANCOMYCIN pwdr 10g |
| 95632997 | VANCOMYCIN caps 250mg |
| 95632998 | VANCOMYCIN caps 125mg |
| 95633996 | VANCOMYCIN pow conc sol inf 1g/vial |
| 95633997 | VANCOMYCIN inj 250mg/vial |
| 95633998 | VANCOMYCIN pow conc sol inf 500mg/vial |
| 95634997 | VANCOMYCIN caps 250mg |
| 95634998 | VANCOMYCIN caps 125mg |
| 95773992 | VANCOMYCIN ORAL 10 GM SOL |
| 95774992 | VANCOCIN ORAL POW |
| 95846990 | CHLORAMPHENICOL INTRATHECAL (HUDDERSFIELD ROYAL INFIRMARY) inj 4mg/2ml |
| 95977992 | POLYMIXIN B SULPHATE INJ |
| 96153992 | CHLOROMYCETIN PALMITATE 125 MG SYR |
| 96256992 | FUCIDIN 500 MG TAB |
| 96466998 | FUSIDIC ACID susp 250mg/5ml |
| 96469998 | FUSIDIC ACID AS FUSIDATE SODIUM tabs 250mg |
| 96612997 | COLISTIMETHATE SODIUM pdr/inj/neb soln 0.5 million units |
| 96612998 | COLISTIMETHATE SODIUM pdr/inj/neb soln 1 million units |
| 96613997 | COLISTIN SULPHATE syrp 250000 units/5ml |
| 96613998 | COLISTIN SULPHATE tabs 1.5 million units |
| 96614997 | COLISTIMETHATE SODIUM pdr/inj/neb soln 1 million units |
| 96614998 | COLISTIMETHATE SODIUM pdr/inj/neb soln 0.5 million units |
| 96615997 | COLISTIN SULPHATE syrp 250000 units/5ml |
| 96615998 | COLISTIN SULPHATE tabs 1.5 million units |
| 96729998 | SODIUM FUSIDATE pow conc sol inf 500mg |
| 96737989 | VANCOMYCIN pow conc sol inf 1g/vial |
| 96737990 | VANCOMYCIN pow conc sol inf 500mg/vial |
| 96768992 | SODIUM FUSIDATE 500 MG TAB |
| 96769992 | SODIUM FUSIDATE 250 MG CAP |
| 96810998 | CHLORAMPHENICOL pwdr/inj.soln 1g/vial |
| 96811997 | CHLORAMPHENICOL sterile pwdr 5g/vial |
| 96811998 | CHLORAMPHENICOL sterile pwdr 1.2g/vial |
| 96812996 | CHLORAMPHENICOL sterile pwdr 300mg/vial |
| 96812997 | CHLORAMPHENICOL oral susp 125mg/ml |
| 96812998 | CHLORAMPHENICOL caps 250mg |
| 96815996 | CHLORAMPHENICOL pwdr/inj.soln 1g/vial |
| 96815997 | CHLORAMPHENICOL sterile pwdr 5g/vial |
| 96815998 | CHLORAMPHENICOL sterile pwdr 1.2g/vial |
| 96816996 | CHLORAMPHENICOL sterile pwdr 300mg/vial |
| 96816997 | CHLORAMPHENICOL oral susp 125mg/ml |
| 96816998 | CHLORAMPHENICOL caps 250mg |
| 97490992 | FUCIDIN EC 500 MG TAB |
| 97491992 | FUCIDIN POW |
| 97827990 | CHLORAMPHENICOL caps 250mg |
| 97841989 | VANCOMYCIN pow conc sol inf 1g/vial |
| 97841990 | VANCOMYCIN pow conc sol inf 500mg/vial |
| 97842989 | VANCOMYCIN caps 250mg |
| 97842990 | VANCOMYCIN caps 125mg |
| 97919990 | VANCOMYCIN pow conc sol inf 500mg/vial |
| 98086992 | SODIUM FUSIDATE POW |
| 98649998 | DALFOPRISTIN + QUINUPRISTIN pow conc sol inf 350mg + 150mg |
| 98762998 | DALFOPRISTIN + QUINUPRISTIN pow conc sol inf 350mg + 150mg |
| 98828998 | SODIUM FUSIDATE pow conc sol inf 500mg |
| 98829998 | FUSIDIC ACID susp 250mg/5ml |
| 99251988 | CHLORAMPHENICOL caps 250mg |
| 99619998 | SODIUM FUSIDATE tabs 250mg |
| 83768998 | SULFADIAZINE oral liq |
| 83782998 | TRIMETHOPRIM oral liq |
| 84563998 | TRIMETHOPRIM susp 50mg/5ml |
| 87034998 | TRIMETHOPRIM + SULFAMETHOXAZOLE conc soln inf 160mg + 800mg/10ml |
| 87035998 | SULFAMETHOXAZOLE + TRIMETHOPRIM conc soln inf 800mg + 160mg/10ml |
| 87036998 | TRIMETHOPRIM + SULFAMETHOXAZOLE conc soln inf 80mg + 400mg/5ml |
| 87037998 | SULFAMETHOXAZOLE + TRIMETHOPRIM conc soln inf 400mg + 80mg/5ml |
| 87038998 | CO-TRIMOXAZOLE conc soln inf 160mg + 800mg/10ml |
| 87039998 | CO-TRIMOXAZOLE conc soln inf 80mg + 400mg/5ml |
| 87688998 | SULFAMETHOXAZOLE + TRIMETHOPRIM conc soln inf 80mg + 16mg/ml |
| 87689998 | SULFAMETHOXAZOLE + TRIMETHOPRIM oral susp 400mg + 80mg/5ml |
| 87690998 | SULFAMETHOXAZOLE + TRIMETHOPRIM oral susp 200mg + 40mg/5ml |
| 87691998 | SULFAMETHOXAZOLE + TRIMETHOPRIM tabs 800mg + 160mg |
| 87692998 | TRIMETHOPRIM + SULFAMETHOXAZOLE conc soln inf 16mg + 80mg/ml |
| 87693998 | TRIMETHOPRIM + SULFAMETHOXAZOLE oral susp 80mg + 400mg/5ml |
| 87694998 | TRIMETHOPRIM + SULFAMETHOXAZOLE oral susp 40mg + 200mg/5ml |
| 87695998 | TRIMETHOPRIM + SULFAMETHOXAZOLE tabs 160mg+800mg |
| 87696998 | TRIMETHOPRIM + SULFAMETHOXAZOLE tabs 80mg+400mg |
| 91281998 | PHTHALYLSULFATHIAZOLE tabs 500mg |
| 92368990 | TRIMETHOPRIM susp 50mg/5ml |
| 92552990 | CO-TRIMOXAZOLE tabs 80mg+400mg |
| 92759990 | TRIMETHOPRIM susp 50mg/5ml |
| 92940998 | TRIMETHOPRIM tabs 200mg |
| 93520990 | SULFADIAZINE tabs 500mg |
| 93865990 | TRIMETHOPRIM tabs 200mg |
| 93866990 | TRIMETHOPRIM tabs 100mg |
| 93957996 | CO-TRIMOXAZOLE paed oral susp 40mg + 200mg/5ml |
| 93957997 | CO-TRIMOXAZOLE tabs 160mg+800mg |
| 93957998 | CO-TRIMOXAZOLE tabs 80mg+400mg |
| 93982992 | SULPHAMETHAZINE MIX |
| 94079992 | BACTRIM PAED 120 MG TAB |
| 94119992 | CO-TRIMOXAZOLE 800 MG TAB |
| 94225992 | MADRIBON .5 GM TAB |
| 94378992 | TRIMETHOPRIM 100 MG CAP |
| 94383992 | UROLUCOSIL 100 MG SYR |
| 94404990 | TRIMETHOPRIM tabs 200mg |
| 94405990 | TRIMETHOPRIM tabs 100mg |
| 94483992 | KELFIZINE W PAED 1 GM SYR |
| 94506990 | CO-TRIMOXAZOLE conc soln inf 160mg + 800mg/10ml |
| 94507990 | CO-TRIMOXAZOLE conc soln inf 80mg + 400mg/5ml |
| 94553992 | SYRAPRIM ML INJ |
| 94817998 | SULFADIMIDINE paed mix 500mg/5ml |
| 94842992 | CO-TRIMOXAZOLE 80 MG SYR |
| 94844992 | CO-TRIMOXAZOLE 200 MG SUS |
| 94849992 | CO-TRIMOXAZOLE 100 MG TAB |
| 94850992 | CO-TRIMOXAZOLE PAED 200 MG SYR |
| 94850996 | TRIMETHOPRIM inj 20mg/ml |
| 94850997 | TRIMETHOPRIM susp 50mg/5ml |
| 94850998 | TRIMETHOPRIM tabs 300mg |
| 94851992 | C0-TRIMOXAZOLE TAB |
| 94851997 | TRIMETHOPRIM tabs 200mg |
| 94851998 | TRIMETHOPRIM tabs 100mg |
| 94852998 | SULFADIAZINE tabs 500mg |
| 94863998 | SULFAMETHOXAZOLE + TRIMETHOPRIM tabs 400mg + 80mg |
| 95043992 | GANTRISIN .5 GM TAB |
| 95044992 | GANTRISIN .5 GM SYR |
| 95067990 | TRIMETHOPRIM tabs 200mg |
| 95068990 | TRIMETHOPRIM tabs 100mg |
| 95108997 | TRIMETHOPRIM tabs 200mg |
| 95108998 | TRIMETHOPRIM tabs 100mg |
| 95109997 | TRIMETHOPRIM tabs 200mg |
| 95109998 | TRIMETHOPRIM tabs 100mg |
| 95185992 | KELFIZINE W 2 GM SYR |
| 95211992 | LEDERKYN 500 MG TAB |
| 95233997 | SULFAMETOPYRAZINE susp 500mg/5ml |
| 95233998 | SULFAMETOPYRAZINE tabs 2g |
| 95234998 | SULFAGUANIDINE tabs 500mg |
| 95235997 | SULPHAFURAZOLE syrp 500mg/5ml |
| 95235998 | SULPHAFURAZOLE tabs 500mg |
| 95236998 | SULFADIMIDINE inj 333mg/ml |
| 95237998 | SULPHADIMETHOXINE tabs 500mg |
| 95239998 | SULFADIAZINE inj 250mg/ml |
| 95348998 | CO-TRIMOXAZOLE conc soln inf 16mg + 80mg/ml |
| 95349998 | CO-TRIMOXAZOLE conc soln inf 80mg + 400mg/5ml |
| 95350998 | CO-TRIMOXAZOLE IM inj 320mg/ml |
| 95351998 | CO-TRIMOXAZOLE conc soln inf 16mg + 80mg/ml |
| 95352998 | CO-TRIMOXAZOLE IM inj 320mg/ml |
| 95353998 | CO-TRIMOXAZOLE ADULT oral susp 80mg + 400mg/5ml |
| 95354998 | CO-TRIMOXAZOLE paed oral susp 40mg + 200mg/5ml |
| 95563998 | PHENAZOPYRIDINE + SULPHAUREA tabs |
| 95590990 | TRIMETHOPRIM tabs 200mg |
| 95591990 | TRIMETHOPRIM tabs 100mg |
| 95617992 | SUCCINYLSULPHATHIAZOLE PAED SYR |
| 95618992 | STREPTOTRIAD TAB |
| 95626992 | SULFAMTOPYRAZINE 500 MG SYR |
| 95716992 | UROLUCOSIL 100 MG TAB |
| 95729990 | TRIMETHOPRIM susp 50mg/5ml |
| 95999992 | SUCCINYLSULPHATHIAZOLE POW |
| 96003992 | SULPHATRIAD SYR |
| 96083992 | SULPHADIMIDINE 250 MG TAB |
| 96169992 | COMIXCO 160 800 MG TAB |
| 96470992 | SULPHAPYRIDINE 250 MG TAB |
| 96471992 | SULPHAMEZATHINE .5 GM TAB |
| 96472992 | SULPHAMETHIZOLE 100 MG SYR |
| 96473992 | SULPHAUREA/PHENAZOPYRIDINE HCl 500 MG TAB |
| 96474992 | SULPHAPYRIDINE 500 MG TAB |
| 96502992 | UNITRIM 100 MG TAB |
| 96618992 | CO-TRIFAMOLE 480 MG TAB |
| 96749990 | CO-TRIMOXAZOLE conc soln inf 16mg + 80mg/ml |
| 96776992 | SULPHAPHENAZOLE 500 MG SYR |
| 96777992 | SULPHAMETHIZOLE 100 MG TAB |
| 96894998 | CALCIUM SULPHALOXATE tabs 500mg |
| 96895998 | CALCIUM SULPHALOXATE tabs 500mg |
| 97006998 | SULFADIMIDINE tabs 500mg |
| 97012990 | SULFADIAZINE inj 250mg/ml |
| 97123989 | CO-TRIMOXAZOLE ADULT oral susp 80mg + 400mg/5ml |
| 97123990 | CO-TRIMOXAZOLE paed oral susp 40mg + 200mg/5ml |
| 97168992 | CO-TRIMOXAZOLE F/C 480 MG TAB |
| 97169992 | CO-TRIMOXAZOLE 96 MG INJ |
| 97170992 | CO-TRIMAZINE 500 MG TAB |
| 97171992 | CO-TRIMAZINE 250 MG SYR |
| 97176992 | CO-TRIMOXAZOLE PAED 120 MG TAB |
| 97181998 | CO-TRIMOXAZOLE paed tabs 20mg+100mg |
| 97182998 | CO-TRIMOXAZOLE tabs 160mg+800mg |
| 97183998 | CO-TRIMOXAZOLE disp tab 80mg+400mg |
| 97184998 | CO-TRIMOXAZOLE paed oral susp 40mg + 200mg/5ml |
| 97185998 | CO-TRIMOXAZOLE tabs 160mg+800mg |
| 97186997 | CO-TRIMOXAZOLE disp tab 80mg+400mg |
| 97186998 | CO-TRIMOXAZOLE tabs 80mg+400mg |
| 97187998 | CO-TRIMOXAZOLE paed oral susp 40mg + 200mg/5ml |
| 97191992 | COMIXCO PAED SUSP 40 200 MG/5ML SUS |
| 97198997 | CO-TRIMOXAZOLE paed tabs 40mg+200mg |
| 97198998 | CO-TRIMOXAZOLE paed tabs 20mg+100mg |
| 97199998 | CO-TRIMOXAZOLE disp tab 160mg+800mg |
| 97200996 | CO-TRIMOXAZOLE tabs 160mg+800mg |
| 97200997 | CO-TRIMOXAZOLE disp tab 80mg+400mg |
| 97200998 | CO-TRIMOXAZOLE tabs 80mg+400mg |
| 97351998 | CO-TRIMOXAZOLE paed oral susp 40mg + 200mg/5ml |
| 97352998 | CO-TRIMOXAZOLE ADULT oral susp 80mg + 400mg/5ml |
| 97353998 | CO-TRIMOXAZOLE tabs 160mg+800mg |
| 97354998 | CO-TRIMOXAZOLE tabs 80mg+400mg |
| 97561992 | GX CO-TRIMOXAZOLE 480 MG TAB |
| 97712989 | TRIMETHOPRIM tabs 200mg |
| 97712990 | TRIMETHOPRIM tabs 100mg |
| 98008990 | CO-TRIMOXAZOLE tabs 80mg+400mg |
| 98027989 | CO-TRIMOXAZOLE disp tab 80mg+400mg |
| 98027990 | CO-TRIMOXAZOLE tabs 160mg+800mg |
| 98033992 | SULPHADIAZINE /SULPHAMERAZINE /SULPHATHI 185 MG TAB |
| 98132992 | SUCCINYLSULPHATHIAZOLE SUP |
| 98133992 | SUCCINYLSULPHATHIAZOLE SYR |
| 98134992 | SUCCINYLSULPHATHIAZOLE 500 MG TAB |
| 98136990 | CO-TRIMOXAZOLE tabs 80mg+400mg |
| 98140992 | SULFASUXIDINE 3.5 MG SUP |
| 98141992 | SULPHAPYRIDINE 2 MG TAB |
| 98142992 | SULPHAPHENAZOLE 500 MG TAB |
| 98143992 | SULPHAMETHOXYPYRIDAZINE 500 MG TAB |
| 98144992 | SULPHADIMIDINE 1 GM TAB |
| 98145992 | SULPHADIAZINE M & B TAB |
| 98146992 | SULPHACETAMIDE SYR |
| 98150992 | SULPHATRIAD .5 GM TAB |
| 98151992 | SULPHATHIAZOLE 500 MG TAB |
| 98178990 | CO-TRIMOXAZOLE tabs 80mg+400mg |
| 98182988 | CO-TRIMOXAZOLE tabs 160mg+800mg |
| 98182989 | CO-TRIMOXAZOLE disp tab 80mg+400mg |
| 98182990 | CO-TRIMOXAZOLE paed oral susp 40mg + 200mg/5ml |
| 98236998 | TRIMETHOPRIM inj 20mg/ml |
| 98365998 | TRIMETHOPRIM susp 50mg/5ml |
| 98378992 | UNITRIM 200 MG TAB |
| 98379992 | UROLUCOSIL 500 MG TAB |
| 98387989 | TRIMETHOPRIM tabs 100mg |
| 98387990 | TRIMETHOPRIM tabs 200mg |
| 98398989 | TRIMETHOPRIM tabs 200mg |
| 98398990 | TRIMETHOPRIM tabs 100mg |
| 98399989 | TRIMETHOPRIM tabs 200mg |
| 98399990 | TRIMETHOPRIM tabs 100mg |
| 98519997 | CO-TRIMOXAZOLE paed oral susp 40mg + 200mg/5ml |
| 98519998 | CO-TRIMOXAZOLE paed tabs 20mg+100mg |
| 98520998 | CO-TRIMOXAZOLE IM inj 320mg/ml |
| 98521998 | CO-TRIMOXAZOLE tabs 80mg+400mg |
| 98522998 | CO-TRIMOXAZOLE tabs 160mg+800mg |
| 98523998 | CO-TRIMOXAZOLE ADULT oral susp 80mg + 400mg/5ml |
| 98611997 | CO-TRIMOXAZOLE paed oral susp 40mg + 200mg/5ml |
| 98611998 | CO-TRIMOXAZOLE paed tabs 20mg+100mg |
| 98612998 | CO-TRIMOXAZOLE ADULT oral susp 80mg + 400mg/5ml |
| 98613998 | CO-TRIMOXAZOLE tabs 160mg+800mg |
| 98669988 | TRIMETHOPRIM susp 50mg/5ml |
| 98669989 | TRIMETHOPRIM tabs 100mg |
| 98669990 | TRIMETHOPRIM tabs 200mg |
| 98922990 | SULFADIMIDINE paed mix 500mg/5ml |
| 98923990 | SULFADIMIDINE tabs 500mg |
| 98924990 | SULFADIAZINE inj 250mg/ml |
| 98925990 | SULFADIAZINE tabs 500mg |
| 99025996 | TRIMETHOPRIM susp 50mg/5ml |
| 99025997 | TRIMETHOPRIM tabs 200mg |
| 99025998 | TRIMETHOPRIM tabs 100mg |
| 99052998 | PHTHALYLSULFATHIAZOLE tabs 500mg |
| 99080997 | TRIMETHOPRIM tabs 300mg |
| 99080998 | TRIMETHOPRIM tabs 100mg |
| 99096998 | SULFADIMIDINE inj 333mg/ml |
| 99143997 | CO-TRIMOXAZOLE disp tab 80mg+400mg |
| 99143998 | CO-TRIMOXAZOLE tabs 80mg+400mg |
| 99243989 | CO-TRIMOXAZOLE tabs 160mg+800mg |
| 99243990 | CO-TRIMOXAZOLE tabs 80mg+400mg |
| 99340989 | TRIMETHOPRIM tabs 200mg |
| 99340990 | TRIMETHOPRIM tabs 100mg |
| 99341989 | TRIMETHOPRIM tabs 200mg |
| 99341990 | TRIMETHOPRIM tabs 100mg |
| 99342989 | TRIMETHOPRIM tabs 200mg |
| 99342990 | TRIMETHOPRIM tabs 100mg |
| 99399998 | TRIMETHOPRIM inj 20mg/ml |
| 99400996 | TRIMETHOPRIM susp 50mg/5ml |
| 99400997 | TRIMETHOPRIM tabs 200mg |
| 99400998 | TRIMETHOPRIM tabs 100mg |
| 99453998 | SULPHADIMETHOXINE tabs 500mg |
| 99515997 | SULFAMETOPYRAZINE susp 500mg/5ml |
| 99515998 | SULFAMETOPYRAZINE tabs 2g |
| 99527997 | TRIMETHOPRIM tabs 200mg |
| 99527998 | TRIMETHOPRIM tabs 100mg |
| 99605997 | SULPHAFURAZOLE syrp 500mg/5ml |
| 99605998 | SULPHAFURAZOLE tabs 500mg |
| 99659988 | CO-TRIMOXAZOLE tabs 160mg+800mg |
| 99659989 | CO-TRIMOXAZOLE paed oral susp 40mg + 200mg/5ml |
| 99659990 | CO-TRIMOXAZOLE tabs 80mg+400mg |
| 99660989 | CO-TRIMOXAZOLE paed oral susp 40mg + 200mg/5ml |
| 99660990 | CO-TRIMOXAZOLE tabs 80mg+400mg |
| 99661988 | CO-TRIMOXAZOLE tabs 160mg+800mg |
| 99661989 | CO-TRIMOXAZOLE paed oral susp 40mg + 200mg/5ml |
| 99661990 | CO-TRIMOXAZOLE tabs 80mg+400mg |
| 99749992 | BACTRIM DRAPSULES CAP |
| 99919998 | CO-TRIMOXAZOLE disp tab 80mg+400mg |
| 82730998 | DOXYCYCLINE MONOHYDRATE mr cap 40mg |
| 82732998 | DOXYCYCLINE MONOHYDRATE mr cap 40mg |
| 83932998 | MINOCYCLINE oral liq |
| 83942998 | OXYTETRACYCLINE oral liq |
| 84347998 | DEMECLOCYCLINE oral liq |
| 84948998 | DOXYCYCLINE (AS HYCLATE) oral liq |
| 86032998 | TIGECYCLINE pdr/inf.soln. 50mg/vial |
| 86033998 | TIGECYCLINE pdr/inf.soln. 50mg/vial |
| 86390998 | MINOCYCLINE mr cap 100mg |
| 86753998 | MINOCYCLINE mr cap 100mg |
| 87156998 | BECLOMETASONE oint + CHLORTETRACYCLINE IN WHITE SOFT PARAFFIN (BCM) oint 25% + 3% |
| 87959998 | MINOCYCLINE mr cap 100mg |
| 88030998 | DOXYCYCLINE (AS HYCLATE) cap.with micrograns. 50mg |
| 88271998 | DOXYCYCLINE (AS HYCLATE) cap.with micrograns. 50mg |
| 88431998 | DOXYCYCLINE (AS HYCLATE) caps 100mg |
| 89009998 | DEMECLOCYCLINE caps 150mg |
| 89467998 | DOXYCYCLINE (AS HYCLATE) tabs 20mg |
| 90801998 | DOXYCYCLINE (AS HYCLATE) tabs 20mg |
| 91262998 | OXYTETRACYCLINE + BROMHEXINE HCl caps 250mg + 8mg |
| 91308998 | MINOCYCLINE tabs 100mg |
| 91630998 | DOXYCYCLINE MONOHYDRATE disp tab 100mg |
| 92362998 | DOXYCYCLINE (AS HYCLATE) caps 100mg |
| 92481998 | TETRACYCLINE + CHLORTETRACYCLINE & DEMECLOCYCLINE tabs |
| 92556998 | DOXYCYCLINE (AS HYCLATE) caps 20mg |
| 92580997 | MINOCYCLINE tabs 100mg |
| 92580998 | MINOCYCLINE tabs 50mg |
| 92601997 | MINOCYCLINE tabs 100mg |
| 92601998 | MINOCYCLINE tabs 50mg |
| 92613997 | DOXYCYCLINE (AS HYCLATE) cap.with micrograns. 50mg |
| 92613998 | DOXYCYCLINE (AS HYCLATE) caps 50mg |
| 92774990 | DOXYCYCLINE (AS HYCLATE) caps 50mg |
| 92775990 | DOXYCYCLINE (AS HYCLATE) caps 100mg |
| 92854997 | MINOCYCLINE caps 100mg |
| 92854998 | MINOCYCLINE caps 50mg |
| 92856997 | DOXYCYCLINE (AS HYCLATE) caps 50mg |
| 92856998 | DOXYCYCLINE (AS HYCLATE) caps 100mg |
| 92880990 | DEMECLOCYCLINE caps 150mg |
| 92931998 | MINOCYCLINE tabs 50mg |
| 93024998 | MINOCYCLINE dental gel 2% |
| 93276992 | CHLORTETRACYCLINE HYD./DEMECLOCYCLINE HY 115.4 MG TAB |
| 93484992 | CYCLODOX CAP 100 mg |
| 93707992 | DETECLO 75 MG SYR |
| 93711992 | GALENOMYCIN TAB |
| 93725992 | DOXATET INJ |
| 93837997 | TETRACYCLINE syrp 125mg/5ml |
| 93837998 | TETRACYCLINE caps 250mg |
| 93855990 | MINOCYCLINE caps 100mg |
| 93923998 | DOXYCYCLINE (AS HYCLATE) caps 100mg |
| 93938998 | OXYTETRACYCLINE tabs 250mg |
| 93986992 | TETRACYCLINE HCL/PANCREATIC CONCENTRATE CAP |
| 93987992 | TETRACYCLINE HYDROCHLORIDE/AMPHOTERICIN SYR |
| 94007992 | LEDERMYCIN DROPS 60 MG |
| 94158990 | MINOCYCLINE tabs 100mg |
| 94398990 | OXYTETRACYCLINE tabs 250mg |
| 94446990 | OXYTETRACYCLINE tabs 250mg |
| 94561996 | TETRACYCLINE IV inj 500mg/vial |
| 94561997 | TETRACYCLINE IV inj 250mg/vial |
| 94561998 | TETRACYCLINE IM inj 100mg/vial |
| 94666998 | CHLORTETRACYCLINE + DEMECLOCYCLINE & TETRACYCLINE tabs |
| 94750990 | OXYTETRACYCLINE tabs 250mg |
| 94848990 | MINOCYCLINE mr cap 100mg |
| 94933998 | LYMECYCLINE caps 408mg |
| 95182990 | MINOCYCLINE tabs 100mg |
| 95183990 | MINOCYCLINE tabs 50mg |
| 95189998 | TETRACYCLINE + PANCREATIC ENZYMES caps |
| 95190998 | TETRACYCLINE + AMPHOTERACIN syrp |
| 95191998 | TETRACYCLINE + NYSTATIN tabs 250mg + 250,000 units |
| 95192998 | TETRACYCLINE + CHLORTETRACYCLINE & DEMECLOCYCLINE tabs |
| 95193998 | TETRACYCLINE caps 250mg |
| 95194998 | TETRACYCLINE mr cap 250mg |
| 95195996 | TETRACYCLINE IV inj 500mg/vial |
| 95195997 | TETRACYCLINE IV inj 250mg/vial |
| 95195998 | TETRACYCLINE IM inj 100mg/vial |
| 95196996 | TETRACYCLINE pwdr |
| 95196997 | TETRACYCLINE caps 250mg |
| 95196998 | TETRACYCLINE syrp 125mg/5ml |
| 95210992 | LEDERMYCIN 75 MG SYR |
| 95369992 | OXYTETRACYCLINE 500 MG TAB |
| 95379990 | MINOCYCLINE tabs 50mg |
| 95640998 | OXYTETRACYCLINE syrp 125mg/5ml |
| 95644997 | OXYTETRACYCLINE syrp 125mg/5ml |
| 95644998 | OXYTETRACYCLINE caps 250mg |
| 95654992 | TERRA-BRON TAB |
| 95659992 | TETREX CAP |
| 95660992 | TETRALYSAL 150 MG CAP |
| 95680996 | NYSTATIN + TETRACYCLINE HCl syrp |
| 95680997 | NYSTATIN + TETRACYCLINE HCl tabs |
| 95680998 | NYSTATIN + TETRACYCLINE HCl caps |
| 95801997 | MINOCYCLINE tabs 100mg |
| 95801998 | MINOCYCLINE tabs 50mg |
| 95828992 | CHLORTETRACYCLINE HCl SYR |
| 95883998 | METACYCLINE caps 150mg |
| 95884998 | METACYCLINE caps 150mg |
| 95991998 | LYMECYCLINE caps 408mg |
| 96075990 | MINOCYCLINE tabs 100mg |
| 96076990 | MINOCYCLINE tabs 50mg |
| 96089990 | DOXYCYCLINE (AS HYCLATE) caps 100mg |
| 96094992 | BLENMIX 100 MG TAB |
| 96178992 | DEMECLOCYCLINE HCl 75 MG SYR |
| 96202990 | DOXYCYCLINE (AS HYCLATE) caps 50mg |
| 96282990 | DOXYCYCLINE (AS HYCLATE) tabs 100mg |
| 96304997 | DOXYCYCLINE (AS HYCLATE) caps 20mg |
| 96304998 | DOXYCYCLINE (AS HYCLATE) syrp 50mg/5ml |
| 96305996 | DOXYCYCLINE (AS HYCLATE) tabs 100mg |
| 96305997 | DOXYCYCLINE (AS HYCLATE) caps 100mg |
| 96305998 | DOXYCYCLINE (AS HYCLATE) disp tab 100mg |
| 96329992 | METHACYCLINE HCl 150 MG CAP |
| 96354990 | DOXYCYCLINE (AS HYCLATE) caps 100mg |
| 96367992 | NOVOBIOCIN/TETRACYCLINE 125 MG CAP |
| 96447998 | DEMECLOCYCLINE + CHLORTETRACYCLINE & TETRACYCLINE tabs |
| 96448997 | DEMECLOCYCLINE tabs 300mg |
| 96448998 | DEMECLOCYCLINE caps 150mg |
| 96484992 | TETRACYCLINE/PROCAINE HCl 100 MG INJ |
| 96485992 | TETRACYCLINE 500 MG TAB |
| 96635998 | CLOMOCYCLINE caps 170mg |
| 96636998 | CLOMOCYCLINE caps 170mg |
| 96681997 | CHLORTETRACYCLINE pwdr |
| 96681998 | CHLORTETRACYCLINE caps 250mg |
| 96779992 | TERRAMYCIN SYR |
| 96781992 | TETRACYCLINE 500 MG CAP |
| 96826992 | TETRACHEL 200 MG TAB |
| 96835992 | BLENMIX 50 MG TAB |
| 96979990 | MINOCYCLINE tabs 100mg |
| 96980989 | MINOCYCLINE tabs 100mg |
| 96980990 | MINOCYCLINE tabs 50mg |
| 96989990 | TETRACYCLINE tabs 250mg |
| 96994992 | BROMHEXINE HCL/OXYTETRACYCLINE HCL CAP |
| 97051990 | DOXYCYCLINE (AS HYCLATE) caps 100mg |
| 97121989 | DOXYCYCLINE (AS HYCLATE) caps 50mg |
| 97121990 | DOXYCYCLINE (AS HYCLATE) caps 100mg |
| 97142997 | TETRACYCLINE tabs 250mg |
| 97142998 | TETRACYCLINE caps 250mg |
| 97143998 | OXYTETRACYCLINE caps 250mg |
| 97144998 | OXYTETRACYCLINE tabs 250mg |
| 97145998 | OXYTETRACYCLINE tabs 250mg |
| 97146998 | OXYTETRACYCLINE tabs 250mg |
| 97153997 | TETRACYCLINE syrp 125mg/5ml |
| 97153998 | TETRACYCLINE tabs 250mg |
| 97154998 | OXYTETRACYCLINE tabs 250mg |
| 97209989 | DOXYCYCLINE (AS HYCLATE) caps 100mg |
| 97209990 | DOXYCYCLINE (AS HYCLATE) caps 50mg |
| 97246992 | DEMIX 100 MG CAP |
| 97247990 | DOXYCYCLINE (AS HYCLATE) caps 50mg |
| 97247992 | DEMECLOCYCLINE HCl DROPS 60 MG DRO |
| 97559997 | MINOCYCLINE tabs 100mg |
| 97559998 | MINOCYCLINE tabs 50mg |
| 97683992 | LYMECYCLINE 204 MG CAP |
| 97711998 | DOXYCYCLINE (AS HYCLATE) caps 100mg |
| 97732990 | OXYTETRACYCLINE tabs 250mg |
| 97735992 | METHACYCLINE HCl 75 MG SYR |
| 97738989 | MINOCYCLINE tabs 100mg |
| 97738990 | MINOCYCLINE tabs 50mg |
| 97753998 | DOXYCYCLINE (AS HYCLATE) caps 50mg |
| 97761989 | DOXYCYCLINE (AS HYCLATE) caps 100mg |
| 97761990 | DOXYCYCLINE (AS HYCLATE) caps 50mg |
| 97800989 | MINOCYCLINE tabs 100mg |
| 97800990 | MINOCYCLINE tabs 50mg |
| 97815992 | NOVOBIOCIN/TETRACYCLINE 62.5 MG MIX |
| 97841992 | OXYTETRACYCLINE/PROCAINE HCl 100 MG INJ |
| 97842992 | OXYTETRACYCLINE 250 MG SYR |
| 97843992 | OXYTETRACYCLINE 100 MG TAB |
| 97844992 | OXYTETRACYCLINE 250 MG INJ |
| 97873989 | MINOCYCLINE tabs 100mg |
| 97873990 | MINOCYCLINE tabs 50mg |
| 97892998 | TETRACYCLINE mr cap 250mg |
| 97913998 | DOXYCYCLINE (AS HYCLATE) tabs 100mg |
| 98029989 | MINOCYCLINE tabs 100mg |
| 98029990 | MINOCYCLINE tabs 50mg |
| 98044990 | DOXYCYCLINE (AS HYCLATE) caps 100mg |
| 98231998 | DOXYCYCLINE MONOHYDRATE disp tab 100mg |
| 98323997 | TETRACYCLINE syrp 125mg/5ml |
| 98323998 | TETRACYCLINE caps 250mg |
| 98341996 | TETRACYCLINE pwdr |
| 98341997 | TETRACYCLINE caps 250mg |
| 98341998 | TETRACYCLINE tabs 250mg |
| 98352989 | DOXYCYCLINE (AS HYCLATE) caps 50mg |
| 98352990 | DOXYCYCLINE (AS HYCLATE) caps 100mg |
| 98405990 | TETRACYCLINE tabs 250mg |
| 98456998 | OXYTETRACYCLINE tabs 250mg |
| 98462990 | OXYTETRACYCLINE tabs 250mg |
| 98463990 | OXYTETRACYCLINE tabs 250mg |
| 98464990 | OXYTETRACYCLINE tabs 250mg |
| 98480989 | MINOCYCLINE tabs 100mg |
| 98480990 | MINOCYCLINE tabs 50mg |
| 98530998 | MINOCYCLINE mr cap 100mg |
| 98531996 | MINOCYCLINE caps 100mg |
| 98531997 | MINOCYCLINE caps 50mg |
| 98531998 | MINOCYCLINE mr cap 100mg |
| 98601989 | DOXYCYCLINE (AS HYCLATE) caps 100mg |
| 98601990 | DOXYCYCLINE (AS HYCLATE) caps 50mg |
| 98612990 | TETRACYCLINE caps 250mg |
| 98969996 | DOXYCYCLINE (AS HYCLATE) syrp 50mg/5ml |
| 98969997 | DOXYCYCLINE MONOHYDRATE disp tab 100mg |
| 98969998 | DOXYCYCLINE (AS HYCLATE) caps 100mg |
| 99043990 | TETRACYCLINE tabs 250mg |
| 99053998 | TETRACYCLINE caps 250mg |
| 99054998 | LYMECYCLINE caps 408mg |
| 99055997 | TETRACYCLINE caps 250mg |
| 99055998 | TETRACYCLINE tabs 250mg |
| 99056998 | TETRACYCLINE caps 250mg |
| 99060997 | OXYTETRACYCLINE caps 250mg |
| 99060998 | OXYTETRACYCLINE tabs 250mg |
| 99101997 | DOXYCYCLINE (AS HYCLATE) caps 50mg |
| 99101998 | DOXYCYCLINE (AS HYCLATE) caps 100mg |
| 99354990 | TETRACYCLINE tabs 250mg |
| 99355990 | TETRACYCLINE tabs 250mg |
| 99356990 | TETRACYCLINE tabs 250mg |
| 99382997 | TETRACYCLINE + NYSTATIN tabs 250mg + 250,000 units |
| 99478990 | OXYTETRACYCLINE tabs 250mg |
| 99479990 | OXYTETRACYCLINE tabs 250mg |
| 99480990 | OXYTETRACYCLINE tabs 250mg |
| 99485997 | DEMECLOCYCLINE tabs 300mg |
| 99485998 | DEMECLOCYCLINE caps 150mg |
| 99613989 | DOXYCYCLINE (AS HYCLATE) caps 50mg |
| 99613990 | DOXYCYCLINE (AS HYCLATE) caps 100mg |
| 99767998 | TETRACYCLINE + CHLORTETRACYCLINE & DEMECLOCYCLINE tabs |
| 99884998 | OXYTETRACYCLINE + BROMHEXINE HCl caps 250mg + 8mg |
| 99895998 | OXYTETRACYCLINE tabs 250mg |
| 99923997 | CHLORTETRACYCLINE pwdr |
| 99923998 | CHLORTETRACYCLINE caps 250mg |
| 83936998 | NITROFURANTOIN oral liq |
| 92816997 | FOSFOMYCIN paed sach 2g |
| 92816998 | FOSFOMYCIN sach 3g |
| 92817997 | FOSFOMYCIN paed sach 2g |
| 92817998 | FOSFOMYCIN sach 3g |
| 93792990 | NITROFURANTOIN tabs 100mg |
| 93793990 | NITROFURANTOIN tabs 50mg |
| 93815990 | NITROFURANTOIN tabs 100mg |
| 93816990 | NITROFURANTOIN tabs 50mg |
| 93840992 | CEDURAN TAB |
| 94229992 | MANDELAMINE .25 GM TAB |
| 94684990 | NITROFURANTOIN tabs 100mg |
| 94685990 | NITROFURANTOIN tabs 50mg |
| 94715992 | BERKFURIN 50 MG TAB |
| 95008990 | NITROFURANTOIN tabs 100mg |
| 95009990 | NITROFURANTOIN tabs 50mg |
| 95200990 | NITROFURANTOIN susp 25mg/5ml |
| 95245992 | MANDELAMINE .5 GM TAB |
| 95714997 | NITROFURANTOIN tabs 100mg |
| 95714998 | NITROFURANTOIN tabs 50mg |
| 95715996 | NITROFURANTOIN mr cap 100mg |
| 95715997 | NITROFURANTOIN susp 25mg/5ml |
| 95715998 | NITROFURANTOIN caps 100mg |
| 95716996 | NITROFURANTOIN caps 50mg |
| 95716997 | NITROFURANTOIN tabs 100mg |
| 95716998 | NITROFURANTOIN tabs 50mg |
| 95876998 | METHENAMINE HIPPURATE tabs 1g |
| 95877998 | METHENAMINE HIPPURATE tabs 300mg |
| 96266992 | HEXAMINE MANDELATE 500 MG TAB |
| 96722992 | NITROFURANTOIN 25 MG TAB |
| 97216989 | NITROFURANTOIN tabs 100mg |
| 97216990 | NITROFURANTOIN tabs 50mg |
| 97494992 | FURADANTIN 50 MG SUS |
| 97574992 | HEXAMINE MANDELATE 250 MG TAB |
| 97677998 | NITROFURANTOIN mr cap 100mg |
| 97804992 | NITROFURANTOIN/DEGLYCYRRIZINISED EXTRACT TAB |
| 97980989 | NITROFURANTOIN tabs 100mg |
| 97980990 | NITROFURANTOIN tabs 50mg |
| 99454997 | NITROFURANTOIN caps 50mg |
| 99454998 | NITROFURANTOIN caps 100mg |
| 99560998 | METHENAMINE HIPPURATE tabs 1g |
| 99609996 | NITROFURANTOIN susp 25mg/5ml |
| 99609997 | NITROFURANTOIN tabs 100mg |
| 99609998 | NITROFURANTOIN tabs 50mg |
| 99952992 | HEXAMINE MANDELATE/METHIONINE 250 MG TAB |
