# Supplementary material for: Association between Antibiotic Prescribing in Pregnancy and Cerebral Palsy or Epilepsy in Children Born at Term: A Cohort Study Using The Health Improvement Network
Source: PLoS One. 2015 Mar 25;10(3):e0122034. doi: 10.1371/journal.pone.0122034 (PMC4373729; doi:10.1371/journal.pone.0122034)
Supplement: S2 Appendix — (DOCX) [file pone.0122034.s002.docx]

**S2 Appendix – Description of algorithm and codes used to identify children with epilepsy.**

Code lists below were created by Wilhelmine Meeraus with expert advice and input from: Prof Ruth Gilbert (paediatrician, epidemiologist); Dr Irene Petersen (statistician, primary care data epidemiologist); Dr Felixity Knott (GP, epidemiologist); and Dr Richard chin (epileptologist).

A child was considered to have epilepsy if they had two or more codes for epilepsy treatment in their medical record and these codes had been recorded no more four months apart, or the child had at least one code for a diagnosis, or the child had two or more epilepsy symptoms recorded on separate days.

The full medical records of a 10% sample of children with epilepsy were reviewed for validation purposes. All reviewers were blinded as to whether the children’s mothers were prescribed antibiotics in pregnancy. Out of 116 children reviewed, seven (6%) had no evidence at all (other than repeat prescriptions for Anti-Epileptic Drug, AED) suggestive of epilepsy. None of the seven children had evidence of AED use for other indications and therefore all children identified using the coding algorithm were considered to have epilepsy in this study.

Read Codes for Epilepsy Diagnosis

| Code | Description |
| --- | --- |
| 6110.00 | Contraceptive advice for patients with epilepsy |
| 67AF.00 | Pregnancy advice for patients with epilepsy |
| 67IJ000 | Pre-conception advice for patients with epilepsy |
| 8IAg.00 | Contraceptive advice for patients with epilepsy declined |
| 8IAh.00 | Pre-conception advice for patients with epilepsy declined |
| 8IAi.00 | Pregnancy advice for patients with epilepsy declined |
| 8IB2.00 | Contraceptiv advice for patients with epilepsy not indicated |
| 8IB3.00 | Pre-conception advic fr patients with epilepsy not indicated |
| 8IB4.00 | Pregnancy advice for patients with epilepsy not indicated |
| 9Of5.00 | Epilepsy monitoring call first letter |
| 9Of6.00 | Epilepsy monitoring call second letter |
| 9Of7.00 | Epilepsy monitoring call third letter |
| 13Y9.00 | Epilepsy society member |
| 1B1W.00 | Transient epileptic amnesia |
| 1O30.00 | Epilepsy confirmed |
| 667..00 | Epilepsy monitoring |
| 6674.00 | Epilepsy associated problems |
| 667B.00 | Nocturnal epilepsy |
| 667C.00 | Epilepsy control good |
| 667D.00 | Epilepsy control poor |
| 667E.00 | Epilepsy care arrangement |
| 667G.00 | Epilepsy restricts employment |
| 667H.00 | Epilepsy prevents employment |
| 667J.00 | Epilepsy impairs education |
| 667K.00 | Epilepsy limits activities |
| 667L.00 | Epilepsy does not limit activities |
| 667M.00 | Epilepsy management plan given |
| 667N.00 | Epilepsy severity |
| 9h6..00 | Exception reporting: epilepsy quality indicators |
| 9h61.00 | Excepted from epilepsy quality indicators: Patient unsuitabl |
| 9h62.00 | Excepted from epilepsy quality indicators: Informed dissent |
| Eu05212 | [X]Schizophrenia-like psychosis in epilepsy |
| Eu05y11 | [X]Epileptic psychosis NOS |
| Eu06013 | [X]Limbic epilepsy personality |
| Eu80300 | [X]Acquired aphasia with epilepsy [Landau - Kleffner] |
| F132100 | Progressive myoclonic epilepsy |
| F25..00 | Epilepsy |
| F250.00 | Generalised nonconvulsive epilepsy |
| F250000 | Petit mal (minor) epilepsy |
| F250100 | Pykno-epilepsy |
| F250400 | Juvenile absence epilepsy |
| F250500 | Lennox-Gastaut syndrome |
| F250y00 | Other specified generalised nonconvulsive epilepsy |
| F250z00 | Generalised nonconvulsive epilepsy NOS |
| F251.00 | Generalised convulsive epilepsy |
| F251000 | Grand mal (major) epilepsy |
| F251011 | Tonic-clonic epilepsy |
| F251100 | Neonatal myoclonic epilepsy |
| F251111 | Otohara syndrome |
| F251500 | Tonic-clonic epilepsy |
| F251y00 | Other specified generalised convulsive epilepsy |
| F251z00 | Generalised convulsive epilepsy NOS |
| F254.00 | Partial epilepsy with impairment of consciousness |
| F254000 | Temporal lobe epilepsy |
| F254100 | Psychomotor epilepsy |
| F254200 | Psychosensory epilepsy |
| F254300 | Limbic system epilepsy |
| F254400 | Epileptic automatism |
| F254z00 | Partial epilepsy with impairment of consciousness NOS |
| F255.00 | Partial epilepsy without impairment of consciousness |
| F255000 | Jacksonian focal or motor epilepsy |
| F255011 | Focal epilepsy |
| F255012 | Motor epilepsy |
| F255100 | Sensory induced epilepsy |
| F255200 | Somatosensory epilepsy |
| F255300 | Visceral reflex epilepsy |
| F255311 | Partial epilepsy with autonomic symptoms |
| F255400 | Visual reflex epilepsy |
| F255500 | Unilateral epilepsy |
| F255y00 | Partial epilepsy without impairment of consciousness OS |
| F255z00 | Partial epilepsy without impairment of consciousness NOS |
| F256.00 | Infantile spasms |
| F256.11 | Lightning spasms |
| F256.12 | West syndrome |
| F256000 | Hypsarrhythmia |
| F256100 | Salaam attacks |
| F256z00 | Infantile spasms NOS |
| F257.00 | Kojevnikov's epilepsy |
| F259.00 | Early infant epileptic encephalopathy wth suppression bursts |
| F259.11 | Ohtahara syndrome |
| F25A.00 | Juvenile myoclonic epilepsy |
| F25B.00 | Alcohol-induced epilepsy |
| F25C.00 | Drug-induced epilepsy |
| F25D.00 | Menstrual epilepsy |
| F25E.00 | Stress-induced epilepsy |
| F25F.00 | Photosensitive epilepsy |
| F25y.00 | Other forms of epilepsy |
| F25y000 | Cursive (running) epilepsy |
| F25y100 | Gelastic epilepsy |
| F25y200 | Locl-rlt(foc)(part)idiop epilep&epilptic syn seiz locl onset |
| F25y400 | Benign Rolandic epilepsy |
| F25y500 | Panayiotopoulos syndrome |
| F25yz00 | Other forms of epilepsy NOS |
| F25z.00 | Epilepsy NOS |
| Fyu5000 | [X]Other generalized epilepsy and epileptic syndromes |
| Fyu5100 | [X]Other epilepsy |
| SC20000 | Traumatic epilepsy |
| ZS82.00 | Acquired epileptic aphasia |
| ZS82.11 | Landau-Kleffner syndrome |

Encrypted Multilex Codes for Anti-Epileptic Drug Treatment for Epilepsy

*NB. Encrypted multiplex codes were were used in The Health Improvement Network (THIN) to classify medicinal products and devices prior to their switch to Gemscript codes.*

| Code | Description |
| --- | --- |
| 82574998 | ESLICARBAZEPINE ACETATE tabs 800mg |
| 82576998 | ESLICARBAZEPINE ACETATE tabs 800mg |
| 82713998 | CLOBAZAM oral soln 25mg/5ml |
| 82714998 | CLOBAZAM oral susp 25mg/5ml |
| 82857998 | SODIUM VALPROATE + VALPROIC ACID MR granules 1000mg |
| 83073998 | PHENOBARBITAL oral soln 20mg/5ml |
| 92197990 | TOPIRAMATE tabs 200mg |
| 92198990 | TOPIRAMATE tabs 100mg |
| 92199990 | TOPIRAMATE tabs 50mg |
| 92200990 | TOPIRAMATE tabs 25mg |
| 92279990 | TOPIRAMATE tabs 200mg |
| 92280990 | TOPIRAMATE tabs 100mg |
| 92281990 | TOPIRAMATE tabs 50mg |
| 92282990 | TOPIRAMATE tabs 25mg |
| 92283990 | TOPIRAMATE caps 50mg |
| 92284990 | TOPIRAMATE caps 25mg |
| 92285990 | TOPIRAMATE caps 15mg |
| 92291990 | TOPIRAMATE caps 50mg |
| 92292990 | TOPIRAMATE caps 25mg |
| 92293990 | TOPIRAMATE caps 15mg |
| 92295990 | TOPIRAMATE tabs 200mg |
| 92296990 | TOPIRAMATE tabs 100mg |
| 92297990 | TOPIRAMATE tabs 50mg |
| 92298990 | TOPIRAMATE tabs 25mg |
| 92330990 | TOPIRAMATE tabs 200mg |
| 92331990 | TOPIRAMATE tabs 100mg |
| 92332990 | TOPIRAMATE tabs 50mg |
| 92333990 | TOPIRAMATE tabs 25mg |
| 92356990 | CLONAZEPAM tabs 2mg |
| 92357990 | CLONAZEPAM tabs 500 micrograms |
| 92387990 | OXCARBAZEPINE tabs 600mg |
| 92388990 | OXCARBAZEPINE tabs 300mg |
| 92389990 | OXCARBAZEPINE tabs 150mg |
| 92462990 | GABAPENTIN tabs 800mg |
| 92463990 | GABAPENTIN tabs 600mg |
| 92502990 | GABAPENTIN tabs 800mg |
| 92503990 | GABAPENTIN tabs 600mg |
| 99880998 | TIAGABINE tabs 10mg |
| 99762998 | ACETAZOLAMIDE mr cap 500mg |
| 99752990 | CARBAMAZEPINE tabs 100mg |
| 99752989 | CARBAMAZEPINE tabs 200mg |
| 99752988 | CARBAMAZEPINE tabs 400mg |
| 99751990 | CARBAMAZEPINE tabs 100mg |
| 99751989 | CARBAMAZEPINE tabs 200mg |
| 99751988 | CARBAMAZEPINE tabs 400mg |
| 99720990 | NITRAZEPAM tabs 5mg |
| 99719990 | NITRAZEPAM tabs 5mg |
| 99718990 | NITRAZEPAM tabs 5mg |
| 99717990 | NITRAZEPAM tabs 5mg |
| 99697998 | ETHOSUXIMIDE caps 250mg |
| 99697997 | ETHOSUXIMIDE syrp 250mg/5ml |
| 99694998 | PHENYTOIN + PHENOBARBITAL caps 100mg + 50mg |
| 99692998 | PHENYTOIN SODIUM inj 250mg/5ml |
| 99622998 | CLOBAZAM caps 10mg |
| 99622997 | CLOBAZAM tabs 10mg |
| 99459990 | PHENOBARBITAL tabs 60mg |
| 99459989 | PHENOBARBITAL tabs 30mg |
| 99458990 | PHENOBARBITAL tabs 15mg |
| 99458989 | PHENOBARBITAL tabs 30mg |
| 99458988 | PHENOBARBITAL tabs 60mg |
| 99457990 | PHENOBARBITAL elixir 15mg/5ml |
| 99455990 | PHENYTOIN SODIUM tabs 50mg |
| 99455989 | PHENYTOIN SODIUM tabs 100mg |
| 99454990 | PHENYTOIN SODIUM tabs 50mg |
| 99454989 | PHENYTOIN SODIUM caps 100mg |
| 99453990 | PHENYTOIN SODIUM tabs 100mg |
| 99453989 | PHENYTOIN SODIUM caps 100mg |
| 99432990 | PHENOBARBITAL tabs 100mg |
| 99404998 | NITRAZEPAM tabs 5mg |
| 99383998 | PRIMIDONE tabs 250mg |
| 99383997 | PRIMIDONE oral susp 250mg/5ml |
| 99354998 | NITRAZEPAM tabs 5mg |
| 99332998 | BECLAMIDE tabs 500mg |
| 99176998 | CLONAZEPAM tabs 500 micrograms |
| 99176997 | CLONAZEPAM tabs 2mg |
| 99170998 | FLUNITRAZEPAM tabs 1mg |
| 99142990 | NITRAZEPAM tabs 5mg |
| 99142989 | NITRAZEPAM tabs 10mg |
| 99125998 | NITRAZEPAM susp 2.5mg/5ml |
| 99124990 | PHENOBARBITAL tabs 15mg |
| 99124989 | PHENOBARBITAL tabs 30mg |
| 99124988 | PHENOBARBITAL tabs 60mg |
| 99122990 | PHENYTOIN SODIUM tabs 50mg |
| 99122989 | PHENYTOIN SODIUM tabs 100mg |
| 99121990 | PHENYTOIN SODIUM tabs 50mg |
| 99121989 | PHENYTOIN SODIUM tabs 100mg |
| 99110990 | PHENOBARBITAL tabs 100mg |
| 98991998 | DIAZEPAM caps 10mg |
| 98989998 | Gabapentin tabs 600mg 100 tablet(s) |
| 98989997 | Gabapentin tabs 800mg 100 tablet(s) |
| 98949998 | ETHOSUXIMIDE caps 250mg |
| 98949997 | ETHOSUXIMIDE syrp 250mg/5ml |
| 98929990 | SODIUM VALPROATE ec tab 200mg |
| 98929989 | SODIUM VALPROATE ec tab 500mg |
| 98929988 | SODIUM VALPROATE sf liq 200mg/5ml |
| 98928998 | ACETAZOLAMIDE tabs 250mg |
| 98764990 | PHENOBARBITAL SODIUM pwdr |
| 98739990 | PHENOBARBITAL SODIUM |
| 98730998 | OXCARBAZEPINE tabs 150mg |
| 98688990 | PHENOBARBITAL elixir 15mg/5ml |
| 98675990 | NITRAZEPAM tabs 5mg |
| 98658998 | PHENYTOIN susp 30mg/5ml |
| 98517998 | CLONAZEPAM conc soln inj 1mg/1ml |
| 98476998 | PHENOBARBITAL tabs 30mg |
| 98476997 | PHENOBARBITAL tabs 60mg |
| 98476996 | PHENOBARBITAL tabs 15mg |
| 98470990 | NITRAZEPAM tabs 5mg |
| 98461998 | METHYLPHENOBARBITAL tabs 30mg |
| 98461997 | METHYLPHENOBARBITAL tabs 60mg |
| 98461996 | METHYLPHENOBARBITAL tabs 200mg |
| 98434990 | PHENOBARBITAL inj 60mg/ml |
| 98434989 | PHENOBARBITAL inj 15mg |
| 98434988 | PHENOBARBITAL inj 30mg |
| 98431990 | PHENYTOIN SODIUM inj 250mg/5ml |
| 98430990 | PHENYTOIN SODIUM tabs 100mg |
| 98430989 | PHENYTOIN SODIUM tabs 50mg |
| 98385990 | SODIUM VALPROATE ec tab 200mg |
| 98385989 | SODIUM VALPROATE ec tab 500mg |
| 98361998 | CARBAMAZEPINE tabs 100mg |
| 98361997 | CARBAMAZEPINE tabs 200mg |
| 98361996 | CARBAMAZEPINE tabs 400mg |
| 98360998 | CARBAMAZEPINE liq 100mg/5ml |
| 98338990 | CARBAMAZEPINE tabs 100mg |
| 98338989 | CARBAMAZEPINE tabs 200mg |
| 98338988 | CARBAMAZEPINE tabs 400mg |
| 98328998 | ACETAZOLAMIDE tabs 250mg |
| 98328997 | ACETAZOLAMIDE SODIUM inj 500mg |
| 98315998 | PHENYTOIN SODIUM caps 25mg |
| 98315997 | PHENYTOIN SODIUM caps 50mg |
| 98315996 | PHENYTOIN SODIUM caps 100mg |
| 98200998 | TIAGABINE tabs 5mg |
| 98152992 | SULTHIAME |
| 98148992 | SULTHIAME |
| 98147992 | SULTHIAME |
| 98112990 | PHENOBARBITAL tabs 60mg |
| 98112989 | PHENOBARBITAL tabs 30mg |
| 98112988 | PHENOBARBITAL tabs 15mg |
| 98111990 | PHENOBARBITAL SODIUM inj 200mg/ml |
| 98110990 | PHENYTOIN SODIUM tabs 100mg |
| 98090998 | Phenytoin sodium tabs 50mg 28 tablet(s) |
| 98090997 | Phenytoin sodium tabs 100mg tablet(s) 100 |
| 98088990 | NITRAZEPAM susp 5mg/5ml |
| 98087998 | Phenobarbital elixir 15mg/5ml 2 litre(s) |
| 98087997 | PHENOBARBITAL |
| 98084990 | SODIUM VALPROATE ec tab 500mg |
| 98075990 | PHENYTOIN sf susp 90mg/5ml |
| 98049990 | PHENOBARBITAL tabs 15mg |
| 98049989 | PHENOBARBITAL tabs 30mg |
| 98049988 | PHENOBARBITAL tabs 60mg |
| 97955992 | PRIMIDONE/PHENYTION SODIUM |
| 97949992 | PRIMIDONE |
| 97911990 | SODIUM VALPROATE sf liq 200mg/5ml |
| 97911989 | SODIUM VALPROATE ec tab 200mg |
| 97911988 | SODIUM VALPROATE ec tab 500mg |
| 97910990 | SODIUM VALPROATE crushable tab 100mg |
| 97910989 | SODIUM VALPROATE ec tab 200mg |
| 97910988 | SODIUM VALPROATE ec tab 500mg |
| 97897992 | PHENYTOIN |
| 97896992 | PHENYTOIN SODIUM/ PHENOBARBITONE |
| 97884992 | PHENOBARBITONE & PHENYTOIN |
| 97883992 | PHENOBARBITONE/PHENYTOIN 50/100MG |
| 97867992 | PARAMETHADOINE |
| 97782998 | ACETAZOLAMIDE mr cap 250mg |
| 97779990 | CARBAMAZEPINE tabs 100mg |
| 97779989 | CARBAMAZEPINE tabs 200mg |
| 97779988 | CARBAMAZEPINE tabs 400mg |
| 97736998 | PHENYTOIN SODIUM tabs 50mg |
| 97736997 | PHENYTOIN SODIUM tabs 100mg |
| 97736992 | METHSUXIMIDE |
| 97721990 | SODIUM VALPROATE ec tab 200mg |
| 97721989 | SODIUM VALPROATE ec tab 500mg |
| 97697990 | SODIUM VALPROATE syrp 200mg/5ml |
| 97628998 | Valproic acid (as semisodium salt) ec tab 250mg 90 tablet(s) |
| 97628997 | Valproic acid (as semisodium salt) ec tab 500mg 90 tablet(s) |
| 97514998 | PHENYTOIN SODIUM caps 300mg |
| 97514997 | PHENYTOIN paed tab 50mg |
| 97511992 | PHENOBARBITONE |
| 97402992 | ETHOSUXIMIDE |
| 97401992 | ETHOTOIN |
| 97268998 | NITRAZEPAM caps 5mg |
| 97227998 | Nitrazepam tabs 5mg 28 tablet(s) |
| 97208998 | NITRAZEPAM tabs 5mg |
| 97207998 | NITRAZEPAM tabs 5mg |
| 97206998 | NITRAZEPAM tabs 5mg |
| 97205998 | NITRAZEPAM caps 5mg |
| 97203998 | Phenobarbital tabs 15mg 500 tablet(s) |
| 97203997 | Phenobarbital tabs 30mg 500 tablet(s) |
| 97203996 | Phenobarbital tabs 60mg 500 tablet(s) |
| 97202998 | Phenobarbital tabs 100mg 250 tablet(s) |
| 97185990 | ACETAZOLAMIDE syrp 40mg/ml |
| 97161992 | CLOBAZAM |
| 97160992 | CLOBAZAM |
| 97159992 | CLOBAZAM |
| 97158992 | CLOBAZAM |
| 97140990 | PHENYTOIN SODIUM tabs 50mg |
| 97140989 | PHENYTOIN SODIUM tabs 100mg |
| 97128990 | CARBAMAZEPINE mr tab 200mg |
| 97128989 | CARBAMAZEPINE mr tab 400mg |
| 97103990 | PHENOBARBITAL tabs 15mg |
| 97086998 | CARBAMAZEPINE mr tab 200mg |
| 97080998 | Phenobarbital sodium tabs 30mg 100 tablet(s) |
| 97080997 | Phenobarbital sodium tabs 60mg 500 tablet(s) |
| 97033998 | Carbamazepine tabs 100mg 56 tablet(s) |
| 97033997 | Carbamazepine tabs 200mg 56 tablet(s) |
| 97033996 | Carbamazepine tabs 400mg tablet(s) 28 |
| 96988998 | Acetazolamide tabs 250mg 112 tablet(s) |
| 96988997 | Acetazolamide pwdr 1 25g bottle |
| 96988996 | ACETAZOLAMIDE |
| 96987998 | Acetazolamide mr cap 500mg 30 capsule(s) |
| 96987997 | Acetazolamide mr cap 250mg 30 capsule(s) |
| 96986998 | Acetazolamide sodium inj 500mg 1 500mg vial(s) |
| 96986990 | SODIUM VALPROATE ec tab 200mg |
| 96986989 | SODIUM VALPROATE syrp 200mg/5ml |
| 96986988 | SODIUM VALPROATE ec tab 500mg |
| 96985998 | ACETAZOLAMIDE pwdr |
| 96978990 | PHENYTOIN SODIUM tabs 100mg |
| 96977990 | SODIUM VALPROATE ec tab 500mg |
| 96977989 | SODIUM VALPROATE ec tab 200mg |
| 96932990 | NITRAZEPAM tabs 5mg |
| 96916990 | CARBAMAZEPINE tabs 100mg |
| 96916989 | CARBAMAZEPINE tabs 200mg |
| 96916988 | CARBAMAZEPINE tabs 400mg |
| 96914998 | Beclamide tabs 500mg 1 tablet(s) |
| 96885998 | Carbamazepine liq 100mg/5ml 300 mls |
| 96866990 | PHENOBARBITAL tabs 100mg |
| 96850992 | PIRACETAM |
| 96817992 | METHSUXIMIDE |
| 96767998 | Ethosuximide caps 250mg 56 capsule(s) |
| 96767997 | Ethosuximide syrp 250mg/5ml 200 mls |
| 96740990 | PHENYTOIN SODIUM inj 250mg/5ml |
| 96697990 | CARBAMAZEPINE tabs 400mg |
| 96697989 | CARBAMAZEPINE tabs 100mg |
| 96697988 | CARBAMAZEPINE tabs 200mg |
| 96676990 | SODIUM VALPROATE crushable tab 100mg |
| 96648998 | Clobazam caps 10mg 30 capsule(s) |
| 96648997 | Clobazam tabs 10mg 30 tablet(s) |
| 96634998 | Clonazepam tabs 500 micrograms 100 tablet(s) |
| 96634997 | Clonazepam tabs 2mg 100 tablet(s) |
| 96634996 | Clonazepam conc soln inj 1mg/1ml 5 ampoule + wfi |
| 96571990 | CLONAZEPAM sf oral soln 2mg/5ml |
| 96536990 | CARBAMAZEPINE mr tab 200mg |
| 96536989 | CARBAMAZEPINE mr tab 400mg |
| 96479992 | CARBAMAZEPINE 100mg/5mL sf liq |
| 96463992 | Sod valproate c/r 200 mg tab 0 |
| 96446990 | CARBAMAZEPINE mr tab 200mg |
| 96446989 | CARBAMAZEPINE mr tab 400mg |
| 96386992 | PHENOBARBITONE/PHENYTOIN 60/100MG |
| 96160992 | CLOBAZAM |
| 96159990 | SODIUM VALPROATE sf liq 200mg/5ml |
| 96128990 | CARBAMAZEPINE mr tab 200mg |
| 96127990 | CARBAMAZEPINE mr tab 400mg |
| 96107990 | NITRAZEPAM tabs 5mg |
| 96096992 | PIRACETAM |
| 95995990 | NITRAZEPAM tabs 5mg |
| 95925990 | NITRAZEPAM tabs 5mg |
| 95899998 | MEPENZOLATE BROMIDE + PHENOBARBITAL tabs |
| 95852998 | Methylphenobarbital tabs 30mg 100 tablet(s) |
| 95852997 | Methylphenobarbital tabs 60mg 100 tablet(s) |
| 95852996 | Methylphenobarbital tabs 200mg 100 tablet(s) |
| 95838992 | PHENYTOIN SODIUM/ PHENOBARBITONE SODIUM |
| 95810990 | SODIUM VALPROATE syrp 200mg/5ml |
| 95750992 | ETHOSUXIMIDE |
| 95721998 | Nitrazepam caps 5mg 500 capsule(s) |
| 95720998 | Nitrazepam tabs 10mg 500 tablet(s) |
| 95720997 | Nitrazepam susp 2.5mg/5ml 150 mls |
| 95720996 | Nitrazepam susp 5mg/5ml 500 mls |
| 95719998 | NITRAZEPAM tabs 5mg |
| 95718998 | NITRAZEPAM tabs 10mg |
| 95690992 | TROXIDONE |
| 95598990 | NITRAZEPAM tabs 5mg |
| 95554998 | Phenobarbital sodium inj 200mg/ml 10 1ml ampoule(s) |
| 95553998 | PHENOBARBITAL SODIUM inj 200mg/ml |
| 95533998 | Phenytoin paed tab 50mg 112 tablet(s) |
| 95533997 | Phenytoin caps 25mg capsule(s) 28 |
| 95533996 | Phenytoin caps 50mg capsule(s) 28 |
| 95532998 | Phenytoin caps 100mg capsule(s) 84 |
| 95532997 | Phenytoin susp 30mg/5ml 500 mls |
| 95532996 | Phenytoin caps 300mg capsule(s) 28 |
| 95531998 | Phenytoin sodium inj 250mg/5ml 5 ampoule(s) |
| 95444998 | Lamotrigine tabs 50mg 42 tablets (add-on starter pack) |
| 95444997 | Lamotrigine tabs 100mg 56 tablet(s) |
| 95444996 | Lamotrigine tabs 25mg 42 tablet(s) |
| 95421992 | PHENOBARBITONE |
| 95420992 | PHENOBARBITONE |
| 95419992 | PHENOBARBITONE |
| 95418992 | PHENOBARBITONE |
| 95417992 | PHENOBARBITONE |
| 95415992 | Phenobarbitone sodium 15 mg tab 0 |
| 95411992 | PHENOBARBITONE |
| 95410992 | PHENOBARBITONE S/R |
| 95409992 | PHENOBARBITONE |
| 95404998 | LAMOTRIGINE tabs 50mg |
| 95404997 | LAMOTRIGINE tabs 100mg |
| 95404996 | LAMOTRIGINE tabs 25mg |
| 95403998 | Primidone tabs 250mg 100 tablet(s) |
| 95403997 | Primidone oral susp 250mg/5ml 1 mls |
| 95387992 | PARAMETHADOINE |
| 95361992 | SULTHIAME |
| 95330992 | NITRAZEPAM 5mg tablets |
| 95217990 | SODIUM VALPROATE + VALPROIC ACID mr tab 300mg |
| 95216990 | SODIUM VALPROATE + VALPROIC ACID mr tab 500mg |
| 95190990 | GABAPENTIN caps 100mg |
| 95189990 | GABAPENTIN caps 300mg |
| 95188990 | GABAPENTIN caps 400mg |
| 95187990 | GABAPENTIN tabs 600mg |
| 95186990 | GABAPENTIN tabs 800mg |
| 95161990 | GABAPENTIN caps 100mg |
| 95159990 | GABAPENTIN caps 300mg |
| 95158990 | GABAPENTIN caps 400mg |
| 95157990 | GABAPENTIN tabs 600mg |
| 95156990 | GABAPENTIN tabs 800mg |
| 94914992 | ACETAZOLAMIDE |
| 94835998 | Gabapentin caps 100mg 100 capsule(s) |
| 94835997 | Gabapentin caps 300mg 100 capsule(s) |
| 94835996 | Gabapentin caps 400mg 100 capsule(s) |
| 94834998 | GABAPENTIN caps 100mg |
| 94834997 | GABAPENTIN caps 300mg |
| 94834996 | GABAPENTIN caps 400mg |
| 94772990 | ACETAZOLAMIDE tabs 250mg |
| 94606998 | Sodium valproate ec tab 200mg 100 tablet(s) |
| 94606997 | Sodium valproate ec tab 500mg 100 tablet(s) |
| 94568998 | Sodium valproate crushable tab 100mg 100 tablet(s) |
| 94568997 | Sodium valproate sf liq 200mg/5ml 300 mls |
| 94568996 | Sodium valproate syrp 200mg/5ml 300 mls |
| 94525992 | PHENYTOIN |
| 94521992 | Phenobarbitone 30 mg cap 0 |
| 94520992 | PHENOBARBITONE |
| 94455992 | PHENYTOIN SODIUM/ PHENOBARBITONE |
| 94428992 | Mepenzolate bromide product |
| 94409998 | SODIUM VALPROATE ec tab 200mg |
| 94409997 | SODIUM VALPROATE ec tab 500mg |
| 94409996 | SODIUM VALPROATE crushable tab 100mg |
| 94408998 | SODIUM VALPROATE syrp 200mg/5ml |
| 94408997 | SODIUM VALPROATE sf liq 200mg/5ml |
| 94408996 | SODIUM VALPROATE pwdr/inj.soln 400mg |
| 94377998 | PHENOBARBITAL + BELLADONNA ALUMINIUM HYDROXIDE & MAGNESIUM TRISILICATE tabs |
| 94288992 | PHENYTOIN |
| 94285992 | PHENOBARBITONE |
| 94284992 | PHENOBARBITONE |
| 94283992 | PHENOBARBITONE |
| 94282992 | Phenobarbitone 15 mg cap 0 |
| 94281992 | PHENOBARBITONE SODIUM |
| 94279992 | PHENOBARBITONE |
| 94278992 | PHENOBARBITONE S/R |
| 94272992 | ETHOTOIN |
| 94256992 | SULTHIAME |
| 94167990 | GABAPENTIN caps 100mg |
| 94166990 | GABAPENTIN caps 300mg |
| 94165990 | GABAPENTIN caps 400mg |
| 94123990 | GABAPENTIN caps 100mg |
| 94122990 | GABAPENTIN caps 300mg |
| 94121990 | GABAPENTIN caps 400mg |
| 94120990 | LAMOTRIGINE tabs 25mg |
| 94119990 | LAMOTRIGINE tabs 50mg |
| 94118990 | LAMOTRIGINE tabs 100mg |
| 94117990 | LAMOTRIGINE tabs 200mg |
| 94116990 | GABAPENTIN caps 300mg |
| 94105990 | LAMOTRIGINE tabs 25mg |
| 94104990 | LAMOTRIGINE tabs 50mg |
| 94103990 | LAMOTRIGINE tabs 100mg |
| 94102990 | LAMOTRIGINE tabs 200mg |
| 94101990 | LAMOTRIGINE disp tab 25mg |
| 94100990 | LAMOTRIGINE disp tab 100mg |
| 94099990 | LAMOTRIGINE tabs 25mg |
| 94098990 | LAMOTRIGINE tabs 50mg |
| 94097990 | LAMOTRIGINE tabs 100mg |
| 94096990 | LAMOTRIGINE tabs 200mg |
| 94093990 | LAMOTRIGINE tabs 25mg |
| 94092990 | LAMOTRIGINE tabs 50mg |
| 94091990 | LAMOTRIGINE tabs 100mg |
| 94090990 | LAMOTRIGINE tabs 200mg |
| 94089990 | LAMOTRIGINE disp tab 5mg |
| 94088990 | LAMOTRIGINE disp tab 25mg |
| 94087990 | LAMOTRIGINE disp tab 100mg |
| 94086990 | LAMOTRIGINE tabs 25mg |
| 94085990 | LAMOTRIGINE tabs 50mg |
| 94084990 | LAMOTRIGINE tabs 100mg |
| 94083990 | LAMOTRIGINE tabs 200mg |
| 94068998 | VALPROIC ACID (AS SEMISODIUM SALT) ec tab 250mg |
| 94068997 | VALPROIC ACID (AS SEMISODIUM SALT) ec tab 500mg |
| 94066990 | LAMOTRIGINE tabs 25mg |
| 94065990 | LAMOTRIGINE tabs 50mg |
| 94064990 | LAMOTRIGINE tabs 100mg |
| 94063990 | LAMOTRIGINE tabs 200mg |
| 94049990 | LAMOTRIGINE tabs 25mg |
| 94048990 | LAMOTRIGINE tabs 50mg |
| 94047990 | LAMOTRIGINE tabs 100mg |
| 94046990 | LAMOTRIGINE tabs 200mg |
| 94045990 | LAMOTRIGINE disp tab 5mg |
| 94044990 | LAMOTRIGINE disp tab 25mg |
| 94043990 | LAMOTRIGINE disp tab 100mg |
| 94033992 | SULTHIAME |
| 94013990 | LAMOTRIGINE tabs 25mg |
| 94012990 | LAMOTRIGINE tabs 50mg |
| 94011990 | LAMOTRIGINE tabs 100mg |
| 94010990 | LAMOTRIGINE tabs 200mg |
| 94009990 | LAMOTRIGINE disp tab 25mg |
| 94008990 | LAMOTRIGINE disp tab 100mg |
| 93973990 | LAMOTRIGINE tabs 25mg |
| 93972990 | LAMOTRIGINE tabs 50mg |
| 93971990 | LAMOTRIGINE tabs 100mg |
| 93970990 | LAMOTRIGINE tabs 200mg |
| 93939990 | LAMOTRIGINE tabs 25mg |
| 93938990 | LAMOTRIGINE tabs 50mg |
| 93937990 | LAMOTRIGINE tabs 100mg |
| 93936990 | LAMOTRIGINE tabs 200mg |
| 93935990 | LAMOTRIGINE disp tab 5mg |
| 93934990 | LAMOTRIGINE disp tab 25mg |
| 93933990 | LAMOTRIGINE disp tab 100mg |
| 93913990 | CLONAZEPAM sf soln 500micrograms/5ml |
| 93894990 | NITRAZEPAM tabs 5mg |
| 93882990 | LAMOTRIGINE tabs 25mg |
| 93881990 | LAMOTRIGINE tabs 50mg |
| 93880990 | LAMOTRIGINE tabs 100mg |
| 93879990 | LAMOTRIGINE tabs 200mg |
| 93878990 | LAMOTRIGINE disp tab 5mg |
| 93877990 | LAMOTRIGINE disp tab 25mg |
| 93876990 | LAMOTRIGINE disp tab 100mg |
| 93812990 | GABAPENTIN caps 100mg |
| 93811990 | GABAPENTIN caps 300mg |
| 93810990 | GABAPENTIN caps 400mg |
| 93809990 | LAMOTRIGINE tabs 25mg |
| 93808990 | LAMOTRIGINE tabs 50mg |
| 93807990 | LAMOTRIGINE tabs 100mg |
| 93806990 | LAMOTRIGINE tabs 200mg |
| 93772990 | LAMOTRIGINE disp tab 100mg |
| 93771990 | LAMOTRIGINE disp tab 25mg |
| 93770998 | Vigabatrin tabs 500mg 100 tablet(s) |
| 93770997 | Vigabatrin sf pwdr 500mg 50 sachet(s) |
| 93770996 | VIGABATRIN |
| 93770990 | LAMOTRIGINE disp tab 2mg |
| 93769998 | VIGABATRIN tabs 500mg |
| 93769997 | VIGABATRIN sf pwdr 500mg |
| 93769990 | LAMOTRIGINE disp tab 5mg |
| 93768992 | PHENOBARBITONE |
| 93743990 | GABAPENTIN oral soln 250mg/5ml |
| 93727990 | CLOBAZAM oral susp 10mg/5ml |
| 93720992 | Phenobarbitone sodium 100 mg tab 0 |
| 93709990 | ACETAZOLAMIDE tabs 250mg |
| 93688990 | LAMOTRIGINE disp tab 25mg |
| 93687990 | LAMOTRIGINE disp tab 100mg |
| 93596990 | LAMOTRIGINE tabs 25mg |
| 93595990 | LAMOTRIGINE tabs 50mg |
| 93594990 | LAMOTRIGINE tabs 100mg |
| 93593990 | LAMOTRIGINE tabs 200mg |
| 93579998 | Carbamazepine mr tab 200mg 30 tablet(s) |
| 93579997 | Carbamazepine mr tab 400mg 30 tablet(s) |
| 93532998 | CARBAMAZEPINE mr tab 200mg |
| 93532997 | CARBAMAZEPINE mr tab 400mg |
| 93531998 | CARBAMAZEPINE chewable tab 100mg |
| 93531997 | CARBAMAZEPINE chewable tab 200mg |
| 93530998 | Carbamazepine chewable tab 100mg 56 tablet(s) |
| 93530997 | Carbamazepine chewable tab 200mg 56 tablet(s) |
| 93529990 | CLOBAZAM tabs 10mg |
| 93527990 | LAMOTRIGINE disp tab 5mg |
| 93512990 | LAMOTRIGINE disp tab 5mg |
| 93511990 | LAMOTRIGINE disp tab 25mg |
| 93501990 | LAMOTRIGINE disp tab 100mg |
| 93500990 | LAMOTRIGINE disp tab 50mg |
| 93493990 | LAMOTRIGINE tabs 25mg |
| 93492990 | LAMOTRIGINE tabs 50mg |
| 93491990 | LAMOTRIGINE tabs 100mg |
| 93490990 | LAMOTRIGINE tabs 200mg |
| 93460992 | LAMOTRIGINE 50mg tablets |
| 93454998 | Phenobarbital inj 15mg 1ml ampoule(s) 10 |
| 93454997 | Phenobarbital inj 30mg 1ml ampoule(s) 10 |
| 93454996 | Phenobarbital inj 60mg/ml 1ml ampoule(s) 10 |
| 93444990 | SODIUM VALPROATE ec tab 200mg |
| 93443990 | SODIUM VALPROATE ec tab 500mg |
| 93404992 | PHENOBARBITONE |
| 93224990 | ACETAZOLAMIDE oral susp 250mg/5ml |
| 93148998 | Sodium valproate pwdr/inj.soln 400mg 1 400mg vial + diluent |
| 93114990 | ETHOSUXIMIDE caps 250mg |
| 93064990 | LAMOTRIGINE disp tab 5mg |
| 93063990 | LAMOTRIGINE disp tab 25mg |
| 93062990 | LAMOTRIGINE disp tab 100mg |
| 93059998 | PIRACETAM FC tab 800mg |
| 93059997 | PIRACETAM FC tab 1200mg |
| 93059996 | PIRACETAM soln 33% |
| 93058998 | Piracetam fc tab 800mg 90 tablet(s) |
| 93058997 | Piracetam fc tab 1200mg 60 tablet(s) |
| 93058996 | Piracetam soln 33% 300 mls |
| 93051990 | GABAPENTIN caps 100mg |
| 93050990 | GABAPENTIN caps 400mg |
| 93049990 | LAMOTRIGINE disp tab 5mg |
| 93048990 | LAMOTRIGINE disp tab 25mg |
| 93047990 | LAMOTRIGINE disp tab 100mg |
| 93037992 | PHENOBARBITONE SODIUM ALCOHOL FREE |
| 93016998 | VALPROIC ACID ec soft gelatin ca 150mg |
| 93016997 | VALPROIC ACID ec soft gelatin ca 300mg |
| 93016996 | VALPROIC ACID ec soft gelatin ca 500mg |
| 93015998 | Valproic acid ec soft gelatin ca 150mg 100 capsule(s) |
| 93015997 | Valproic acid ec soft gelatin ca 300mg 100 capsule(s) |
| 93015996 | Valproic acid ec soft gelatin ca 500mg 100 capsule(s) |
| 93013990 | OXCARBAZEPINE tabs 150mg |
| 93012990 | OXCARBAZEPINE tabs 300mg |
| 93011990 | OXCARBAZEPINE tabs 600mg |
| 92973990 | CARBAMAZEPINE oral susp 500mg/5ml |
| 92969990 | GABAPENTIN caps 100mg |
| 92968990 | GABAPENTIN caps 300mg |
| 92918998 | SODIUM VALPROATE mr tab 200mg |
| 92918997 | SODIUM VALPROATE mr tab 300mg |
| 92918996 | SODIUM VALPROATE mr tab 500mg |
| 92917998 | Sodium valproate + valproic acid mr tab 200mg 100 tablet(s) |
| 92917997 | Sodium valproate + valproic acid mr tab 300mg 100 tablet(s) |
| 92917996 | Sodium valproate + valproic acid mr tab 500mg 100 tablet(s) |
| 92872990 | GABAPENTIN caps 100mg |
| 92871990 | GABAPENTIN caps 300mg |
| 92870990 | GABAPENTIN caps 400mg |
| 92837998 | CARBAMAZEPINE tabs 100mg |
| 92837997 | CARBAMAZEPINE tabs 200mg |
| 92837996 | CARBAMAZEPINE tabs 400mg |
| 92812998 | Phenytoin sf susp 90mg/5ml 500 mls |
| 92807990 | LAMOTRIGINE disp tab 100mg |
| 92806990 | LAMOTRIGINE disp tab 25mg |
| 92805990 | OXCARBAZEPINE tabs 150mg |
| 92804990 | OXCARBAZEPINE tabs 300mg |
| 92803990 | OXCARBAZEPINE tabs 600mg |
| 92802998 | SODIUM VALPROATE ec tab 200mg |
| 92802997 | SODIUM VALPROATE ec tab 500mg |
| 92802996 | SODIUM VALPROATE sf liq 200mg/5ml |
| 92797990 | CLONAZEPAM tabs 2mg |
| 92796990 | CLONAZEPAM tabs 500 micrograms |
| 92735998 | CARBAMAZEPINE supp 125mg |
| 92735997 | CARBAMAZEPINE supp 250mg |
| 92734998 | Carbamazepine supp 125mg 5 suppository(ies) |
| 92734997 | Carbamazepine supp 250mg 5 suppository(ies) |
| 92709998 | LAMOTRIGINE disp tab 5mg |
| 92709997 | LAMOTRIGINE disp tab 25mg |
| 92709996 | LAMOTRIGINE disp tab 100mg |
| 92701990 | PHENYTOIN SODIUM inj 250mg/5ml |
| 92700998 | Lamotrigine disp tab 5mg 28 tablet(s) |
| 92700997 | Lamotrigine disp tab 25mg 56 tablet(s) |
| 92700996 | Lamotrigine disp tab 100mg 56 tablet(s) |
| 92614990 | PHENYTOIN SODIUM tabs 100mg |
| 92378998 | CARBAMAZEPINE mr tab 400mg |
| 92375998 | LEVETIRACETAM tabs 250mg |
| 92375997 | LEVETIRACETAM tabs 500mg |
| 92375996 | LEVETIRACETAM tabs 1000mg |
| 92345998 | Sodium valproate mr tab 300mg 100 tablet(s) |
| 92283998 | CARBAMAZEPINE mr tab 200mg |
| 92282998 | CARBAMAZEPINE mr tab 400mg |
| 92131998 | CARBAMAZEPINE mr tab 200mg |
| 92131997 | CARBAMAZEPINE mr tab 400mg |
| 92064998 | FOSPHENYTOIN SODIUM conc for inj 750mg/10ml |
| 91839998 | Oxcarbazepine sf oral susp 60mg/ml 250 mls |
| 91643998 | ACETAZOLAMIDE SODIUM inj 500mg |
| 91626998 | OXCARBAZEPINE tabs 300mg |
| 91625998 | Oxcarbazepine tabs 150mg 50 tablet(s) |
| 91625997 | Oxcarbazepine tabs 300mg 50 tablet(s) |
| 91625996 | Oxcarbazepine tabs 600mg 50 tablet(s) |
| 91596998 | LAMOTRIGINE tabs 200mg |
| 91596997 | LAMOTRIGINE disp tab 2mg |
| 91465998 | Lamotrigine tabs 200mg 30 tablet(s) |
| 91465997 | Lamotrigine disp tab 2mg 30 tablet(s) |
| 91218998 | OXCARBAZEPINE sf oral susp 60mg/ml |
| 91051998 | TOPIRAMATE tabs 50mg |
| 91051997 | TOPIRAMATE tabs 100mg |
| 91051996 | TOPIRAMATE tabs 200mg |
| 91050998 | Topiramate tabs 50mg 60 tablet(s) |
| 91050997 | Topiramate tabs 100mg 60 tablet(s) |
| 91050996 | Topiramate tabs 200mg 60 tablet(s) |
| 91045998 | TOPIRAMATE tabs 25mg |
| 91044998 | Topiramate tabs 25mg 60 tablet(s) |
| 91044997 | Topiramate caps 15mg 60 capsule(s) |
| 91044996 | Topiramate caps 25mg 60 capsule(s) |
| 90904998 | PHENYTOIN+ PHENOBARBITAL |
| 90858998 | TIAGABINE tabs 15mg |
| 90780998 | Phenytoin sodium caps 25mg 28 capsule(s) |
| 90780997 | Phenytoin sodium caps 50mg 28 capsule(s) |
| 90780996 | Phenytoin sodium caps 100mg 84 capsule(s) |
| 90776998 | Phenytoin sodium caps 300mg 28 capsule(s) |
| 90505998 | Sodium valproate mr tab 500mg 100 tablet(s) |
| 90426998 | GABAPENTIN tabs 600mg |
| 90426997 | GABAPENTIN tabs 800mg |
| 90425998 | Gabapentin caps & tabs 300mg + 600mg 1 titration pack (40 x 300mg caps + 10 x 600mg tabs) |
| 90424998 | GABAPENTIN caps & tabs 300mg + 600mg |
| 89991998 | Fosphenytoin sodium conc for inj 75mg/ml 10 10ml vial(s) |
| 89409998 | Tiagabine tabs 5mg 100 tablet(s) |
| 89409997 | Tiagabine tabs 10mg 100 tablet(s) |
| 89409996 | Tiagabine tabs 15mg 100 tablet(s) |
| 89408998 | TIAGABINE tabs 5mg |
| 89408997 | TIAGABINE tabs 10mg |
| 89408996 | TIAGABINE tabs 15mg |
| 89384998 | CARBAMAZEPINE mr tab 200mg |
| 89384997 | CARBAMAZEPINE mr tab 400mg |
| 89231998 | OXCARBAZEPINE tabs 600mg |
| 89210998 | Levetiracetam tabs 250mg 60 tablet(s) |
| 89210997 | Levetiracetam tabs 500mg 60 tablet(s) |
| 89210996 | Levetiracetam tabs 1000mg 60 tablet(s) |
| 89008998 | ACETAZOLAMIDE mr cap 250mg |
| 88868998 | TOPIRAMATE caps 15mg |
| 88868997 | TOPIRAMATE caps 25mg |
| 88868996 | TOPIRAMATE caps 50mg |
| 88423998 | CLONAZEPAM |
| 88423997 | Clonazepam susp 500micrograms/5ml 150 mls |
| 88423996 | Clonazepam sf oral soln 2mg/5ml 150 mls |
| 88422998 | CLONAZEPAM oral drops 2.5mg/ml |
| 88396998 | Topiramate caps 50mg 60 capsule(s) |
| 88217998 | CARBAMAZEPINE mr tab 200mg |
| 88217997 | CARBAMAZEPINE mr tab 400mg |
| 88178998 | SODIUM VALPROATE mr tab 500mg |
| 88177998 | SODIUM VALPROATE mr tab 300mg |
| 87522998 | PHENOBARBITAL SODIUM |
| 87408998 | Pregabalin caps 25mg 56 capsule(s) |
| 87407998 | Pregabalin caps 50mg 84 capsule(s) |
| 87406998 | Pregabalin caps 75mg 56 capsule(s) |
| 87405998 | Pregabalin caps 100mg 84 capsule(s) |
| 87404998 | Pregabalin caps 150mg 56 capsule(s) |
| 87403998 | Pregabalin caps 200mg 84 capsule(s) |
| 87402998 | Pregabalin caps 300mg 56 capsule(s) |
| 87401998 | PREGABALIN caps 25mg |
| 87400998 | PREGABALIN caps 50mg |
| 87399998 | PREGABALIN caps 75mg |
| 87398998 | PREGABALIN caps 100mg |
| 87397998 | PREGABALIN caps 150mg |
| 87396998 | PREGABALIN caps 200mg |
| 87395998 | PREGABALIN caps 300mg |
| 87289998 | NITRAZEPAM tabs 5mg |
| 87196998 | Levetiracetam tabs 750mg 60 tablet(s) |
| 87195998 | Levetiracetam oral soln 100mg/ml 300 mls |
| 87194998 | LEVETIRACETAM tabs 750mg |
| 87193998 | LEVETIRACETAM oral soln 100mg/ml |
| 87191998 | PHENOBARBITAL |
| 87106998 | PRIMIDONE tabs 250mg |
| 87030998 | PHENOBARBITAL |
| 86846998 | Zonisamide caps 25mg 14 capsule(s) |
| 86845998 | Zonisamide caps 50mg 56 capsule(s) |
| 86844998 | Zonisamide caps 100mg 56 capsule(s) |
| 86843998 | ZONISAMIDE caps 25mg |
| 86842998 | ZONISAMIDE caps 50mg |
| 86841998 | ZONISAMIDE caps 100mg |
| 86604998 | Clonazepam sf soln 500micrograms/5ml mls 150 |
| 86485998 | GABAPENTIN |
| 86457998 | CLOBAZAM |
| 86429998 | ACETAZOLAMIDE |
| 86362998 | GABAPENTIN |
| 86349998 | PRIMIDONE |
| 86161998 | CLOBAZAM |
| 86109998 | ETHOSUXIMIDE caps 250mg |
| 86019998 | Lamotrigine disp tab 50mg tablet(s) 56 |
| 85969998 | Levetiracetam conc soln inf 500mg/5ml 10 vial(s) |
| 85968998 | LEVETIRACETAM conc soln inf 500mg/5ml |
| 85954998 | ETHOSUXIMIDE syrp 250mg/5ml |
| 85786998 | Acetazolamide oral liq |
| 85753998 | Gabapentin oral liq |
| 85633998 | Clonazepam oral liq |
| 85559998 | CLONAZEPAM |
| 85557998 | Phenytoin oral liq |
| 85534998 | Primidone caps |
| 85533998 | Primidone oral liq |
| 85486998 | ACETAZOLAMIDE |
| 85466998 | PRIMIDONE |
| 85424998 | Clobazam oral liq |
| 85423998 | Clobazam caps capsule(s) 1 |
| 85379998 | Lamotrigine (ipu) disp tab 200mg |
| 85235998 | Phenobarbital oral liq |
| 85180998 | PRIMIDONE |
| 85159998 | Carbamazepine oral liq |
| 85030998 | Sodium valproate inj 300mg/3ml 5 ampoule(s) |
| 85029998 | SODIUM VALPROATE inj 300mg/3ml |
| 84953998 | Levetiracetam oral liq mls 1 |
| 84903998 | Lamotrigine oral liq |
| 84729998 | Topiramate oral liq |
| 84720998 | Sodium valproate supp |
| 84671998 | Sodium valproate mr cap 150mg 100 capsule(s) |
| 84670998 | Sodium valproate mr cap 300mg 100 capsule(s) |
| 84669998 | Sodium valproate mr granules 500mg sachet(s) 100 |
| 84668998 | Sodium valproate mr granules 1000mg sachet(s) 100 |
| 84667998 | SODIUM VALPROATE mr cap 150mg |
| 84666998 | SODIUM VALPROATE mr cap 300mg |
| 84665998 | SODIUM VALPROATE MR granules 500mg |
| 84664998 | SODIUM VALPROATE MR granules 1000mg |
| 84420998 | Rufinamide fc tab 100mg |
| 84419998 | Rufinamide fc tab 200mg |
| 84418998 | Rufinamide fc tab 400mg |
| 84417998 | RUFINAMIDE FC tab 100mg |
| 84416998 | RUFINAMIDE FC tab 200mg |
| 84415998 | RUFINAMIDE FC tab 400mg |
| 84311998 | CARBAMAZEPINE |
| 84234998 | Pregabalin caps 225mg capsule(s) 56 |
| 84233998 | PREGABALIN caps 225mg |
| 84200998 | Nitrazepam oral liq |
| 84194998 | Phenobarbital caps |
| 84193998 | Phenobarbital tabs |
| 84127998 | PRIMIDONE |
| 84098998 | Stiripentol caps 250mg capsule(s) 60 |
| 84097998 | Stiripentol caps 500mg capsule(s) 60 |
| 84096998 | Stiripentol sach 250mg sachet(s) 60 |
| 84095998 | Stiripentol sach 500mg sachet(s) 60 |
| 84094998 | STIRIPENTOL caps 250mg |
| 84093998 | STIRIPENTOL caps 500mg |
| 84092998 | STIRIPENTOL sach 250mg |
| 84091998 | STIRIPENTOL sach 500mg |
| 84089998 | Sodium valproate inj 1000mg/10ml ampoule(s) 5 |
| 84088998 | SODIUM VALPROATE inj 1000mg/10ml |
| 83946998 | Oxcarbazepine oral liq mls 1 |
| 83896998 | Phenytoin sodium oral liq mls 1 |
| 83895998 | Phenobarbital sodium oral liq mls 1 |
| 83797998 | Vigabatrin oral liq |
| 83794998 | Sodium valproate + valproic acid mr granules 100mg |
| 83793998 | Sodium valproate + valproic acid mr granules 250mg |
| 83792998 | Sodium valproate + valproic acid mr granules 500mg |
| 83791998 | Sodium valproate + valproic acid mr granules 750mg |
| 83790998 | Sodium valproate + valproic acid (ipu) mr granules 1000mg |
| 83766998 | Sodium valproate oral liq mls 1 |
| 83709998 | SODIUM VALPROATE + VALPROIC ACID MR |
| 83708998 | SODIUM VALPROATE + VALPROIC ACID MR granules 50mg |
| 83707998 | SODIUM VALPROATE + VALPROIC ACID MR granules 100mg |
| 83706998 | SODIUM VALPROATE + VALPROIC ACID MR granules 250mg |
| 83705998 | SODIUM VALPROATE + VALPROIC ACID MR granules 500mg |
| 83704998 | SODIUM VALPROATE + VALPROIC ACID MR granules 750mg |
| 83518998 | Lacosamide soln for inf 200mg/20ml vial(s) 1 |
| 83517998 | LACOSAMIDE soln for inf 200mg/20ml |
| 83516998 | Lacosamide syrp 15mg/ml mls 200 |
| 83515998 | LACOSAMIDE syrp 15mg/ml |
| 83514998 | Lacosamide tabs 100mg tablet(s) 56 |
| 83513998 | LACOSAMIDE tabs 100mg |
| 83512998 | Lacosamide tabs 150mg tablet(s) 56 |
| 83511998 | LACOSAMIDE tabs 150mg |
| 83510998 | Lacosamide tabs 200mg tablet(s) 56 |
| 83509998 | LACOSAMIDE tabs 200mg |
| 83508998 | Lacosamide tabs 50mg tablet(s) 14 |
| 83507998 | LACOSAMIDE tabs 50mg |
| 83480998 | SODIUM VALPROATE ec tab 200mg |
| 83479998 | SODIUM VALPROATE ec tab 500mg |
| 83321998 | Sodium valproate mr tab 200mg tablet(s) 100 |

Read Codes for Symptoms of Epilepsy

| Code | Description |
| --- | --- |
| R003400 | [D]Nocturnal seizure |
| 1B63.00 | Had a fit |
| 1B63.11 | Fit - had one symptom |
| 1B64.00 | Had a convulsion |
| 1B64.11 | Convulsion - symptom |
| 282..00 | O/E - fit/convulsion |
| 282..11 | O/E - a convulsion |
| 282..12 | O/E - a fit |
| 282..13 | O/E - a seizure |
| 2822.00 | O/E - grand mal fit |
| 2823.00 | O/E - petit mal fit |
| 2824.00 | O/E - focal (Jacksonian) fit |
| 2824.11 | O/E - Jacksonian fit |
| 2824.12 | O/E - focal fit |
| 2825.00 | O/E - psychomotor fit |
| 2826.00 | O/E - salaam attack |
| 2828.00 | Absence seizure |
| 282Z.00 | O/E - fit/convulsion NOS |
| 667Q.00 | 1 to 12 seizures a year |
| 667R.00 | 2 to 4 seizures a month |
| 667S.00 | 1 to 7 seizures a week |
| 667T.00 | Daily seizures |
| 667V.00 | Many seizures a day |
| Eu10800 | [X]Alcohol withdrawal-induced seizure |
| F132300 | Myoclonic jerks |
| F132z12 | Myoclonic seizure |
| F250011 | Epileptic absences |
| F250200 | Epileptic seizures - atonic |
| F250300 | Epileptic seizures - akinetic |
| F251200 | Epileptic seizures - clonic |
| F251300 | Epileptic seizures - myoclonic |
| F251400 | Epileptic seizures - tonic |
| F251600 | Grand mal seizure |
| F252.00 | Petit mal status |
| F253.00 | Grand mal status |
| F253.11 | Status epilepticus |
| F254500 | Complex partial epileptic seizure |
| F255600 | Simple partial epileptic seizure |
| F256100 | Salaam attacks |
| F258.00 | Post-ictal state |
| F25X.00 | Status epilepticus unspecified |
| F25y300 | Complex partial status epilepticus |
| F25z.11 | Fit (in known epileptic) NOS |
| Fyu5200 | [X]Other status epilepticus |
| Fyu5900 | [X]Status epilepticus unspecified |
| Q480.00 | Convulsions in newborn |
| Q480.11 | Fits in newborn |
| Q480.12 | Seizures in newborn |
| R003.00 | [D]Convulsions |
| R003100 | [D]Convulsions infantile |
| R003200 | [D]Fit |
| R003211 | [D]Fit (in non epileptic) NOS |
| R003y00 | [D]Other specified convulsion |
| R003z00 | [D]Convulsion NOS |
| R003z11 | [D]Seizure NOS |
| Ryu7100 | [X]Other and unspecified convulsions |
