# Supplementary material for: Association between Antibiotic Prescribing in Pregnancy and Cerebral Palsy or Epilepsy in Children Born at Term: A Cohort Study Using The Health Improvement Network
Source: PLoS One. 2015 Mar 25;10(3):e0122034. doi: 10.1371/journal.pone.0122034 (PMC4373729; doi:10.1371/journal.pone.0122034)
Supplement: S3 Appendix — (DOCX) [file pone.0122034.s003.docx]

**S3 Appendix – Description of algorithm and codes used to identify children with cerebral palsy.**

Code lists below were created by Wilhelmine Meeraus with expert advice and input from: Prof Ruth Gilbert (paediatrician, epidemiologist); Dr Irene Petersen (statistician, primary care data epidemiologist); and Prof Irwin Nazareth (GP, epidemiologist).

A child with possible cerebral palsy was defined as someone with one or more codes in their medical record indicative of a diagnosis or management or treatment of symptoms of cerebral palsy. The full medical records of all children with possible cerebral palsy were reviewed for evidence of a non-progressive motor-disability consistent with cerebral palsy. All reviewers were blinded as to whether the children’s mothers were prescribed antibiotics in pregnancy. Six out of ten (59%) of children initially identified with cerebral palsy (based purely on codes) had no consistent evidence of cerebral palsy – these children were not considered as having cerebral palsy in this study. The most frequent codes not associated with cerebral palsy were: spasmodic torticollis, hypotonia, talipes /clubfoot, tenotomy, gastrostomy.

Read Codes for Cerebral Palsy Diagnosis

| **Code** | **Description** |
| --- | --- |
| F137.11 | Athetoid cerebral palsy |
| F137.12 | Athetosis - congenital |
| F137.13 | Vogt's disease |
| F137000 | Athetoid cerebral palsy |
| F137011 | Vogt's disease |
| F137100 | Double athetosis |
| F137111 | Congenital athetosis |
| F23..00 | Congenital cerebral palsy |
| F23..11 | Congenital spastic cerebral palsy |
| F23..12 | Infantile cerebral palsy |
| F23..13 | Littles disease |
| F23..14 | Cerebral atonia |
| F230.00 | Congenital diplegia |
| F230.11 | Paraplegia - congenital |
| F230000 | Congenital paraplegia |
| F230100 | Cerebral palsy with spastic diplegia |
| F230z00 | Congenital diplegia NOS |
| F231.00 | Congenital hemiplegia |
| F232.00 | Congenital quadriplegia |
| F232.11 | Tetraplegia - congenital |
| F233.00 | Congenital monoplegia |
| F233.11 | Congenital spastic foot |
| F234.00 | Infantile hemiplegia NOS |
| F23y.00 | Other congenital cerebral palsy |
| F23y000 | Ataxic infantile cerebral palsy |
| F23y100 | Flaccid infantile cerebral palsy |
| F23y200 | Spastic cerebral palsy |
| F23y300 | Dyskinetic cerebral palsy |
| F23y400 | Ataxic diplegic cerebral palsy |
| F23y500 | Worster-Drought syndrome |
| F23y511 | Congenital suprabulbar paresis |
| F23yz00 | Other infantile cerebral palsy NOS |
| F23z.00 | Congenital cerebral palsy NOS |
| Fyu9.00 | [X]Cerebral palsy and other paralytic syndromes |
| Fyu9000 | [X]Other infantile cerebral palsy |
| G669.00 | Cerebral palsy, not congenital or infantile, acute |

Read Codes for Management of Cerebral Palsy

| **Code** | **Description** |
| --- | --- |
| 7048100 | Rhizotomy of spinal nerve root |
| 7617.00 | Gastrostomy operations |
| 7617.11 | Artificial opening to stomach |
| 7617.12 | Creation of gastrostomy |
| 7617000 | Creation of permanent gastrostomy |
| 7617100 | Creation of temporary gastrostomy |
| 7617111 | Creation of gastrostomy NEC |
| 7617112 | Creation of Witzel temporary gastrostomy |
| 7617200 | Reconstruction of gastrostomy |
| 7617300 | Closure of gastrostomy |
| 7617400 | Attention to gastrostomy tube |
| 7617500 | Removal of gastrostomy tube |
| 7617600 | Change of gastrostomy tube |
| 7617y00 | Other specified gastrostomy operation |
| 7617z00 | Gastrostomy operation NOS |
| 761E.00 | Other therapeutic fibreoptic endoscopy on upper GI tract |
| 761E.11 | Other therapeutic gastroscopy |
| 761E000 | Fibreoptic endoscopic insertion prosthesis in upper GI tract |
| 761E100 | Fibreoptic endoscopic removal of FB from upper GI tract |
| 761E200 | Fibreoptic endoscopic dilation of upper GI tract |
| 761E211 | Fibreoptic dilation of oesophagus |
| 761E300 | Temporary percutaneous endoscopic gastrostomy |
| 761E400 | Permanent percutaneous endoscopic gastrostomy |
| 761E500 | Fibreop endoscop reduction intussusception gastroenterostomy |
| 761E600 | Fibreoptic endoscopic percutaneous insert gastrostomy (PEG) |
| 761E700 | Fib end pressure-controlled balloon dilat low oesoph sphinc |
| 761E800 | Fibreoptic endoscopic dilation of upper GI tract NEC |
| 761E900 | Fibreoptic endoscopic removal of gastrostomy tube |
| 761EA00 | Fibreoptic endoscopic percutaneous insertion of gastrostomy |
| 761Ey00 | Other fibreoptic therapeutic endoscopy on upper GI tract OS |
| 761Ez00 | Other fibreoptic therapeutic endoscopy on upper GI tract NOS |
| 7811011 | Cholecystogastrostomy |
| 7H46.00 | Adjustment to length of tendon |
| 7H46000 | Subcutaneous tenotomy |
| 7H46100 | Tenotomy NEC |
| 7H46400 | Lengthening of tendon |
| 7H46411 | Lengthening of muscle |
| 7H46500 | Lengthening of tendo achilles |
| 7H46511 | Lengthening of tendo calcaneus |
| 7H46512 | Poncet lengthening of achilles tendon |
| 7H46513 | Vulpius elongation of tendo achilles |
| 7H46514 | White lengthening of tendo calcaneus |
| 7H46600 | Distal recession of gastrocnemius |
| 7H46611 | Strayer distal recession of gastrocnemius |
| 7H46y00 | Other specified adjustment to length of tendon |
| 7H46y11 | Duvries tenoplasty |
| 7H46y12 | Lipscomb tenoplasty |
| 7H46y13 | Other specified tenoplasty |
| 7H46z00 | Adjustment to length of tendon NOS |
| 7L18800 | Intramuscular infiltration of botulinum toxin |
| 8CJ2.00 | Percutaneous endoscopic gastrostomy feeding |
| 8D69.00 | Gastrostomy aid training |
| 8D6A.00 | Gastrostomy bag fitting |
| 8D6B.00 | Gastrostomy bag adjustment |
| 8D6C.00 | Gastrostomy bag changed |
| 8E54.00 | Exercises for spasticity |
| J524100 | Complication of gastrostomy |
| SLF2.00 | Skeletal muscle relaxant poisoning |
| SP06900 | Infected percutaneous endoscopic gastrostomy site |
| TB03300 | Formation of gastrostomy with complication, without blame |
| TJF2.00 | Adverse reaction to skeletal muscle relaxants |
| TJF2z00 | Adverse reaction to skeletal muscle relaxant NOS |
| U60F111 | [X] Adverse reaction to skeletal muscle relaxants |
| U60F117 | [X] Adverse reaction to skeletal muscle relaxant NOS |
| ZC32.54 | PEG - Percutaneous endoscopic gastrostomy feeding |
| ZC65200 | Gastrostomy feeding |
| ZC65300 | Percutaneous endoscopic gastrostomy feeding |
| ZC65311 | PEG - Percutaneous endoscopic gastrostomy feeding |
| ZC65400 | Button gastrostomy feeding |
| ZV44100 | [V]Has gastrostomy |
| ZV55100 | [V]Attention to gastrostomy |

Encrypted Mulitlex Codes for Treatment used in Cerebral Palsy

*NB. Encrypted multiplex codes were were used in The Health Improvement Network (THIN) to classify medicinal products and devices prior to their switch to Gemscript codes.*

| **Code** | **Description** |
| --- | --- |
| 84060998 | BOTULINUM A TOXIN inj 500 units |
| 84084998 | BOTULINUM A TOXIN pwdr/inj.soln 50 units/vial |
| 84117998 | DANTROLENE oral soln 12.5mg/5ml |
| 84847998 | TIZANIDINE HCl oral susp 2mg/5ml |
| 85180998 | PRIMIDONE oral susp 50mg/5ml |
| 85308998 | TETRABENAZINE oral susp 25mg/5ml |
| 85984998 | BOTULINUM B TOXIN inj 10000 units/2ml |
| 86030998 | DANTROLENE oral susp 10mg/5ml |
| 86112998 | BOTULINUM A TOXIN pwdr/inj.soln 50 units/vial |
| 86302998 | DANTROLENE oral susp 25mg/5ml |
| 87316998 | BACLOFEN sf oral soln 5mg/5ml |
| 88475997 | TIZANIDINE HCl tabs 4mg |
| 88475998 | TIZANIDINE HCl tabs 2mg |
| 88477997 | TIZANIDINE HCl tabs 4mg |
| 88477998 | TIZANIDINE HCl tabs 2mg |
| 89833998 | RILUZOLE tabs 50mg |
| 89834998 | RILUZOLE tabs 50mg |
| 90912998 | TETRABENAZINE tabs 25mg |
| 91176998 | BACLOFEN tabs 10mg |
| 91597996 | BACLOFEN intrathec inj 10mg/20ml |
| 91597997 | BACLOFEN intrathec inj 10mg/5ml |
| 91597998 | BACLOFEN intrathec inj 50micrograms/1ml |
| 91598996 | BACLOFEN intrathec inj 10mg/20ml |
| 91598997 | BACLOFEN intrathec inj 10mg/5ml |
| 92080998 | BOTULINUM B TOXIN inj 5000 units/ml |
| 92667998 | BOTULINUM A TOXIN pwdr/inj.soln 100 units/vial |
| 93058996 | PIRACETAM soln 33% |
| 93058997 | PIRACETAM FC tab 1200mg |
| 93058998 | PIRACETAM FC tab 800mg |
| 93059996 | PIRACETAM soln 33% |
| 93059997 | PIRACETAM FC tab 1200mg |
| 93059998 | PIRACETAM FC tab 800mg |
| 93519990 | BACLOFEN sf oral soln 5mg/5ml |
| 93698998 | ORPHENADRINE CITRATE tabs 100mg |
| 94254992 | ORPHENADRINE CITRATE S/R 100 MG TAB |
| 94693992 | BACLOFEN 2.5 MG CAP |
| 94867990 | TIZANIDINE HCl tabs 2mg |
| 94947990 | TIZANIDINE HCl tabs 2mg |
| 95199997 | TETRABENAZINE sf susp 50mg/5ml |
| 95199998 | TETRABENAZINE tabs 25mg |
| 95242990 | HALOPERIDOL tabs 500micrograms |
| 95672998 | ORPHENADRINE CITRATE inj 30mg/ml |
| 95875997 | METHOCARBAMOL inj 100mg/ml |
| 95875998 | METHOCARBAMOL tabs 750mg |
| 96096992 | PIRACETAM 400 MG CAP |
| 96460997 | DANTROLENE caps 100mg |
| 96460998 | DANTROLENE caps 25mg |
| 96725992 | ORPHENADRINE HCl 100 MG TAB |
| 96787998 | BACLOFEN tabs 10mg |
| 96863997 | CARISOPRODOL tabs 350mg |
| 96863998 | CARISOPRODOL tabs 125mg |
| 96864997 | CARISOPRODOL tabs 350mg |
| 96864998 | CARISOPRODOL tabs 125mg |
| 96917997 | BACLOFEN sf oral soln 5mg/5ml |
| 96917998 | BACLOFEN tabs 10mg |
| 97784990 | BACLOFEN tabs 10mg |
| 98210990 | BACLOFEN tabs 10mg |
| 98355990 | BACLOFEN tabs 10mg |
| 98532998 | TETRABENAZINE tabs 25mg |
| 98539998 | METHOCARBAMOL inj 100mg/ml |
| 98540998 | METHOCARBAMOL tabs 750mg |
| 98861998 | BOTULINUM A TOXIN inj 500 units |
| 99344998 | ORPHENADRINE CITRATE tabs 100mg |
| 99468997 | BACLOFEN sf oral soln 5mg/5ml |
| 99468998 | BACLOFEN tabs 10mg |
| 99543997 | BOTULINUM A TOXIN pwdr/inj.soln 100 units/vial |
| 99543998 | BOTULINUM A TOXIN inj 500 units |
| 99750992 | BACLOFEN sach 3 MG |
| 99788997 | DANTROLENE caps 100mg |
| 99788998 | DANTROLENE caps 25mg |
| 99793990 | BACLOFEN tabs 10mg |
| 99795990 | BACLOFEN tabs 10mg |
| 99796990 | BACLOFEN tabs 10mg |
| 82447998 | BOTULINUM A TOXIN pwdr/inj.soln 200units/vial |
| 82744998 | BOTULINUM A TOXIN pwdr/inj.soln 125 units/vial |
| 82745998 | BOTULINUM A TOXIN pwdr/inj.soln 125 units/vial |
| 84016998 | BOTULINUM A TOXIN pwdr/inj.soln 100 units/vial |
| 84116998 | DANTROLENE oral susp 12.5mg/5ml |
| 84810998 | QUININE BISULPHATE oral liq |
| 84828998 | TIZANIDINE HCl oral liq |
| 84846998 | TIZANIDINE HCl oral susp 4mg/5ml |
| 85033998 | BACLOFEN oral liq |
| 85728998 | DANTROLENE oral liq |
| 85982998 | BOTULINUM B TOXIN inj 2500 units/0.5ml |
| 85983998 | BOTULINUM B TOXIN inj 5000 units/1ml |
| 85986998 | BOTULINUM B TOXIN inj 2500 units/0.5ml |
| 85987998 | BOTULINUM B TOXIN inj 5000 units/1ml |
| 85988998 | BOTULINUM B TOXIN inj 10000 units/2ml |
| 86111998 | BOTULINUM A TOXIN pwdr/inj.soln 50 units/vial |
| 86428998 | DANTROLENE oral susp 100mg/5ml |
| 88514998 | BACLOFEN tabs 10mg |
| 90751998 | BOTULINUM B TOXIN inj 5000 units/ml |
| 91598998 | BACLOFEN intrathec inj 50micrograms/1ml |
| 92217990 | TIZANIDINE HCl tabs 4mg |
| 92218990 | TIZANIDINE HCl tabs 2mg |
| 93081990 | TIZANIDINE HCl tabs 4mg |
| 93082990 | TIZANIDINE HCl tabs 2mg |
| 93313990 | BACLOFEN sf oral soln 5mg/5ml |
| 93456990 | TIZANIDINE HCl tabs 4mg |
| 93457990 | TIZANIDINE HCl tabs 2mg |
| 93470990 | BACLOFEN sf oral soln 5mg/5ml |
| 94069990 | BACLOFEN tabs 10mg |
| 94866990 | TIZANIDINE HCl tabs 4mg |
| 94946990 | TIZANIDINE HCl tabs 4mg |
| 95251990 | BACLOFEN tabs 10mg |
| 95671998 | ORPHENADRINE CITRATE + PARACETAMOL tabs |
| 96381990 | BACLOFEN tabs 10mg |
| 96850992 | NOOTROPIL 400 MG CAP |
| 96920990 | BACLOFEN tabs 10mg |
| 97191990 | TETRABENAZINE tabs 25mg |
| 97953990 | TETRABENAZINE tabs 25mg |
| 98068990 | TETRABENAZINE sf susp 50mg/5ml |
| 98209990 | BACLOFEN tabs 10mg |
| 99344997 | ORPHENADRINE CITRATE inj 30mg/ml |
| 99794990 | BACLOFEN tabs 10mg |

Read codes for Symptoms of Cerebral Palsy

| **Code** | **Description** |
| --- | --- |
| 2833.00 | O/E - hemiplegia |
| 2834.00 | O/E - monoplegia |
| 2836.00 | O/E - quadriplegia |
| 2837.00 | O/E - diplegia |
| 294..11 | O/E - rigid muscle |
| 2942.00 | O/E - muscle tone hypertonic |
| 2943.00 | O/E - muscle rigid-clasp knife |
| 2944.00 | O/E - muscle rigid - cogwheel |
| 2944.11 | O/E - cog wheel rigidity |
| 2945.00 | O/E - muscle tone hypotonic |
| 2948.00 | O/E - muscle tone spastic |
| 2949.00 | Lower limb spasticity |
| 294A.00 | Upper limb spasticity |
| 296..00 | O/E - muscle contracture |
| 2962.00 | O/E - muscle contraction |
| 2963.00 | O/E - extension contracture |
| 2964.00 | O/E - flexion contracture |
| 296Z.00 | O/E - muscle contracture NOS |
| 2992.00 | O/E - gait spastic |
| 2992.11 | O/E - spastic gait |
| 2993.00 | O/E - gait ataxic |
| 2993.11 | O/E - ataxic gait |
| 2999.00 | Toe-walking gait |
| 29L..12 | O/E - ataxia |
| 29L2.00 | O/E - arms ataxic |
| 29L3.00 | O/E - legs ataxic |
| 7H54.00 | Release of contracture of muscle |
| 7H54000 | Quadricepsplasty |
| 7H54011 | Judet quadricepsplasty |
| 7H54012 | Thompson quadricepsplasty |
| 7H54100 | Excision of Volkmann contracture of forearm |
| 7H54200 | Release of paralytic tether |
| 7H54300 | Release of cicatricial tether |
| 7H54400 | Release of sternomastoid muscle |
| 7H54500 | Release of Finochetti contracture of hand |
| 7H54600 | Release of intrinsic muscle of hand |
| 7H54700 | Release intrinsic muscle of thumb |
| 7H54y00 | Other specified release of contracture of muscle |
| 7H54z00 | Release of contracture of muscle NOS |
| 7K6T.00 | Release of contracture of joint |
| 7K6T000 | Release of webbing of neck |
| 7K6T100 | Release of torticollis |
| 7K6T200 | Release of contracture of shoulder joint |
| 7K6T300 | Release of contracture of hip joint |
| 7K6T400 | Release of contracture of knee joint |
| 7K6T411 | Yount soft tissue release of knee |
| 7K6T412 | Lateral release of contracture of knee joint |
| 7K6T500 | Limited release of contracture of capsule of joint |
| 7K6T600 | Release of contracture of fingers |
| 7K6T700 | Release of contracture of proximal interphalangeal joint |
| 7K6T800 | Release of contracture of distal interphalangeal joint |
| 7K6T900 | Release of contracture of elbow joint |
| 7K6Ty00 | Other specified release of contracture of joint |
| 7K6Tz00 | Release of contracture of joint NOS |
| A940.11 | Locomotor ataxia |
| E201z12 | Ataxia - hysterical |
| F11y100 | Cerebral ataxia |
| F136.00 | Idiopathic torsion dystonia |
| F137.00 | Symptomatic torsion dystonia |
| F137y00 | Other specified symptomatic torsion dystonia |
| F137z00 | Symptomatic torsion dystonia NOS |
| F138.00 | Fragments of torsion dystonia |
| F138000 | Blepharospasm |
| F138100 | Orofacial dyskinesia |
| F138111 | Tardive dyskinesia |
| F138200 | Spasmodic torticollis |
| F138z00 | Fragments of torsion dystonia NOS |
| F139.00 | Paroxysmal dyskinesia |
| F139000 | Paroxysmal non-kinesigenic dyskinesia |
| F139100 | Paroxysmal kinesigenic dyskinesia |
| F13A.00 | Paroxysmal dystonia |
| F13B.00 | Myoclonic dystonia |
| F13X.00 | Dystonia, unspecified |
| F140.00 | Friedreich's ataxia |
| F142000 | Marie's cerebellar ataxia |
| F142100 | Sanger-Brown cerebellar ataxia |
| F143.00 | Cerebellar ataxia NOS |
| F145.00 | Congenital nonprogressive ataxia |
| F14y000 | Ataxia-telangiectasia |
| F22..00 | Hemiplegia |
| F22..11 | Hemiparesis |
| F220.00 | Flaccid hemiplegia |
| F221.00 | Spastic hemiplegia |
| F221.11 | Spastic foot |
| F222.00 | Left hemiplegia |
| F222.11 | Left sided weakness |
| F223.00 | Right hemiplegia |
| F223.11 | Right sided weakness |
| F22z.00 | Hemiplegia NOS |
| F24..00 | Other paralytic syndromes |
| F240.00 | Quadriplegia |
| F240.11 | Tetraplegia |
| F240000 | Flaccid tetraplegia |
| F240100 | Spastic tetraplegia |
| F242.00 | Diplegia of upper limbs |
| F243.00 | Monoplegia of lower limb |
| F244.00 | Monoplegia of upper limb |
| F245.00 | Monoplegia unspecified |
| F38y.13 | Congenital benign hypotonia |
| Fyu1000 | [X]Other hereditary ataxias |
| Fyu2400 | [X]Other dystonia |
| Fyu2A00 | [X]Dystonia, unspecified |
| G558000 | Cardiomyopathy in Friedreich's ataxia |
| J105.00 | Dyskinesia of oesophagus |
| J65yB00 | Biliary dyskinesia |
| K554100 | Contracture of cervix |
| N084.00 | Contracture of joint |
| N084000 | Joint contracture of unspecified site |
| N084100 | Joint contracture of the shoulder region |
| N084200 | Joint contracture of the upper arm |
| N084211 | Elbow joint contracture |
| N084300 | Joint contracture of the forearm |
| N084311 | Wrist joint contracture |
| N084400 | Joint contracture of the hand |
| N084500 | Joint contracture of the pelvic region and thigh |
| N084511 | Hip joint contracture |
| N084600 | Joint contracture of the lower leg |
| N084611 | Knee joint contracture |
| N084700 | Joint contracture of the ankle and foot |
| N084711 | Ankle joint contracture |
| N084800 | Joint contracture of other specified site |
| N084900 | Contracture of multiple joints |
| N084A00 | Flexion contracture-shoulder |
| N084B00 | Extension contracture-shoulder |
| N084C00 | Abduction contracture-shoulder |
| N084D00 | Adduction contracture-shoulder |
| N084E00 | Internal rotation contracture-shoulder |
| N084F00 | External rotation contracture-shoulder |
| N084G00 | Flexion contracture - elbow |
| N084H00 | Extension contracture - elbow |
| N084J00 | Pronation contracture - forearm |
| N084K00 | Supination contracture - forearm |
| N084L00 | Flexion contracture - wrist |
| N084M00 | Extension contracture of the wrist |
| N084N00 | Ulnar deviation contracture of the wrist |
| N084P00 | Radial deviation contracture of the wrist |
| N084Q00 | Flexion contracture of MCP joint |
| N084R00 | Extension contracture of MCP joint |
| N084S00 | Flexion contracture of PIP joint |
| N084T00 | Flexion contracture of DIP joint |
| N084U00 | Flexion contracture of hip |
| N084V00 | Extension contracture of hip |
| N084W00 | Abduction contracture of hip |
| N084X00 | Adduction contracture of hip |
| N084Y00 | Internal rotation contracture of hip |
| N084Z00 | External rotation contracture of hip |
| N084a00 | Flexion contracture of the knee |
| N084b00 | Equinus contracture of the ankle |
| N084c00 | Calcaneus contracture of the ankle |
| N084d00 | Flexion contracture of MTP joint |
| N084e00 | Extension contracture of MTP joint |
| N084f00 | Flexion contracture of toe IP joint |
| N084g00 | Extension contracture of toe IP joint |
| N084z00 | Contracture of joint NOS |
| N135.11 | Contracture of neck |
| N22y.11 | Abscess, contracture or calcification of bursa or tendon |
| N22y000 | Contracture of tendon sheath |
| N238.00 | Muscle contracture |
| N238000 | Contracture of pectoralis major |
| N238100 | Contracture of triceps |
| N238200 | Contracture of biceps |
| N238300 | Contracture of wrist flexor(s) |
| N238400 | Contracture of wrist extensor(s) |
| N238500 | Contracture of flexor pollicis longus |
| N238600 | Contracture of thumb extensor(s) |
| N238700 | Contracture of flexor digitorum superficialis |
| N238800 | Contracture of flexor digitorum profundus |
| N238900 | Contracture of adductor pollicis |
| N238A00 | Contracture of other intrinsic muscle(s) of hand |
| N238B00 | Contracture of iliopsoas |
| N238C00 | Contracture of rectus femoris |
| N238D00 | Contracture of adductor muscle(s) of hip |
| N238E00 | Contracture of abductor muscle(s) of hip |
| N238F00 | Contracture of hamstring(s) |
| N238G00 | Contracture of quadriceps |
| N238H00 | Contracture of tendo achilles |
| N238J00 | Contracture of tibialis anterior |
| N238K00 | Contracture of tibialis posterior |
| N238L00 | Contracture of long toe flexor(s) |
| N238M00 | Contracture of long toe extensor(s) |
| N238N00 | Contracture of intrinsic muscle(s) of foot |
| N23yC00 | Contracture of muscle |
| N367B00 | Plantar flexion contracture of TMT joint |
| N367C00 | Flexion contracture of MTP joint |
| N367D00 | Extension contracture of MTP joint |
| N367E00 | Flexion contracture of toe joint |
| Nyu9300 | [X]Other contracture of tendon (sheath) |
| Pyu9D00 | [X]Primary ciliary dyskinesia |
| Q48y200 | Congenital hypotonia |
| Q48y300 | Congenital hypertonia |
| R012000 | [D]Ataxic gait |
| R013.11 | [D]Dyskinesia |
| R013000 | [D]Ataxia NOS |
| R014000 | [D]Transient monoplegia NOS |
| SK06.00 | Volkmann's ischaemic contracture |
| Z7E4.00 | Ataxia |
| Z7E4100 | Arms ataxic |
| Z7E4200 | Legs ataxic |
| Z7E4300 | Truncal ataxia |
| Z7E4400 | Cerebellar ataxia |
| ZR1W.11 | Modified Ashworth scale for grading spasticity |
| ZRbj.00 | Quadriplegia index of function |
| ZRbj.11 | QIF - Quadriplegia index of function |
| ZS42411 | Ataxic dysarthria |
