# Supplementary material for: Association between Antibiotic Prescribing in Pregnancy and Cerebral Palsy or Epilepsy in Children Born at Term: A Cohort Study Using The Health Improvement Network
Source: PLoS One. 2015 Mar 25;10(3):e0122034. doi: 10.1371/journal.pone.0122034 (PMC4373729; doi:10.1371/journal.pone.0122034)
Supplement: S4 Appendix — (DOCX) [file pone.0122034.s004.docx]

**S4 Appendix – Read codes used to identify women with potentially neurologically-damaging infections in pregnancy.**

The code list below was created by Wilhelmine Meeraus with expert advice and input from: Prof Ruth Gilbert (paediatrician, epidemiologist); Dr Irene Petersen (statistician, primary care data epidemiologist); and Prof Irwin Nazareth (GP, epidemiologist). Women with Read codes for tests (e.g. screening tests or antibody levels) were only considered to have a potentially neurologically-damaging infection in pregnancy if the test was positive and/or indicative of current infection.

| **Code** | **Description** |
| --- | --- |
| A703.00 | Viral (serum) hepatitis B |
| 43ej.00 | VDRL titre |
| 43Jb.00 | Varicella-zoster IgG level |
| 43dV.00 | Varicella zoster IgM level |
| 43dY.00 | Varicella zoster antibody level |
| A52x.00 | Varicella with other specified complications |
| A52z.00 | Varicella with no complication NOS |
| L166z11 | UTI - urinary tract infection in pregnancy |
| 46X0.00 | Urine nitrite positive |
| 46f4.00 | Urine leucocyte test = +++ |
| 46f3.00 | Urine leucocyte test = ++ |
| 46f2.00 | Urine leucocyte test = + |
| 46X2.00 | Urine dipstick for nitrite |
| 46U4.00 | Urine culture - Proteus |
| 46U2.00 | Urine culture - mixed growth |
| 46U3.11 | Urine culture - Escherich.coli |
| 46U3.00 | Urine culture - E. Coli |
| 46U8.00 | Urine culture - Bacteria OS |
| 46H6.00 | Urine chlamydia trachomatis test positive |
| K190z00 | Urinary tract infection, site not specified NOS |
| K190.00 | Urinary tract infection, site not specified |
| L166800 | Urinary tract infection complicating pregnancy |
| K190500 | Urinary tract infection |
| A010.00 | Typhoid fever |
| A172200 | Tuberculous adenitis |
| A172000 | Tuberculous - cervical lymphadenitis |
| A121.00 | Tuberculosis of intrathoracic lymph nodes |
| A170100 | Tuberculosis - lupus vulgaris |
| A1...00 | Tuberculosis |
| AD10100 | Trichomonal vulvovaginitis |
| AD10111 | Trichomonal vaginitis |
| A97..12 | Treponemal infection |
| 9kF8.11 | Treatment of recurrent genital herpes |
| AD0..00 | Toxoplasmosis |
| 43WI.00 | Toxoplasma antibody level |
| 165..00 | Temperature symptoms |
| 8BAD100 | TB chemotherapy |
| 4382.00 | Syphilis titre test positive |
| A97z.00 | Syphilis NOS |
| A97..11 | Syphilis |
| A600.00 | Sylvatic yellow fever |
| 1J72.11 | Suspected swine influenza |
| 1J72.00 | Suspected influenza A virus subtype H1N1 infection |
| A9...11 | Sexually transmitted diseases |
| A341.00 | Scarlet fever - scarlatina |
| A341.11 | Scarlet fever |
| 4J23000 | Sample: salmonella cultured |
| A02z.00 | Salmonella infection NOS |
| A020.00 | Salmonella gastroenteritis |
| A56..00 | Rubella |
| 1AG..00 | Recurrent urinary tract infections |
| K190.11 | Recurrent urinary tract infection |
| K190300 | Recurrent urinary tract infection |
| H00..15 | Pyrexial cold |
| 165..12 | Pyrexia symptoms |
| A11..00 | Pulmonary tuberculosis |
| A531500 | Postzoster neuralgia |
| K190200 | Post operative urinary tract infection |
| 1W0..00 | Possible influenza A virus H1N1 subtype |
| H2z..00 | Pneumonia or influenza NOS |
| H2...00 | Pneumonia and influenza |
| A11y.00 | Other specified pulmonary tuberculosis |
| H2y..00 | Other specified pneumonia or influenza |
| A02..00 | Other salmonella infections |
| A97..00 | Other and unspecified syphilis |
| A532300 | Ophthalmic herpes zoster infection |
| A544.00 | Ophthalmic herpes simplex |
| 2524.00 | O/E-herpes labialis-cold sore |
| 2E13.00 | O/E -pyrexia of unknown origin |
| 2E13.11 | O/E - pyrexia - ? cause |
| 2524.12 | O/E - herpes labialis |
| 2E...11 | O/E - fever |
| A994.00 | Nonspecific urethritis |
| AB21.11 | Monilial vulvovaginitis |
| F007100 | Meningitis due to listeriosis |
| L175.00 | Maternal rubella in pregnancy, childbirth and the puerperium |
| L292.00 | Maternal pyrexia during labour, unspecified |
| A84..00 | Malaria |
| A871000 | Lyme disease |
| A270000 | Listeria infection |
| 43WC.00 | Listeria antibody level |
| 65Y9.11 | Latent tuberculosis |
| 16L..00 | Influenza-like symptoms |
| H271z00 | Influenza with respiratory manifestations NOS |
| H27y.00 | Influenza with other manifestations |
| H27y100 | Influenza with gastrointestinal tract involvement |
| H27z.00 | Influenza NOS |
| H27z.12 | Influenza like illness |
| 4JU0.00 | Influenza H1 virus detected |
| H2A..00 | Influenza due to Influenza A virus subtype H1N1 |
| 4J3L.00 | Influenza A virus H1N1 subtype detected |
| H2A..11 | Influenza A (H1N1) swine flu |
| H27..00 | Influenza |
| A847.00 | Induced malaria |
| A788.11 | Human immunodeficiency virus infection |
| 7P1A000 | Human immunodeficiency virus blood test |
| 43C3.11 | HIV positive |
| A541100 | Herpetic vulvovaginitis |
| A541200 | Herpetic ulceration of vulva |
| A54x300 | Herpesviral vesicular dermatitis |
| A541400 | Herpesviral infection of perianal skin and rectum |
| A532.00 | Herpes zoster with ophthalmic complication |
| A532400 | Herpes zoster ophthalmicus |
| A53z.00 | Herpes zoster NOS |
| A53..00 | Herpes zoster |
| A546.00 | Herpes simplex whitlow |
| A54..11 | Herpes simplex viral infection |
| A54z.00 | Herpes simplex no complication NOS |
| A544300 | Herpes simplex disciform keratitis |
| 43dE.00 | Herpes simplex antibody level |
| A54..00 | Herpes simplex |
| A54z.11 | Herpes labialis |
| L169300 | Herpes gestationis - not delivered |
| L169.00 | Herpes gestationis |
| 43B4.00 | Hepatitis B surface antig +ve |
| 9kZ..00 | Hepatitis B screening positive - enhanced services admin |
| A3B5.00 | Haemophilus influenzae infection |
| A98z.11 | Gonorrhoea |
| A98..00 | Gonococcal infections |
| A75..11 | Glandular fever |
| A56..11 | German measles |
| L166.00 | Genitourinary tract infections in pregnancy |
| L166000 | Genitourinary tract infection in pregnancy unspecified |
| L166z00 | Genitourinary tract infection in pregnancy NOS |
| L166300 | Genitourinary tract infection in pregnancy - not delivered |
| A541000 | Genital herpes unspecified |
| A541600 | Genital herpes simplex type 1 |
| A541z00 | Genital herpes simplex NOS |
| A541.00 | Genital herpes simplex |
| A531100 | Geniculate herpes zoster |
| H27z.11 | Flu like illness |
| 1656.00 | Feverish cold |
| 165..11 | Fever symptoms |
| 1652.00 | Feels hot/feverish |
| 46H..12 | FB in urine -microscopy |
| 2E...00 | Examination of fever |
| A074311 | Diarrhoea due to Campylobacter jejuni |
| A751.00 | Cytomegaloviral mononucleosis |
| A785X00 | Cytomegaloviral disease, unspecified |
| 43JC.00 | CMV IgM level |
| 43WT.00 | CMV antibody level |
| A707000 | Chronic viral hepatitis B with delta-agent |
| A52..00 | Chickenpox - varicella |
| A52..11 | Chickenpox |
| AB21z00 | Candidal vulvovaginitis NOS |
| AB21.00 | Candidal vulvovaginitis |
| A074300 | Campylobacter gastrointestinal tract infection |
| A074312 | Campylobacter enteritis |
| K190000 | Bacteriuria, site not specified |
| K421911 | Bacterial vaginosis |
| K421900 | Bacterial vaginitis |
| 46H..11 | Bacteria in urine O/E |
| A788100 | Asymptomatic human immunodeficiency virus infection |
| L165.00 | Asymptomatic bacteriuria in pregnancy |
| K190011 | Asymptomatic bacteriuria |
| A784.11 | Aphthous fever |
| A541500 | Anogenital herpesviral infection |
| H060C00 | Acute bronchitis due to parainfluenza virus |
| A788.00 | Acquired immune deficiency syndrome |
| Hyu0600 | [X]Influenza+oth respiratory manifestatns,virus not identifd |
| Ayu4G00 | [X]Anogenital herpes viral infection, unspecified |
| A548.00 | [X] Herpes labialis |
| R006.00 | [D]Pyrexia of unknown origin |
| R006200 | [D]Fever NOS |
| R006000 | [D]Chills with fever |
