# Supplementary material for: Association between Antibiotic Prescribing in Pregnancy and Cerebral Palsy or Epilepsy in Children Born at Term: A Cohort Study Using The Health Improvement Network
Source: PLoS One. 2015 Mar 25;10(3):e0122034. doi: 10.1371/journal.pone.0122034 (PMC4373729; doi:10.1371/journal.pone.0122034)
Supplement: S5 Appendix — (DOCX) [file pone.0122034.s005.docx]

**S5 Appendix – Read codes used to identify women with respiratory tract infections in pregnancy.**

The code list below was created by Wilhelmine Meeraus with expert advice and input from: Prof Ruth Gilbert (paediatrician, epidemiologist); Dr Irene Petersen (statistician, primary care data epidemiologist); and Prof Irwin Nazareth (GP, epidemiologist).

| **Code** | **Description** |
| --- | --- |
| 1692.00 | Swollen glands |
| 1712.00 | Dry cough |
| 1713.00 | Productive cough -clear sputum |
| 1714.00 | Productive cough -green sputum |
| 1715.00 | Productive cough-yellow sputum |
| 1716.00 | Productive cough NOS |
| 1716.11 | Coughing up phlegm |
| 1717.00 | Night cough present |
| 1719.00 | Chesty cough |
| 1719.11 | Bronchial cough |
| 16L..00 | Influenza-like symptoms |
| 171..00 | Cough |
| 171..11 | C/O - cough |
| 171..12 | Sputum - symptom |
| 171B.00 | Persistent cough |
| 171C.00 | Morning cough |
| 171J.00 | Reflux cough |
| 171Z.00 | Cough symptom NOS |
| 172..00 | Blood in sputum - haemoptysis |
| 172..11 | Blood in sputum - symptom |
| 173e.00 | Viral wheeze |
| 173e.11 | Viral induced wheeze |
| 1C3..00 | Earache symptoms |
| 1C32.00 | Unilateral earache |
| 1C33.00 | Bilateral earache |
| 1C34.00 | Irritation of ear |
| 1C3Z.00 | Earache symptom NOS |
| 1C4..00 | Ear discharge symptoms |
| 1C42.00 | Ear discharge present |
| 1C43.00 | Blood discharge from ear |
| 1C9..00 | Sore throat symptom |
| 1C9..11 | Throat soreness |
| 1C92.00 | Has a sore throat |
| 1C93.00 | Persistent sore throat |
| 1C9Z.00 | Sore throat symptom NOS |
| 1CA..00 | Hoarseness symptom |
| 1CA..11 | Hoarseness - throat symptom |
| 1CA2.00 | Hoarse |
| 1CA2.11 | Voice hoarseness |
| 1CB3.00 | Throat pain |
| 1CB3.11 | Pain in throat |
| 1CC..00 | Blocked sinuses |
| 1J72.00 | Suspected influenza A virus subtype H1N1 infection |
| 1J72.11 | Suspected swine influenza |
| 1W0..00 | Possible influenza A virus H1N1 subtype |
| 2D24.00 | O/E -nasal disch.-mucopurulent |
| 2D73.11 | O/E - mastoid tender |
| 2DB..11 | O/E - tonsils enlarged |
| 2DB3.00 | O/E - tonsils mod. enlarged |
| 2DB4.00 | O/E - tonsils grossly enlarged |
| 2DB6.00 | O/E - follicular tonsillitis |
| 2DB7.00 | O/E - exudate on tonsils |
| 2DC2.00 | O/E - granular pharyngitis |
| 2DC3.00 | Inflamed throat |
| 4E23.00 | Sputum: mucopurulent |
| 4E29.00 | Green sputum |
| 4J3L.00 | Influenza A virus H1N1 subtype detected |
| 4JU0.00 | Influenza H1 virus detected |
| 65VA.00 | Notification of whooping cough |
| 65VH.00 | Notification of mumps |
| 65Y9.11 | Latent tuberculosis |
| 8BAD100 | TB chemotherapy |
| A1...00 | Tuberculosis |
| A11..00 | Pulmonary tuberculosis |
| A11y.00 | Other specified pulmonary tuberculosis |
| A121.00 | Tuberculosis of intrathoracic lymph nodes |
| A170100 | Tuberculosis - lupus vulgaris |
| A172000 | Tuberculous - cervical lymphadenitis |
| A172200 | Tuberculous adenitis |
| A31..00 | Other mycobacterial diseases |
| A33..00 | Whooping cough |
| A340.00 | Streptococcal sore throat |
| A340100 | Streptococcal laryngitis |
| A340200 | Streptococcal pharyngitis |
| A340300 | Streptococcal tonsillitis |
| A3B5.00 | Haemophilus influenzae infection |
| A3B7.00 | Pseudomonas infection |
| A3BXA00 | Mycoplasma pneumoniae [PPLO] cause/dis classifd/oth chaptr |
| A3By300 | Mycoplasma infection |
| A552.00 | Postmeasles otitis media |
| A72..00 | Mumps |
| A72z.11 | Epidemic parotitis |
| A785.11 | Salivary gland virus disease |
| A790.00 | Adenovirus |
| A79A.00 | Respiratory syncytial virus infection |
| AA1..00 | Vincent's angina |
| AB20.13 | Pharyngeal candidiasis |
| AB2y100 | Candidal otitis externa |
| F501.00 | Infective otitis externa |
| F501000 | Unspecified infective otitis externa |
| F501100 | Acute infective otitis externa |
| F501111 | Abscess, external ear |
| F501112 | Cellulitis, external ear |
| F501200 | Acute infection of pinna |
| F501711 | Impetigo - otitis externa |
| F501800 | Furunculosis of external auditory meatus |
| F501900 | Other acute external ear infections |
| F501F00 | Chronic infective otitis externa NOS |
| F501z00 | Infective otitis externa NOS |
| F52..00 | Suppurative and unspecified otitis media |
| F520.00 | Acute suppurative otitis media |
| F520000 | Acute suppurative otitis media tympanic membrane intact |
| F524.00 | Purulent otitis media NOS |
| F52z.11 | Infection ear |
| F53..00 | Mastoiditis and related conditions |
| F53z.00 | Mastoiditis NOS |
| F540.00 | Acute myringitis without otitis media |
| F563500 | Viral labyrinthitis |
| H0...00 | Acute respiratory infections |
| H00..00 | Acute nasopharyngitis |
| H00..11 | Common cold |
| H00..12 | Coryza - acute |
| H00..13 | Febrile cold |
| H00..14 | Nasal catarrh - acute |
| H00..15 | Pyrexial cold |
| H00..16 | Rhinitis - acute |
| H01..00 | Acute sinusitis |
| H01..11 | Sinusitis |
| H010.00 | Acute maxillary sinusitis |
| H011.00 | Acute frontal sinusitis |
| H012.00 | Acute ethmoidal sinusitis |
| H014.00 | Acute rhinosinusitis |
| H01y000 | Acute pansinusitis |
| H01z.00 | Acute sinusitis NOS |
| H02..00 | Acute pharyngitis |
| H02..11 | Sore throat NOS |
| H02..12 | Viral sore throat NOS |
| H02..13 | Throat infection - pharyngitis |
| H021.00 | Acute phlegmonous pharyngitis |
| H023.00 | Acute bacterial pharyngitis |
| H024.00 | Acute viral pharyngitis |
| H02z.00 | Acute pharyngitis NOS |
| H03..00 | Acute tonsillitis |
| H03..11 | Throat infection - tonsillitis |
| H03..12 | Tonsillitis |
| H030.00 | Acute erythematous tonsillitis |
| H031.00 | Acute follicular tonsillitis |
| H032.00 | Acute ulcerative tonsillitis |
| H035.00 | Acute bacterial tonsillitis |
| H036.00 | Acute viral tonsillitis |
| H037.00 | Recurrent acute tonsillitis |
| H03z.00 | Acute tonsillitis NOS |
| H04..00 | Acute laryngitis and tracheitis |
| H040.00 | Acute laryngitis |
| H040200 | Acute catarrhal laryngitis |
| H040300 | Acute phlegmonous laryngitis |
| H040w00 | Acute viral laryngitis unspecified |
| H040z00 | Acute laryngitis NOS |
| H041.00 | Acute tracheitis |
| H041z00 | Acute tracheitis NOS |
| H042.00 | Acute laryngotracheitis |
| H042.11 | Laryngotracheitis |
| H042z00 | Acute laryngotracheitis NOS |
| H043z00 | Acute epiglottitis NOS |
| H044.00 | Croup |
| H05..00 | Other acute upper respiratory infections |
| H050.00 | Acute laryngopharyngitis |
| H051.00 | Acute upper respiratory tract infection |
| H052.00 | Pharyngotracheitis |
| H054.00 | Recurrent upper respiratory tract infection |
| H055.00 | Pharyngolaryngitis |
| H05y.00 | Other upper respiratory infections of multiple sites |
| H05z.00 | Upper respiratory infection NOS |
| H05z.11 | Upper respiratory tract infection NOS |
| H05z.12 | Viral upper respiratory tract infection NOS |
| H06..00 | Acute bronchitis and bronchiolitis |
| H060.00 | Acute bronchitis |
| H060300 | Acute purulent bronchitis |
| H060500 | Acute tracheobronchitis |
| H060A00 | Acute bronchitis due to mycoplasma pneumoniae |
| H060C00 | Acute bronchitis due to parainfluenza virus |
| H060w00 | Acute viral bronchitis unspecified |
| H060z00 | Acute bronchitis NOS |
| H061.00 | Acute bronchiolitis |
| H062.00 | Acute lower respiratory tract infection |
| H06z.00 | Acute bronchitis or bronchiolitis NOS |
| H06z000 | Chest infection NOS |
| H06z011 | Chest infection |
| H06z100 | Lower resp tract infection |
| H06z111 | Respiratory tract infection |
| H06z200 | Recurrent chest infection |
| H07..00 | Chest cold |
| H0z..00 | Acute respiratory infection NOS |
| H120400 | Chronic infective rhinitis |
| H121400 | Pharyngitis keratosa |
| H130.12 | Maxillary sinusitis |
| H131.11 | Frontal sinusitis |
| H135.00 | Recurrent sinusitis |
| H13y100 | Pansinusitis |
| H141100 | Hypertrophy of tonsils alone |
| H14y500 | Caseous tonsillitis |
| H14y600 | Lingular tonsillitis |
| H15..00 | Peritonsillar abscess - quinsy |
| H15..11 | Quinsy |
| H160400 | Laryngitis sicca |
| H1y1z13 | Sinus congestion |
| H1y1z14 | Nasal infection |
| H2...00 | Pneumonia and influenza |
| H20..00 | Viral pneumonia |
| H201.00 | Pneumonia due to respiratory syncytial virus |
| H21..00 | Lobar (pneumococcal) pneumonia |
| H21..11 | Chest infection - pneumococcal pneumonia |
| H220.00 | Pneumonia due to klebsiella pneumoniae |
| H224.00 | Pneumonia due to staphylococcus |
| H22z.00 | Bacterial pneumonia NOS |
| H233.00 | Chlamydial pneumonia |
| H25..00 | Bronchopneumonia due to unspecified organism |
| H26..00 | Pneumonia due to unspecified organism |
| H260.00 | Lobar pneumonia due to unspecified organism |
| H261.00 | Basal pneumonia due to unspecified organism |
| H262.00 | Postoperative pneumonia |
| H27..00 | Influenza |
| H271z00 | Influenza with respiratory manifestations NOS |
| H27y.00 | Influenza with other manifestations |
| H27y100 | Influenza with gastrointestinal tract involvement |
| H27z.00 | Influenza NOS |
| H27z.11 | Flu like illness |
| H27z.12 | Influenza like illness |
| H28..00 | Atypical pneumonia |
| H2A..00 | Influenza due to Influenza A virus subtype H1N1 |
| H2A..11 | Influenza A (H1N1) swine flu |
| H2y..00 | Other specified pneumonia or influenza |
| H2z..00 | Pneumonia or influenza NOS |
| H5yy.11 | Respiratory infection NOS |
| Hyu0600 | [X]Influenza+oth respiratory manifestatns,virus not identifd |
| R064.00 | [D]Abnormal sputum |
| R064100 | [D]Sputum abnormal - colour |
| R153100 | [D]Positive culture findings in sputum |
